# High-throughput discovery and characterisation of pentafluorobenzene sulfonamide modifiers of Aurora A kinase

**DOI:** 10.1039/d5cb00290g

**Published:** 2026-03-16

**Authors:** Julian Chesti, Jennifer A. Miles, Lawrence J. Collins, Hamish A. McCallum, Martina Foglizzo, Mohd Syed Ahangar, Elton Zeqiraj, Richard Bayliss, Stuart L. Warriner, Megan H. Wright, Adam Nelson

**Affiliations:** a School of Chemistry, University of Leeds Leeds LS2 9JT UK a.s.nelson@leeds.ac.uk; b Astbury Centre for Structural Molecular Biology, University of Leeds Leeds LS2 9JT UK; c School of Molecular and Cellular Biology, University of Leeds Leeds LS2 9JT UK

## Abstract

Covalent modification can enable understanding and modulation of protein function, and the identification of new therapeutic opportunities. A “direct to biology” workflow was developed that harnesses sulfonylation as a connective reaction for the synthesis of diverse sets of reactive fragments. The workflow expanded the diversity of accessible reactive fragment sets, and facilitated the discovery of pentafluorobenzene sulfonamides that modify Aurora A kinase, NEK7 kinase, and UbcH5B. Characterisation of several of the Aurora A-modifying reactive fragments revealed both their modification rates and sites. Furthermore, Cys247, a residue typically buried in Aurora A crystal structures, was identifed as a modifable residue. These findings underscore the importance of protein dynamics in determining cysteine reactivity and highlight the utility of reactive fragment sets for identifying cryptic pockets. Sulfonylation is therefore a useful complement to amide formation in “direct to biology” workflows aimed at identifying novel opportunities for targeted protein modification.

## Abstract 2

Covalent modification, by substitution of pentafluorobenzene sulfonamides, can enable understanding and modulation of protein function, and the identification of new therapeutic opportunities.

## Graphical abstract

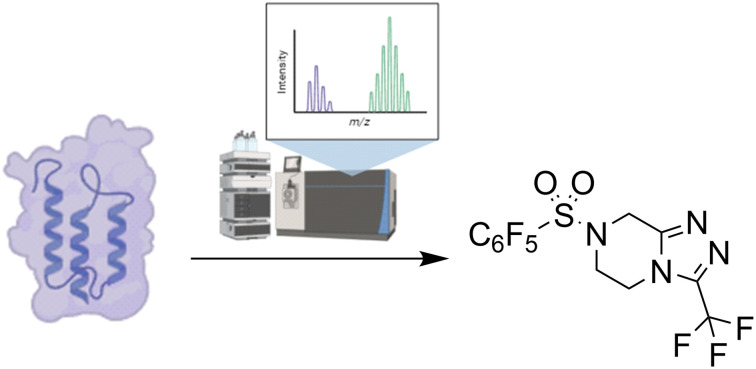

## Introduction

Covalent modification of proteins has enjoyed a recent resurgence as a drug discovery strategy.^1,2^ Covalent modifiers can also drive identification of new therapeutic opportunities, and demonstrate ligand-protein engagement in a cellular context.^3–6^ Furthermore, targeted covalent modification can rewire protein function, for example by modulating catalysis,^7^ interactions,^8^ localisation^9^ and degradation.^10^

The discovery of effective reagents for targeted protein modification can require multiple resource- and time-intensive design-make-test cycles. In broad terms, targeted covalent modifiers may be discovered either by attaching an electrophilic warhead to a high-affinity ligand (“ligand first”), or by developing a reactive fragment or other covalent modifier (“electrophile first”).^11–13^ The “electrophile first” approach was exemplified, initially using tethered fragments,^14^ in the discovery of covalent modifiers of the G12C variant of KRAS.^15^ Covalent modifiers, including reactive fragments, have been exploited widely to enable protein kinase drug discovery and chemical biology.^16–19^

Recently, high-throughput “direct-to-biology” workflows have been established to enable the integrated plate-based synthesis and evaluation of arrays of reactive fragments,^20–22^ including on a nanoscale.^23^ “Direct-to-biology” workflows enable researchers to evaluate arrays of compounds rapidly, without time-consuming purification of each compound. The approach can enable efficient exploration of chemical space and assessment of target tractability, and provide starting points for the discovery of chemical tools and drugs. Within this context, reactive fragments are prepared by connecting, generally by amide formation, pairs of plated building blocks: a building block that introduces diverse functionality for protein recognition, and a building block bearing an electrophilic warhead. As with activity-directed synthesis,^24^ the resulting arrays of reaction products are screened without purification. The approach has been used to discover modifiers of many proteins that bear warheads including α-chloro amides^25,26^ sulfonyl fluorides^27^ and tetrazoles.^28^ The direct-to-biology approach has also been used to discover covalent modifiers and photoreactive probes for a wide range of targets,^20,25–28^ as well as non-covalent molecular glues.^29^

**Fig. 1:**
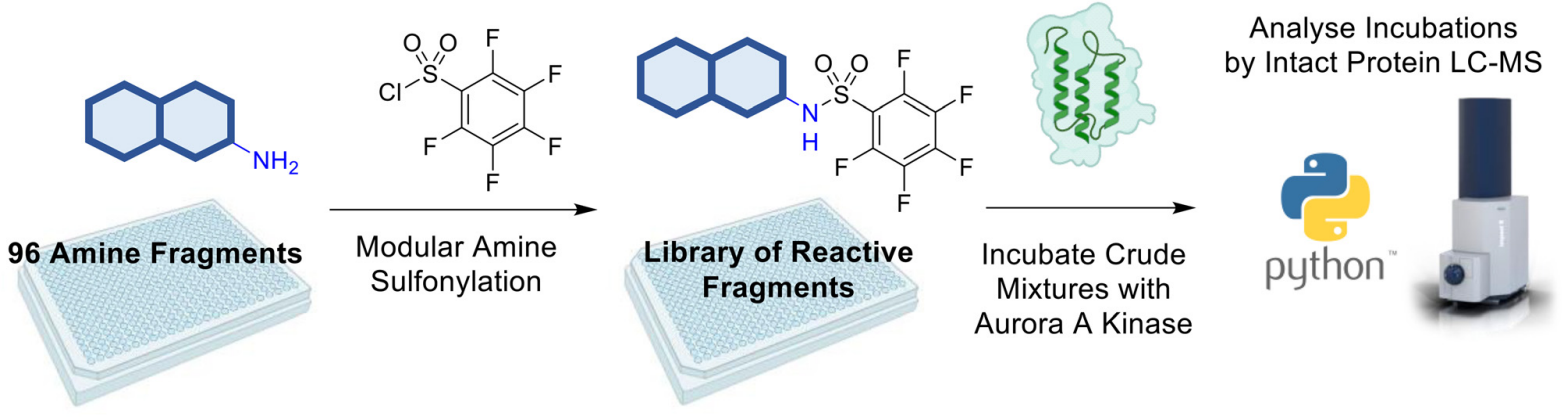
Overview of envisaged “direct to biology” approach for the discovery of reactive fragment modifiers of Aurora A kinase. Initially, arrays of reactive fragments would be prepared by reacting diverse amines with fluorinated benzenesulfonamides. The crude products would be directly screened for covalent modification of Aurora A kinase by mass spectrometry.

In this paper, we show that sulfonamide formation, in addition to amide formation, may be harnessed as a connective reaction in “direct to biology” workflows (Fig. 1). We thereby demonstrate that sulfonamide formation may enable the discovery of reactive fragment modifiers of Aurora A kinase. Aurora A kinase is a serine/threonine kinase that plays a key role in cell division, and is over-expressed in many cancers.^30–32^ A wide range of inhibitors of Aurora A kinase, principally ATP-competitive reversible inhibitors, has been discovered, and alisertib^33^ recently received orphan drug designation for the treatment of small cell lung cancer. In addition, covalent modifiers that target a catalytic lysine residue have also been discovered for Aurora A kinase.^34^ Finally, we describe the validation and characterisation of the reactive fragment hits that were discovered.

## Results and discussion

### High-throughput discovery of covalent modifiers

**Fig. 2:**
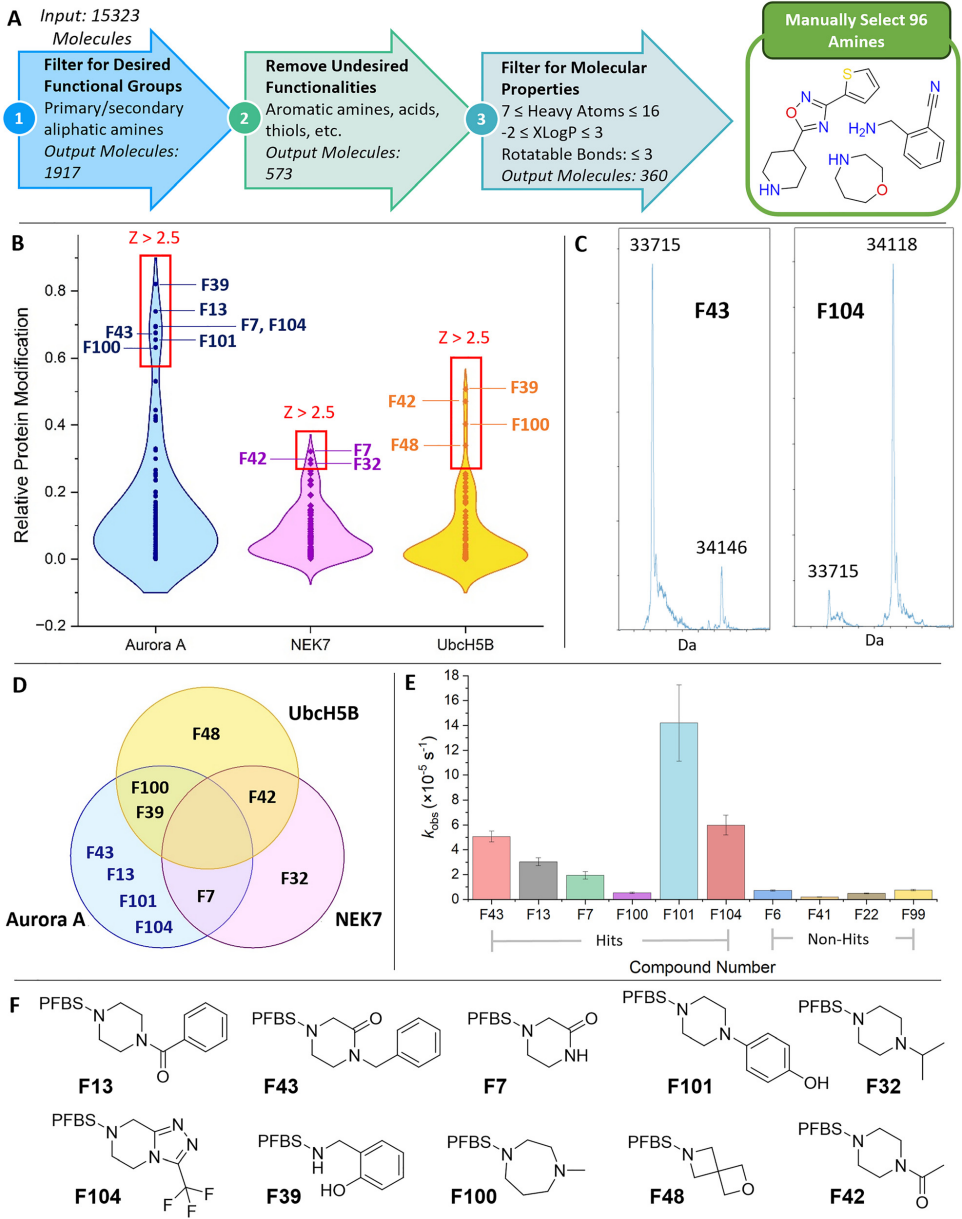
High-throughput discovery of reactive fragment hits. Panel A: Design of a diverse set of amine building blocks (SI). Panel B: Screen of pentafluorobenzene sulfonamides against Aurora A (fragment concentration: 20 µM), NEK7 (20 µM) and UbcH5B (200 µM). Panel C: Deconvoluted mass-spectra of Aurora A kinase (2 µM; unmodified: 33715 Da) following incubation with purified samples of F43 and F104 (20 µM) for 24 h. Panel D: Venn diagram showing the distribution of hits for the three proteins. Panel E: *k*_obs_ values for Aurora A kinase modification with hits and selected non-hits (20 µM). Panel F: Structures of reactive fragment hits. PFBS, perfluorobenzenesulfonyl.

Initially, we designed a set of 96 diverse amine building blocks. Here, a computational workflow, implemented using KNIME,^35^ was used to identify, from our laboratory inventory, primary and secondary amines with appropriate functionality and molecular properties, from which 96 diverse building blocks were manually selected (Fig. 2, Panel A and SI Fig. S4). Anilines and heteroaryl amines were excluded because we had shown that their reactions were not clean under the conditions used (SI, Figs. S2 and S3).

The synthesis of the array of reactive fragments was performed in glass microscale vials within bespoke plastic plates. In a limited array with 24 diverse amines, we had previously shown that only pentafluorobenzene sulfonamides, and not 2,3,5,6-tetrafluoro, 2,4,6-trifluoro or 2,6-difluoro analogues, modified Aurora A kinase (SI, Fig. S1). This finding was in line with previous investigations into the reactivity and viability of fluorinated benzenesulfonamide and (hetero)arene warheads.^36,37^ At this stage, we also assessed the inherent reactivity of exemplar fragments with a cysteine and a lysine derivative (SI, Fig. S14). Accordingly, the array was prepared by pipetting, sequentially, stock solutions of triethylamine (30 µl of a 66 mM solution in MeCN), each amine building block (30 µl of a 60 mM solution in MeCN) and pentafluorobenzenesulfonyl chloride (30 µl of a 60 mM solution in MeCN). Unfortunately, we had found that these sulfonylation reactions were incompatible with DMSO as solvent. The reactions were therefore stirred overnight, the solvent removed, and DMSO added to give, assuming complete conversion, an array of reactive fragments as 20 mM stock solutions in DMSO. The array of reactive fragments was diluted in buffer and incubated (final concentration: 20 µM) for 24 h with truncated (residues 118–389), TPX2-fused Aurora A kinase (final concentration: 2 µM) in TRIS buffer (with 1% DMSO) in a 384-well plate. To assess selectivity, the fragments were also screened against NEK7,^38^ another protein kinase, and UbcH5B, an E2 ubiquitin-conjugating enzyme.††Four mutations (C21S, S22R, C107S, C111S) had been introduced to reduce protein aggregation and modulate ubiquitin binding (see SI).,^39^ For UbcH5B, a higher concentration (200 µM) of reactive fragments was used following a preliminary smaller-scale screen. The plate of protein modification reactions was analysed by mass spectrometry, with automated extraction, processing and visualisation of the resulting data. Specifically, the relative proportions of un-, singly, doubly and triply modified protein were determined. Notably, there was no evidence of sulfonylation of any of the proteins, suggesting that any residual pentafluorobenzenesulfonyl chloride had been quenched before incubation.

For each of the three proteins, reactive fragments that resulted in a proportion of singly modified protein that was >2.5 standard deviations higher than the mean for the whole array (*i.e. Z* > 2.5) were designated as hits (Fig. 2, Panels B and D and SI, Fig. S5); notably, none of these hits resulted in >2% doubly or triply modified protein. Unique reactive fragment hits were found for Aurora A kinase (F13, F43, F101 and F104), NEK7 (F32) and UbcH5B (F48); notably, there were no hits that modified all three proteins.

To enable hit validation, we resynthesised and purified six hits for Aurora A kinase‡‡A clean sample of **F39** was not obtained. (Fig. 2 Panel B), together with four reactive fragments that were not hits for any of the proteins (SI, Fig. S6). The reactions between the purified compounds (20 µM) and Aurora A kinase (2 µM), were followed by mass spectrometry, and time-dependent conversions, performed in triplicate, were determined over 24 h (Fig. 2 Panel C and SI, Fig. S7). Notably, we have shown that two exemplar fragments do not decompose over this timescale under the same conditions. Five of the hits (F7, F13, F43, F101 and F104) were found to have higher observed rates, *k*_obs_ of protein modification than the four compounds that had not been identified as hits (Fig. 2, Panel E).

### Hit characterisation

The two hits with the fastest rates of protein labelling at 20 µM (*i.e.*F101 and F104) were selected for more detailed kinetic studies. Irreversible enzyme inhibitors may be characterised in terms of *K*_I_, an equilibrium constant capturing an initial non-covalent association, and *k*_inact_, a rate constant capturing the irreversible covalent modification event.^40^ Given that chemical modification of Aurora A may not result in inhibition, we have used the directly analogous parameters *K*_D_ and *k*_modify_ to describe the kinetics of its covalent modification. Observed rates of Aurora A modification, *k*_obs_, were determined by time-dependent mass spectrometry at a range of concentrations of reactive fragment (12.5–200 µM); at 200 µM, both fragments were found to be soluble in 1% DMSO in TRIS buffer. For F104, *k*_obs_ was ∼7 × 10^−5^ s^−1^ at all concentrations; this suggested that saturation was observed in all cases *i.e. K*_D_ < 10 µM and *k*_modify_ was ∼7 × 10^−5^ s^−1^ (see SI, Fig. S13). In contrast, at higher concentrations of F101 (*e.g.* 200 µM), the appearance of phosphorylated, but unmodified, protein was observed in addition to the expected covalently-modifed Aurora A (SI, Fig. S8). This more complex behaviour presumably stems from autophophorylation by adventitious ATP, and precluded more complete kinetic characterisation of protein modification, and was not studied further.

**Fig. 3:**
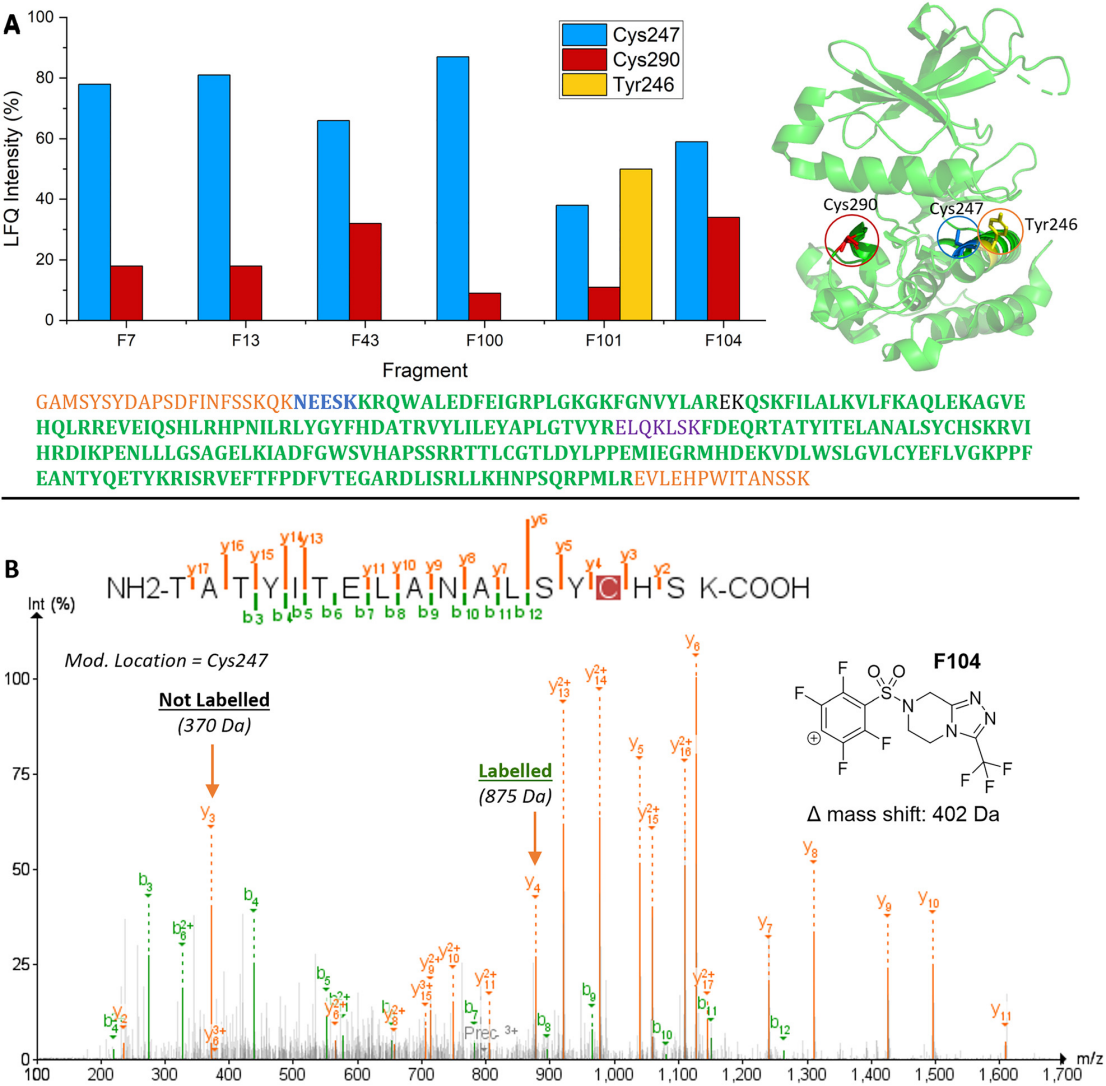
Identification of sites of protein modification by trypsin digestion and LC-MS/MS analysis of the resulting peptides. Panel A: Relative intensities of modified peptides and basis for the assignment of labelling sites. Sequence coverage is illustrated by colour: samples treated with F13, F101 and F104 (99%, coloured); F43 (95%, green and orange); and F7 and F100 (86%, green, and purple). Panel B: Example of the identification of a modification site (Cys247) by fragmentation of an exemplar modified peptide.

The modification sites of the reactive fragment hits were determined by proteolysis and LC-MS/MS analysis of the resulting peptides (SI, Fig. S10). Initially, the reactive fragment hits (200 µM) were incubated with Aurora A kinase (20 µM) for 2–5 h, and the resulting modified protein samples analysed by mass spectrometry. After sequential treatment with tris(2-carboxyethyl)phosphine (TCEP) and iodoacetamide, the protein samples were digested by treatment with trypsin/LysC, and the resulting peptides analysed by LC-MS/MS (Fig. 3 and SI, Fig. S10). Analysis of the relative intensities of the modified peptides, and their fragmentation patterns, revealed that F7, F13, F43, F100, F101 and F104) modified Cys247 and Cys290; one fragment (F101) was also observed to modify Tyr246. Although multiple modification sites were identified, it is notable that none of these hits resulted in >2% double (or triple) modification of Aurora A kinase (SI, Fig. S7).

## Discussion

**Fig. 4:**
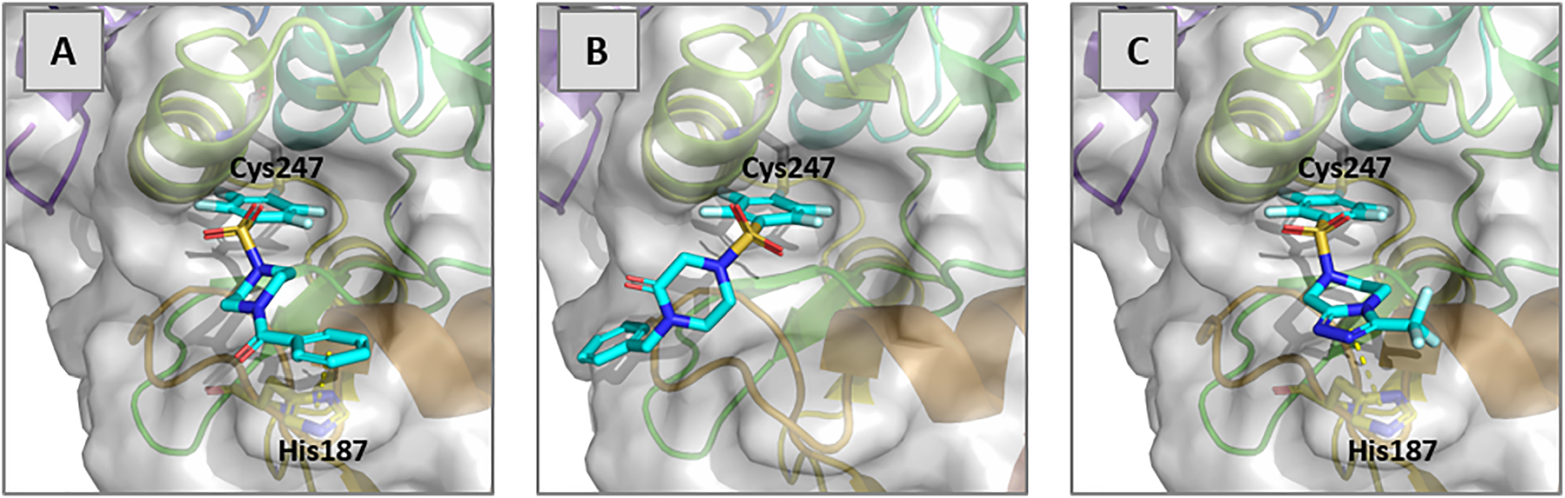
Structures of Aurora A kinase following covalent docking of selected reactive fragments to Cys247 of Aurora A kinase (PDB: 5ORL): F13 (Panel A), F43 (Panel B) and F104 (Panel C). Schödinger Maestro v. 14.1.138 was used.

Modification of established “direct to biology” workflows enabled sulfonylation, rather than amide formation, to be harnessed as a connective reaction for the synthesis of arrays of reactive fragments. This increases the diversity and flexibility of reactive fragments that may be accessed, and enables the introduction of warheads with complementary reactivity (*e.g.* to acrylamides and α-chloro acetamides). The modified workflow enabled discovery of reactive fragment hits bearing a pentafluorobenzenesulfonyl group, a warhead that has previously been shown to be viable for targeted protein modification.^36,37,41^ The Aurora A kinase construct used had three cysteine residues: Cys247 (which is buried), Cys290 (on the activation loop) and Cys319 (which is also buried).§§Solvent accessibility of cysteines in PDB structure 6VPG was assessed using FANTOM (ref. 42).^42^ Remarkably, five of the validated reactive fragment hits were found to modify Cys247, a residue that is buried in the structure^43^ of the unmodified protein (*e.g.* in complex with an ADP analogue: PDB 6VPG). Notably, a isothiazolone had also been observed, unexpectedly, to modify this residue in a previous high-throughput crystallography fragment screen of Aurora A.^43^ We have used covalent docking^44^ (based on PDB structure 5ORL in which Cys247 is covalently modified) to rationalise the modification of Cys247; these experiments suggested that F13 and F104 may engage in π/π stacking interactions with His187 (Fig. 4 and SI, Fig. S11).

**Fig. 5:**
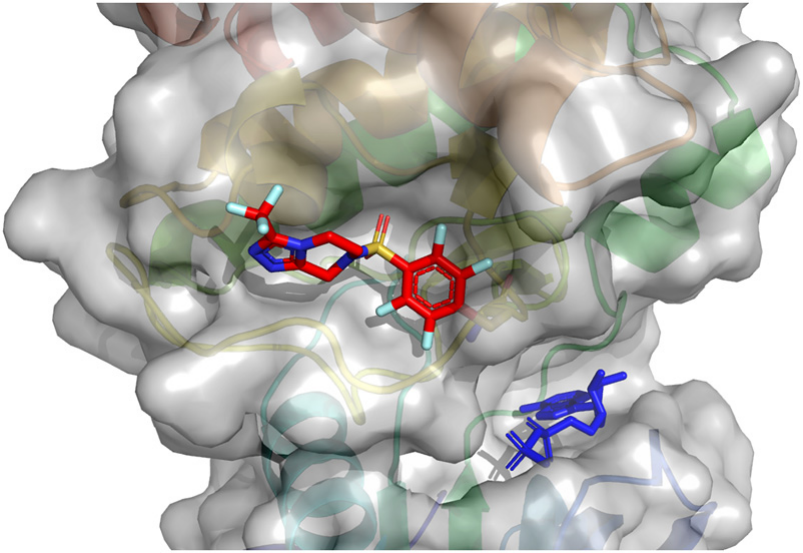
X-ray crystal structure of Aurora A kinase modified on Cys290 with reactive fragment F104 (red) and ADP (blue) (PDB: 9SUY).

We also crystallised the F104-modified D274N variant¶¶This variant of TPX2-fused Aurora A kinase could be expressed in higher yield which aided crystallisation experiments. of Aurora A in the presence of ADP. This enabled determination of the structure of the Cys290-modified protein (PDB: 9SUY) which had crystallised preferentially (Fig. 5), which overlays extremely well with the covalently-docked structure (SI, Fig. S11 Panel D). Overall, the most promising fragment hit that was identified was F104, which did not decompose in buffer over 20 h (SI, Fig. S12), and whose *k*_modify_/*K*_D_ (which is at least 7 M^−1^ s^−1^) is comparable with other reactive fragments discovered using a “direct-to-biology” approach.^25–28^F104 may provide a useful starting point for the discovery of a targeted covalent modifier of Aurora A kinase.

More generally, our results highlight the importance of protein dynamics, which are not captured crystallographically, in determining cryptic sites that are susceptible to covalent modification. In this context, sets of reactive fragments are valuable in enabling experimental profiling of cysteine reactivity in protein targets. Such experiments can enable identification of tractable sites for covalent modification, including cryptic sites, that can enable chemical biology and drug discovery.

## Conclusions

We have developed a “direct to biology” workflow that enables sulfonylation to be harnessed as a connective reaction for the synthesis of arrays of reactive fragments. The identification of sulfonylation as a suitable connective reaction increases both the diversity and flexibility of reactive fragment sets that are accessible *via* amide formation. Our workflow was exploited in the discovery of pentafluorobenzene sulfonamide reactive fragments that modify three protein targets: Aurora A kinase, NEK7 kinase and UbcH5B. We characterised several of the reactive fragments that modify Aurora A kinase by determining both modification rates and sites. The fragments identified – particularly F104 – may provide a useful starting point for the discovery of targeted covalent modifiers of Aurora A kinase. Surprisingly, it was found that Cys247, which is buried in many Aurora A crystal structures, is amenable to covalent modification. We conclude that protein dynamics play a critical role in determining cysteine reactivity, and that sets of reactive fragments are valuable for profiling cysteine reactivity and identifying cryptic sites that may be targeted by small molecules. Overall, sulfonylation may usefully complement amide formation as a connective reaction for “direct to biology” workflows, and may enable the identification of opportunities for the targeted covalent modification of other protein targets.

## Experimental

### High-throughput discovery of covalent modifiers

Reaction arrays were executed in a custom-made PTFE 96-well plate (8 × 12) with inserts for borosilicate glass vials (vial volume = 750 µL, vial dimensions = 8 × 30 mm, CV-2100-0830 Chemglass). Stock solutions of triethylamine (66 mM in MeCN), perfluorobenzenesulfonyl chloride (60 mM in MeCN) and the amine building blocks (60 mM in MeCN) were prepared. Stock solutions of amines that were salts were dissolved in a solution of triethyl amine (60 mM in MeCN). A magnetic follower was added to each vial, followed by a solution of triethylamine (30 µL, 66 mM in MeCN). A solution of the corresponding amine substrate was then added (30 µL, 60 mM in MeCN), followed by a solution of perfluorobenzenesulfonyl chloride (30 µL, 60 mM in MeCN). All reagents were transferred to glass vials using pipettes. The final concentration of components in each well was: triethylamine (22 mM, 1.2 equiv; higher when the amine had been a salt); amine substrate (20 mM, 1 equiv) and perfluorobenzenesulfonyl chloride (20 mM, 1 equiv) in a total reaction volume of 90 µL of MeCN. The glass vials were capped and the reactions were allowed to stir at room temperature for 24 h. The solvent was then removed under reduced pressure in a desiccator. The remaining crude residues were dissolved in 90 µL of DMSO so that the final concentration of product was 20 mM (assumed full conversion of starting material to product). If necessary, these DMSO solutions were stored at −20 °C prior to screening.

The crude reaction mixtures in DMSO (assumed to be 20 mM) were diluted tenfold in DMSO in a separate PTFE custom made 96-well plate (8 × 12) with inserts for borosilicate glass vials (vial volume = 750 µL, vial dimensions = 8 × 30 mm, CV-2100-0830 Chemglass). The resulting solutions (assumed to be 2 mM) were diluted a further tenfold in freshly made TRIS buffer (tris(hydroxymethyl)aminomethane (25 mM), NaCl (150 mM) and MgCl_2_ (5 mM) in H_2_O (100 mL), pH 7.5) in a 384-well PerkinElmer plate to make the desired working solutions (200 µM, 10% DMSO). Working solutions of crude reaction mixtures (3 µL) were then added to a Corning 384-well plate (part no. 4514) containing Aurora A kinase (15 µL, 4 µM) and TRIS buffer (12 µL). The final volume of the incubation mixture was 30 µL with protein (2 µM) and reactive fragment (assumed to be 20 µM, 1% DMSO). The plate was then sealed with adhesive foil and the mixtures were allowed to stand for 24 h at room temperature (∼20 °C). Each reaction was then injected (3 µL) onto a high-resolution mass spectrometer. Maximum entropy deconvolution methods were used as part of the downstream processing of acquired spectra to determine the proportion of unlabelled and labelled protein. A Python script was utilised to rapidly extract, process and visualise MS data acquired from protein labelling reactions.

## Author contributions

The contributions for the authors were conceptualisation (MHW, AN), investigation (JC, JAM), methodology (JC, HAC), software (LJC, SLW), provision of resources (JAM, MF, MSA), supervision (RB, EZ, SLW, MHW, AN), funding acquisition (EZ, SLW, RB, MHW, AN) and writing (original draft: JC, AN; reviewing and editing: all authors).

## Conflicts of interest

There are no conflicts to declare.

## Supplementary Material

CB-OLF-D5CB00290G-s001

## Data Availability

Supplementary information(SI): Supplementary figures; details of proteins used; experimental and computational methods; compound characterisation data; and data for site mapping and kinetic characterisation of protein modification. See DOI: https://doi.org/10.1039/d5cb00290g. The molecular coordinates and experimental data associated with the X-ray crystal structure of Aurora A kinase modified with reactive fragment F104 have been deposited in the Protein Data Bank (PDB) with accession code 9SUY. The code for the automated deconvolution of mass spectrometry data for protein modification reactions can be found at https://github.com/lawrencecollins/BafPipe.
