# Supplementary material for: High-throughput discovery and characterisation of pentafluorobenzene sulfonamide modifiers of Aurora A kinase
Source: RSC Chem Biol. 2026 Mar 16;7(5):862–9. doi: 10.1039/d5cb00290g (PMC12989823; doi:10.1039/d5cb00290g)
Supplement: CB-007-D5CB00290G-s001 [file CB-007-D5CB00290G-s001.pdf]

Supplementary Information (SI) for RSC Chemical Biology.
This journal is © The Royal Society of Chemistry 2026

SUPPLEMENTARY INFORMATION

High-throughput discovery and characterisation of pentafluorobenzene sulfonamide modifiers of Aurora A kinase

Julian Chesti,[a,b] Jennifer A. Miles,[b,c] Lawrence J. Collins,[b,c] Hamish A. McCallum,[a] Martina Foglizzo,[b,c] Mohd Syed Ahangar,[b,c] Elton Zeqiraj,[b,c] Richard Bayliss,[b,c] Stuart L. Warriner,[a,b] Megan H. Wright[a,b] and Adam Nelson*[a,b]

a. *School of Chemistry, University of Leeds, Leeds, LS2 9JT, UKS12*
b. *Astbury Centre for Structural Molecular Biology, University of Leeds, Leeds, LS2 9JT, UK*
c. *School of Molecular and Cellular Biology, University of Leeds, Leeds, LS2 9JT, UK*

Contents

1. SUPPLEMENTARY FIGURES 3
2. BIOLOGY EXPERIMENTAL 17
2.1 PROTEIN SEQUENCES 17
2.1.1 PROTEIN EXPRESSION 18
2.2 PROTEIN LABELLING EXPERIMENTS 19
2.2.1 DISCOVERY OF REACTIVE FRAGMENT HITS USING A DIRECT-TO-BIOLOGY APPROACH 19
2.2.2 AURORA A KINASE LABELLING EXPERIMENTS WITH A REACTIVE FRAGMENT LIBRARY: FIXED TIME POINT, HIT VALIDATION AND TIME COURSE EXPERIMENTS. 20
2.2.3 IDENTIFICATION OF SITE OF LABELLING ON AURORA A KINASE 22
2.2.4 X-RAY CRYSTALLOGRAPHY OF AURORA A WITH FRAGMENT F104 23
3. COMPUTATIONAL METHODS 25
3.1 KNIME WORKFLOW FOR THE IDENTIFICATION OF DIRECT-TO-BIOLOGY SUBSTRATES 25
3.2 GENERATION AND ENUMERATION OF PRODUCTS OF DIRECT-TO-BIOLOGY REACTION ARRAYS 27
3.3 AUTOMATED ANALYSIS OF HIGH-THROUGHPUT SCREENING DATA TO DISCOVERY CHEMICAL MODIFIERS 34
3.4 MSCONVERT AND UNIDEC PARAMETERS FOR THE DECONVOLUTION OF PROTEIN MASS SPECTRA 36
3.5 COVALENT DOCKING OF D2B HIT FRAGMENTS ON AURORA A 38
3.6 FRAGPIPE WORKFLOW FOR AURORA A KINASE PEPTIDE MAPPING 40
4. CHEMISTRY EXPERIMENTAL 42
4.1 FRAGMENT STABILITY AND REACTIVITY ASSAYS 43
4.2 GENERAL PROCEDURE B – RE-SYNTHESIS OF DIRECT-TO-BIOLOGY HIT COMPOUNDS 44
4.3 COMPOUND SYNTHESIS 45
4.4 NMR DATA 51
5 REFERENCES 72

1. Supplementary Figures

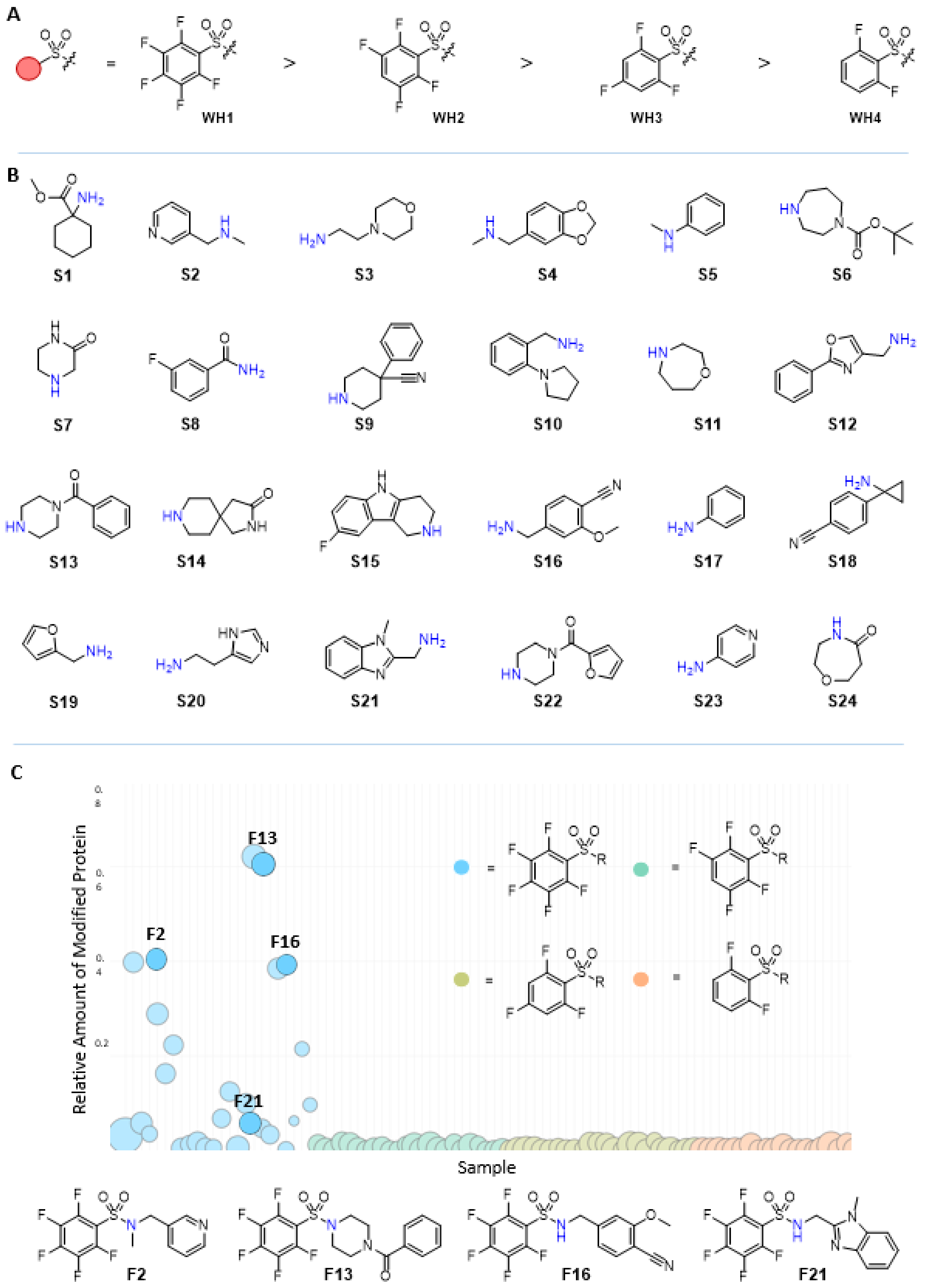

Figure S1: Preliminary direct-to-biology study. Panel A) Commercially-available fluorinated benzenesulfonyl chlorides used as warhead-containing building blocks. Panel B) Commercially-available amine building blocks. Panel C) Reactive fragments were prepared by connecting amine- and warhead-containing building blocks. Results from incubation of crude reaction mixtures (20 µM reactive fragment, assuming full conversion) with Aurora A kinase (2 µM) for 24 hr at room temperature.

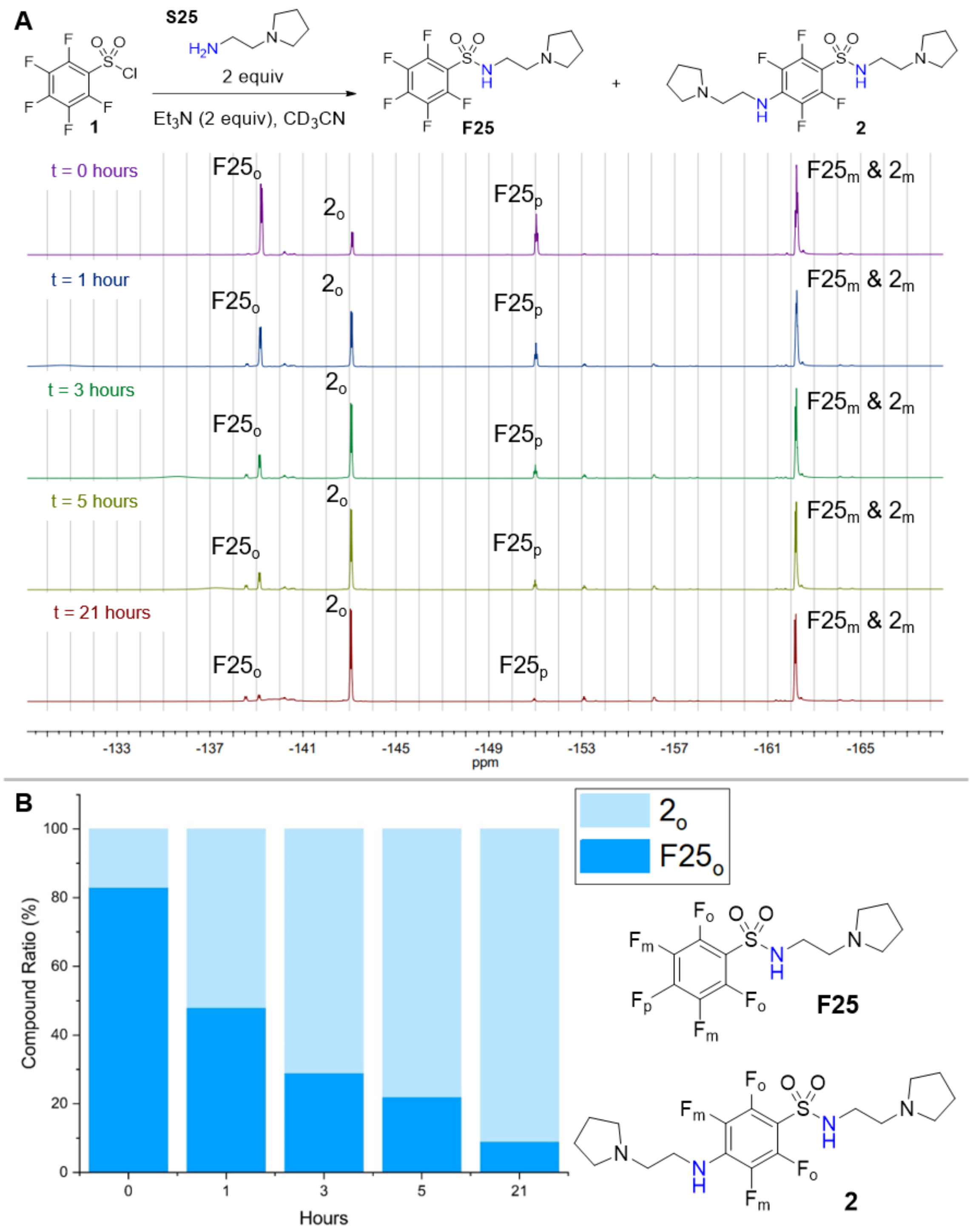

Figure S2: Optimisation of plate-based chemistry. Time-dependent formation of product **F25** and by-product **2** determined by ^{19}F NMR spectroscopy. NMR spectra (Panel A) and product distributions (Panel B) are shown.

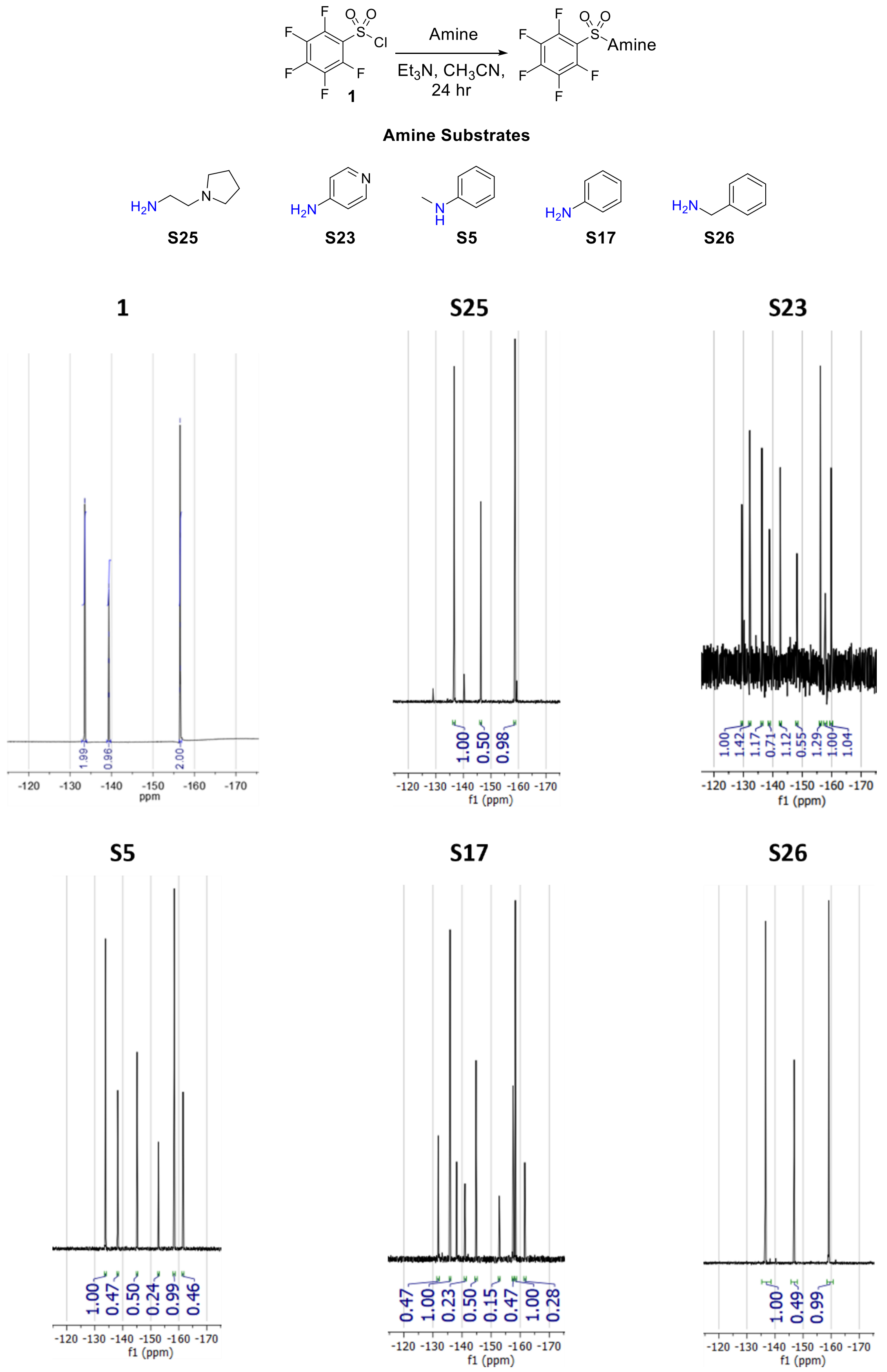

Figure S3: Determination of substrate scope of plate-based chemistry. ^{19}F NMR spectra of crude reaction mixtures obtained after 24 hr reaction under the specified conditions are shown.

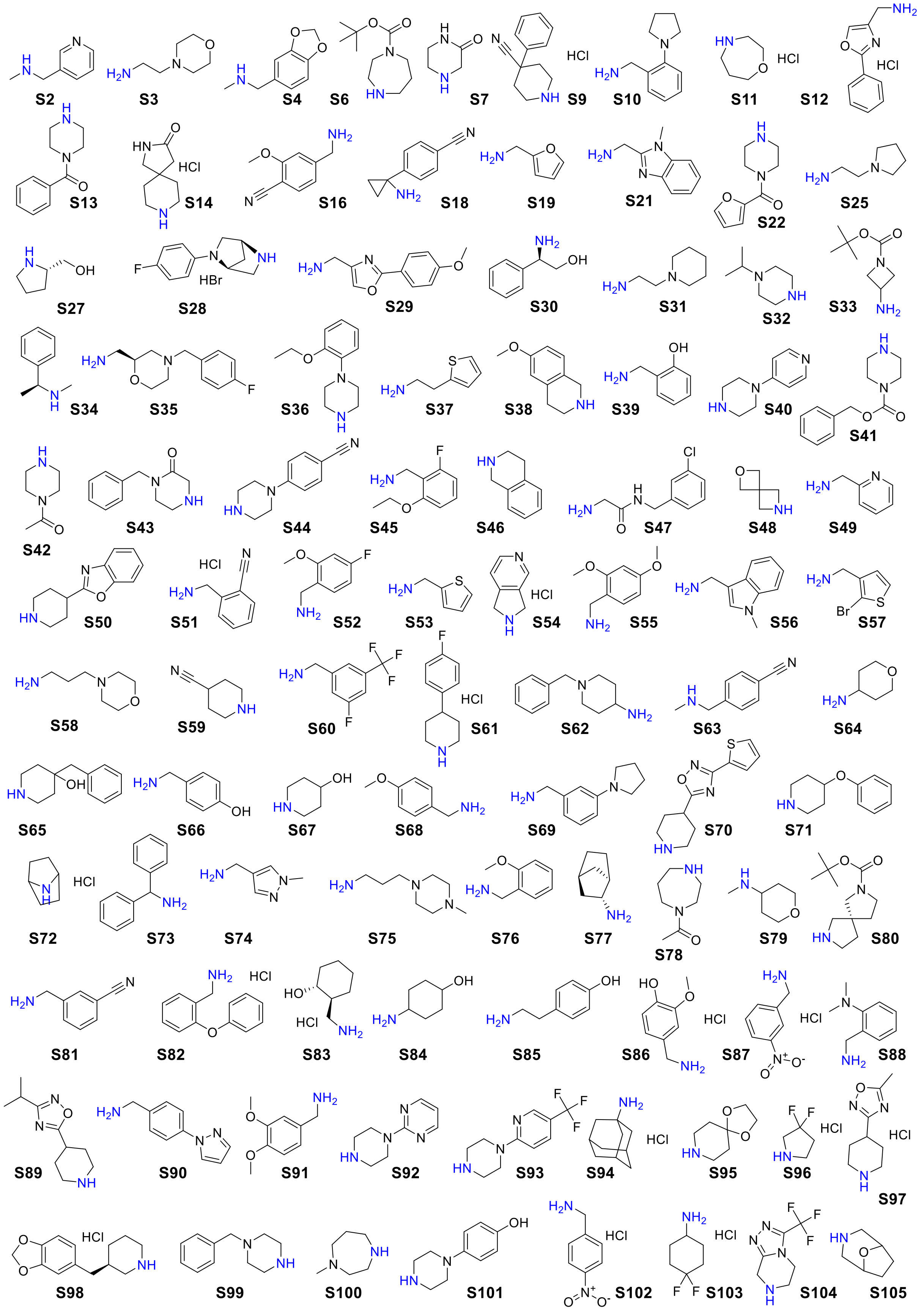

Figure S4: Substrates selected for the high-throughput synthesis of reactive fragments in 96-well plates. Substrates **S25-S105** were not used in preliminary experiments (see Figure S1).

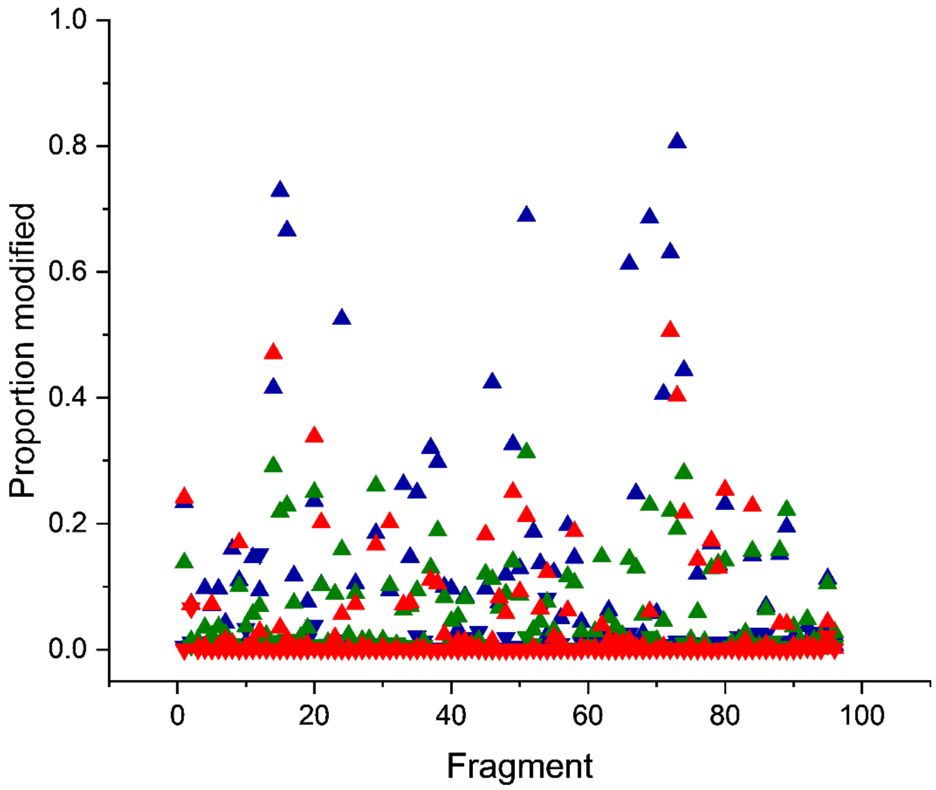

Figure S5: Screen of pentafluorobenzene sulfonamides against Aurora A (fragment concentration: 20 µM; blue), NEK7 (20 µM; green) and UbcH5B (200 µM; red). The proportion of protein (unmodified, or modified once, twice or three times) that has been modified once (up triangle) and twice (down triangle) is indicated by shape.

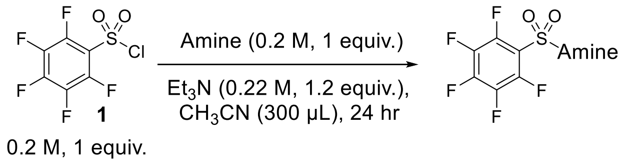

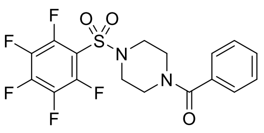

F13
Z = 3.0
Yield = 13%

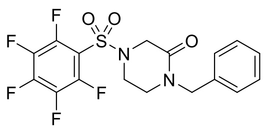

F43
Z = 2.7
Yield = 67%

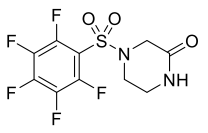

F7
Z = 2.8
Yield = 58%

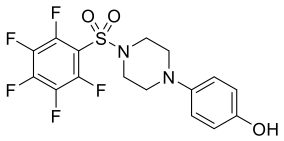

F101
Z = 2.6
Yield = 6%

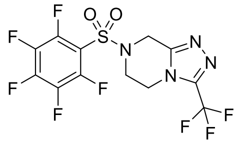

F104
Z = 2.8
Yield = 66%

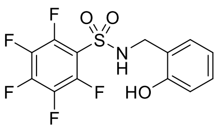

F39
Z = 3.5
not islated

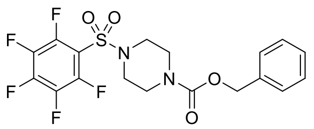

F41
Z = –0.7
Yield = 43%

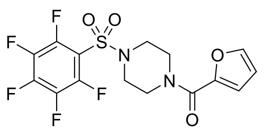

F22
Z = 0
Yield = 76%

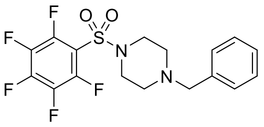

F99
Z = 1.3
Yield = 61%

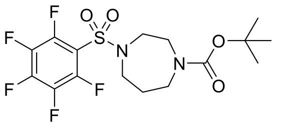

F6
Z = –0.2
Yield = >99%

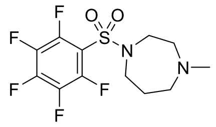

F100
Z = 2.5
Yield = 40%

Figure S6: Resynthesis of hit compounds and selected non-hit compounds. Calculated Z-scores from the original high-throughput screen are shown, together with yields of products (if isolated).

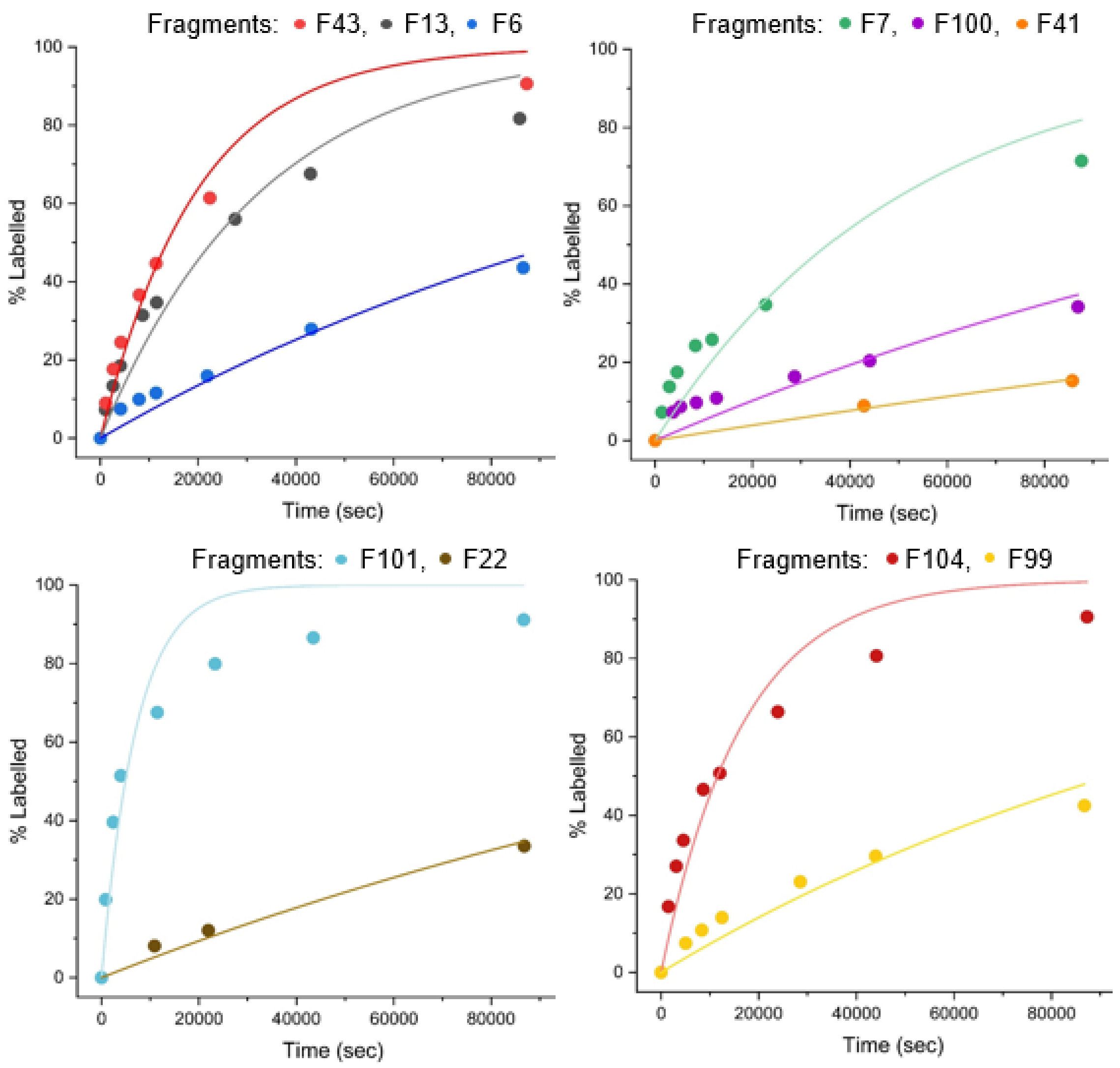

Figure S7: Time course of protein modification reactions involving purified hits determined by intact mass spectrometry. Reactive fragments (20 µM) were incubated with Aurora A kinase (2 µM) at 21 °C.

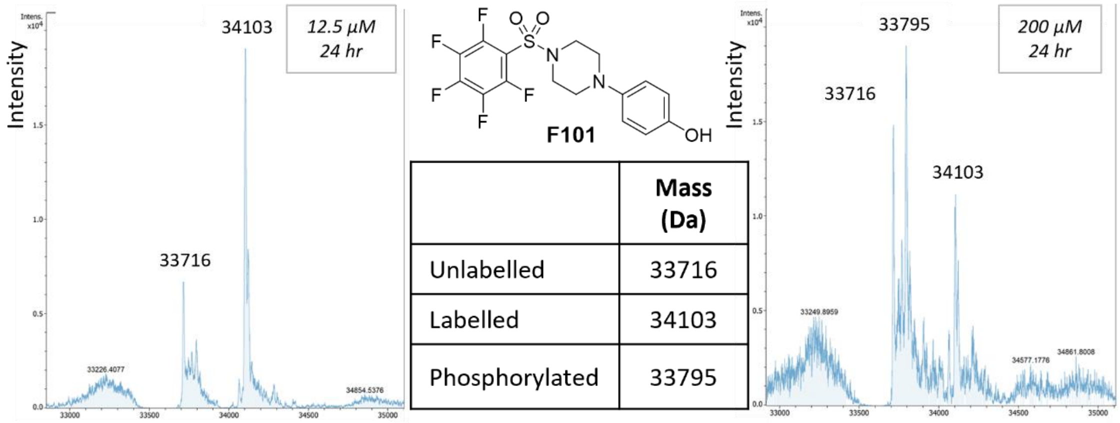

	Mass (Da)
Unlabelled	33716
Labelled	34103
Phosphorylated	33795

Figure S8: Exemplar deconvoluted intact MS data for modification of Aurora A kinase (2 µM) by fragment **F101** after 24 hr at 21 °C. Considerable phosphorylation of the unlabelled kinase (peak at 33795 Da) was observed with 200 µM fragment.

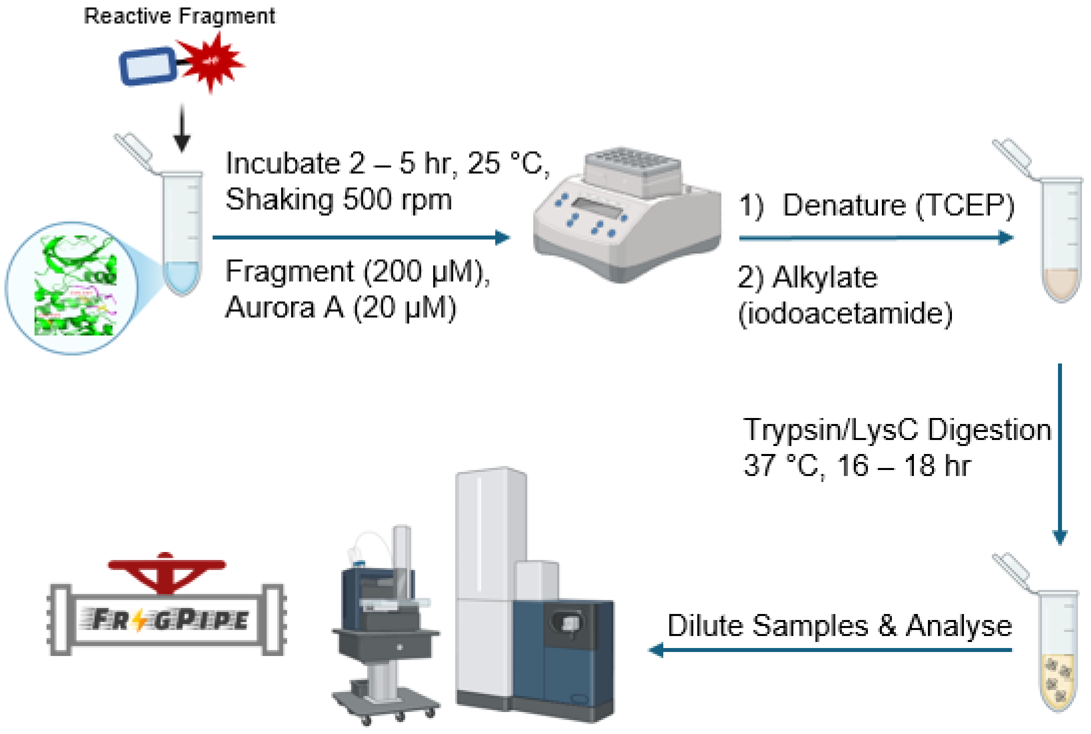

Figure S9: General workflow for the identification of fragment-labelled residues in Aurora A kinase.

A

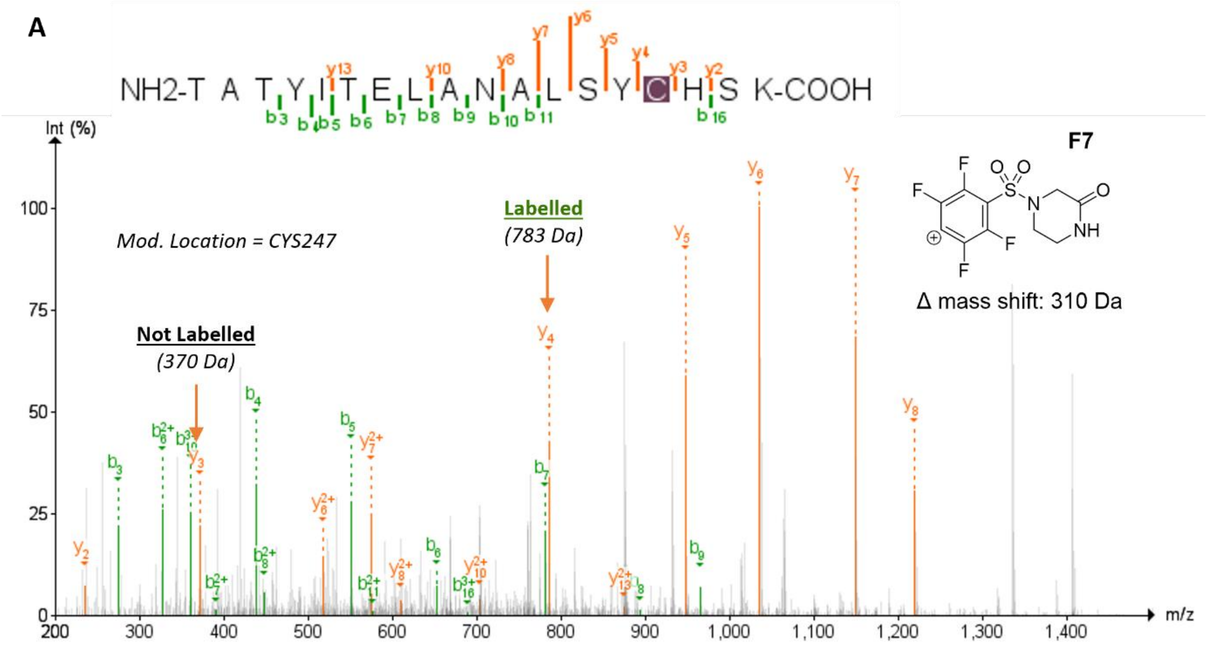

y_3: HSK-COOH *observed mass*: 370; *theoretical mass*: 370 no mass shift
y_4: CHSK-COOH *observed mass*: 783; *theoretical mass*: 473 mass shift of **310** Da

B

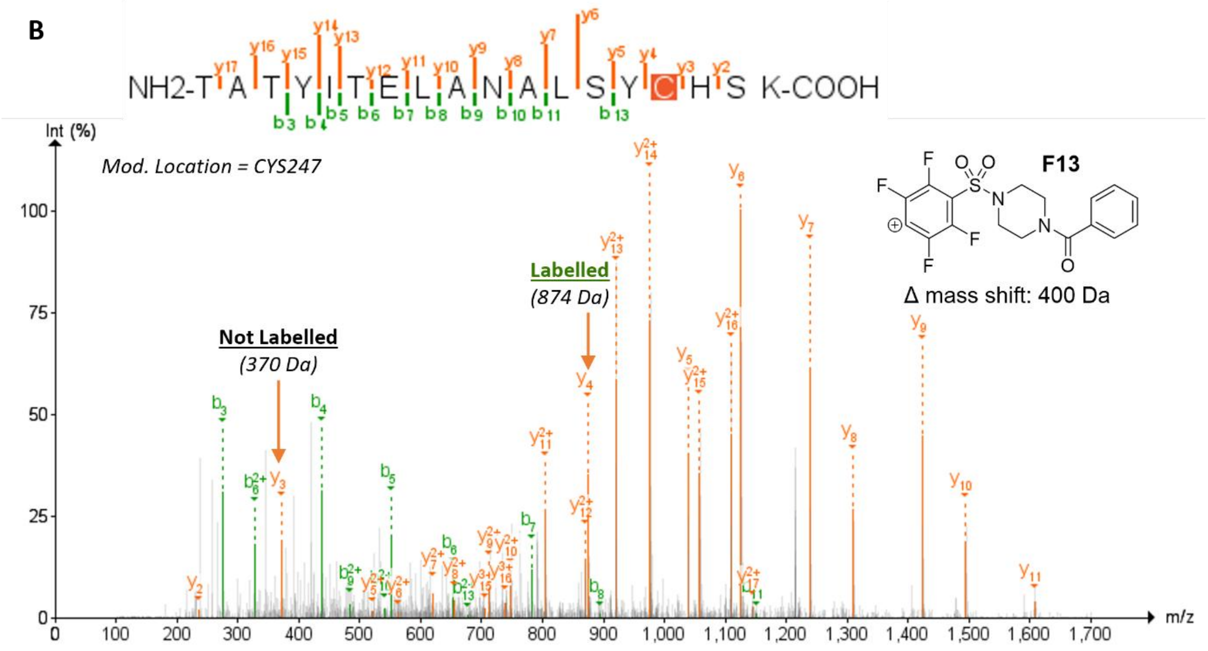

y_3: HSK-COOH *observed mass*: 370; *theoretical mass*: 370 no mass shift
y_4: CHSK-COOH *observed mass*: 873; *theoretical mass*: 473 mass shift of **400** Da

C

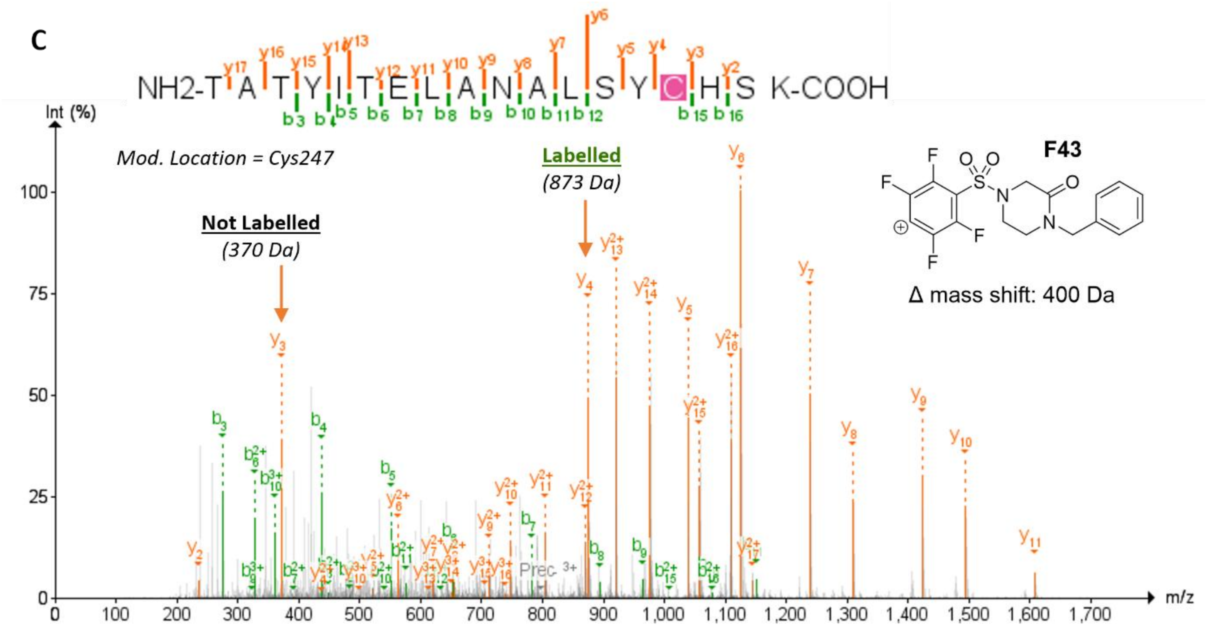

y_3: HSK-COOH *observed mass*: 370; *theoretical mass*: 370 <u>no mass shift</u>

y_4: CHSK-COOH *observed mass*: 873; *theoretical mass*: 473 <u>mass shift of **400** Da</u>

D

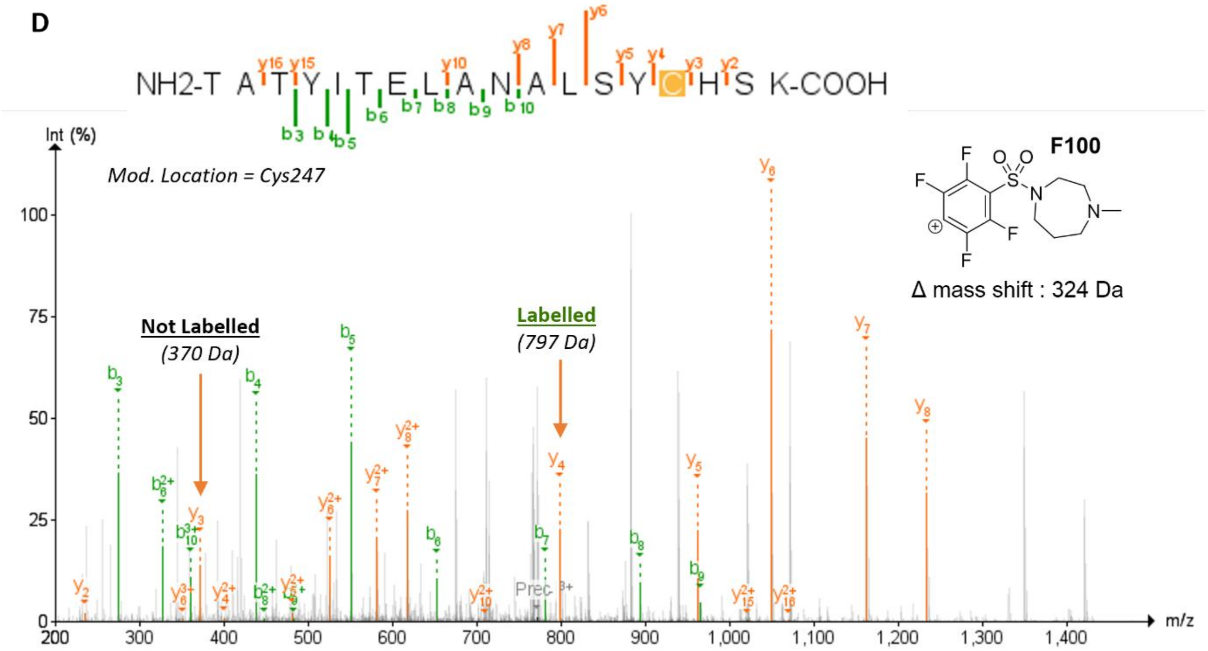

y_3: HSK-COOH *observed mass*: 370; *theoretical mass*: 370 <u>no mass shift</u>

y_4: CHSK-COOH *observed mass*: 797; *theoretical mass*: 473 <u>mass shift of **324** Da</u>

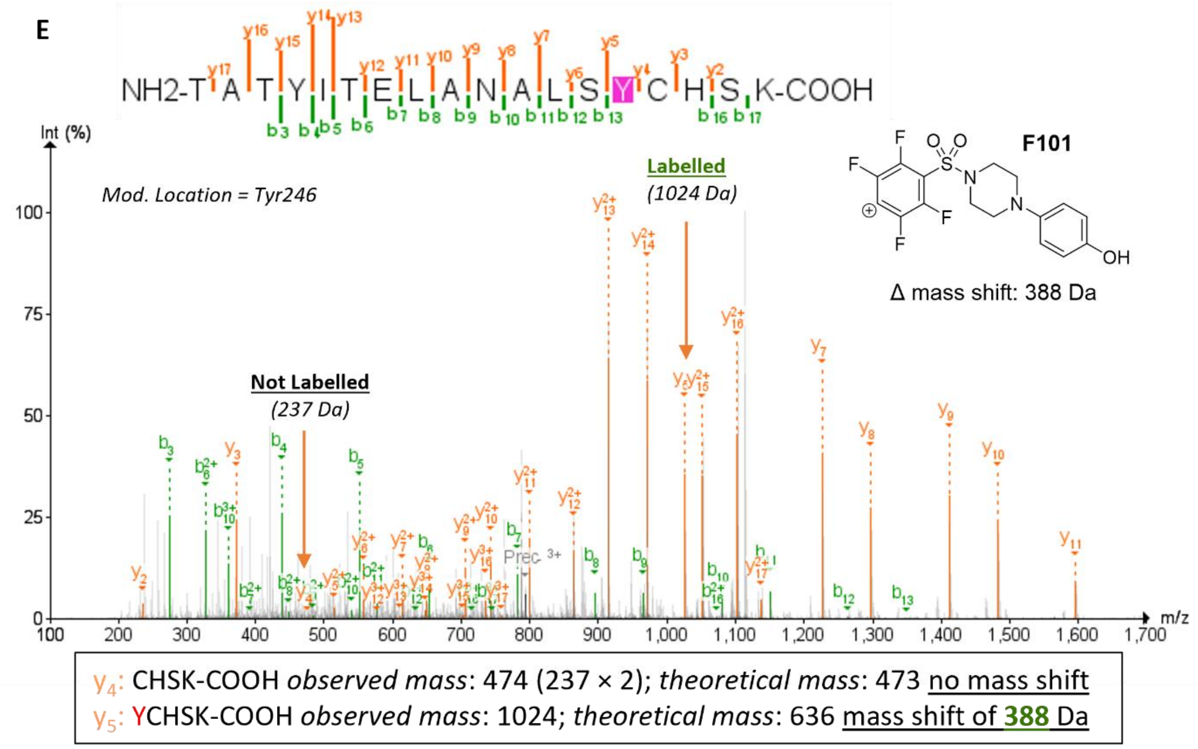

Figure S10: Exemplar mass spectrometry data processed on Fragpipe for the most abundant peptides modified with specific reactive fragments. Panel A) MS2 data showing modification of cysteine 247 by fragment **F7**; Panel B) MS2 data showing modification of cysteine 247 by fragment **F13**; C) MS2 data showing modification of cysteine 247 by fragment **F43**; D) MS2 data showing modification of cysteine 247 by fragment **F100**; E) MS2 data showing modification of tyrosine 246 by fragment **F101**.

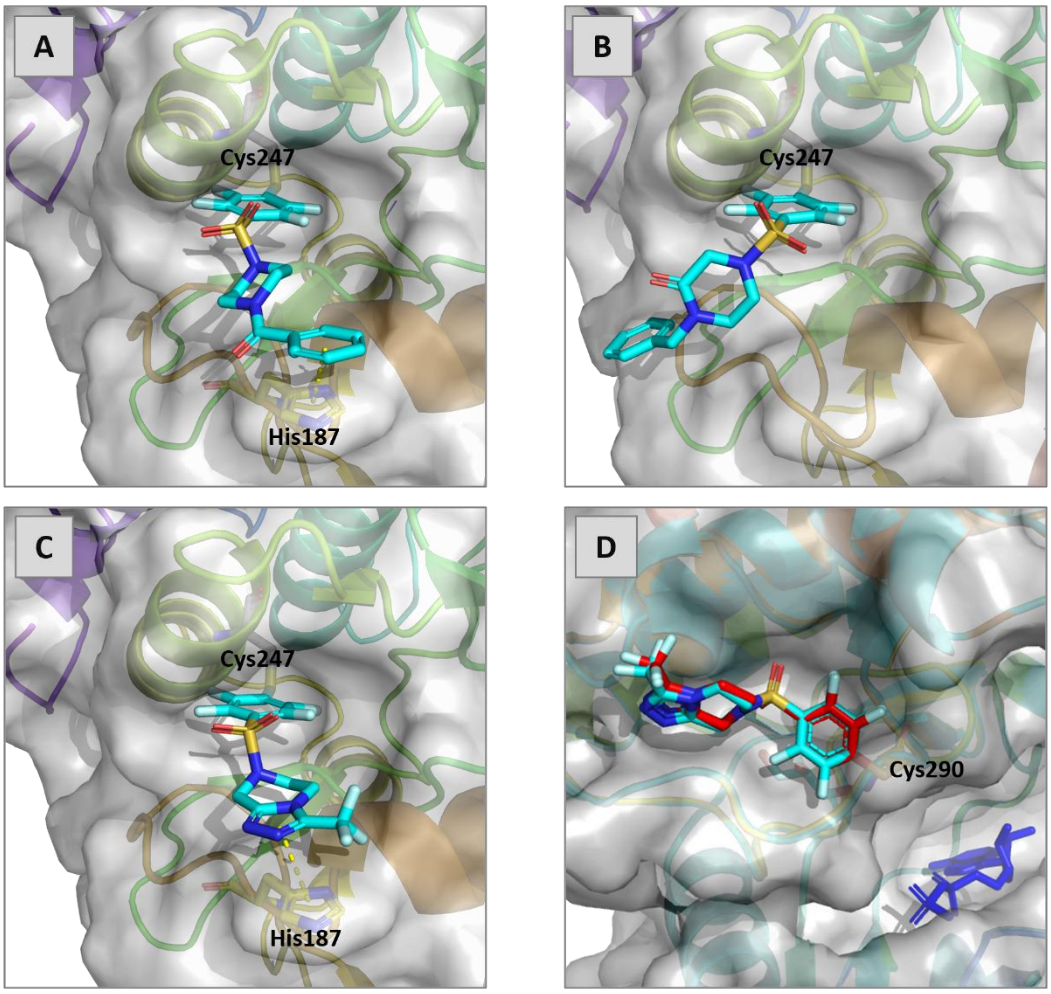

Figure S11: Structures of Aurora A kinase following covalent docking of selected reactive fragments to Cys247 [**F7** (Panel A), **F101** (Panel B) and **F100** (Panel C)] and to Cys290 [Panel D; **F104** (cyan) overlayed with crystal structure (red, PDB: 9SUY)].

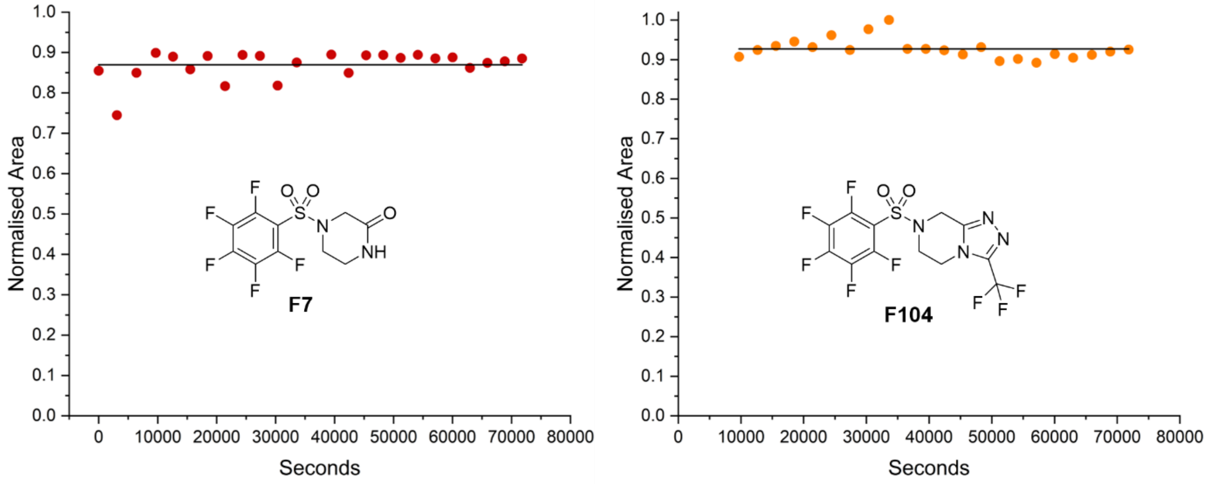

Figure S12: Stability assays for compounds **F7** (left) and **F104** (right), samples were left to incubate for 24 hr at 200 µM in TRIS buffer (25 mM TRIS, 150 mM NaCl, 5 mM $MgCl_2$) and injected into a HPLC system every 50 minutes. Left Panel: stability of fragment **F7** over 24 hr, showing no decomposition. Right Panel: fragment **F104** over 24 hours showing no decomposition

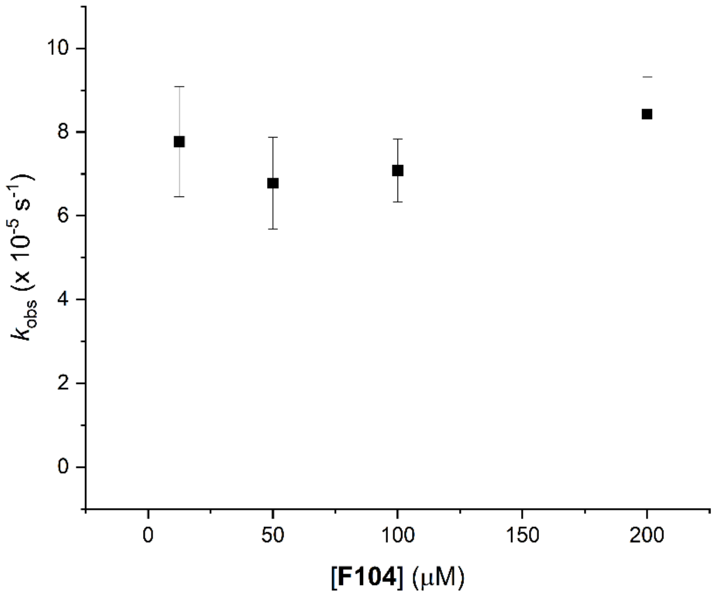

Figure S13: Observed rates of protein labelling by **F104** determined from experiments performed in duplicate. The bars represent standard fitting errors.

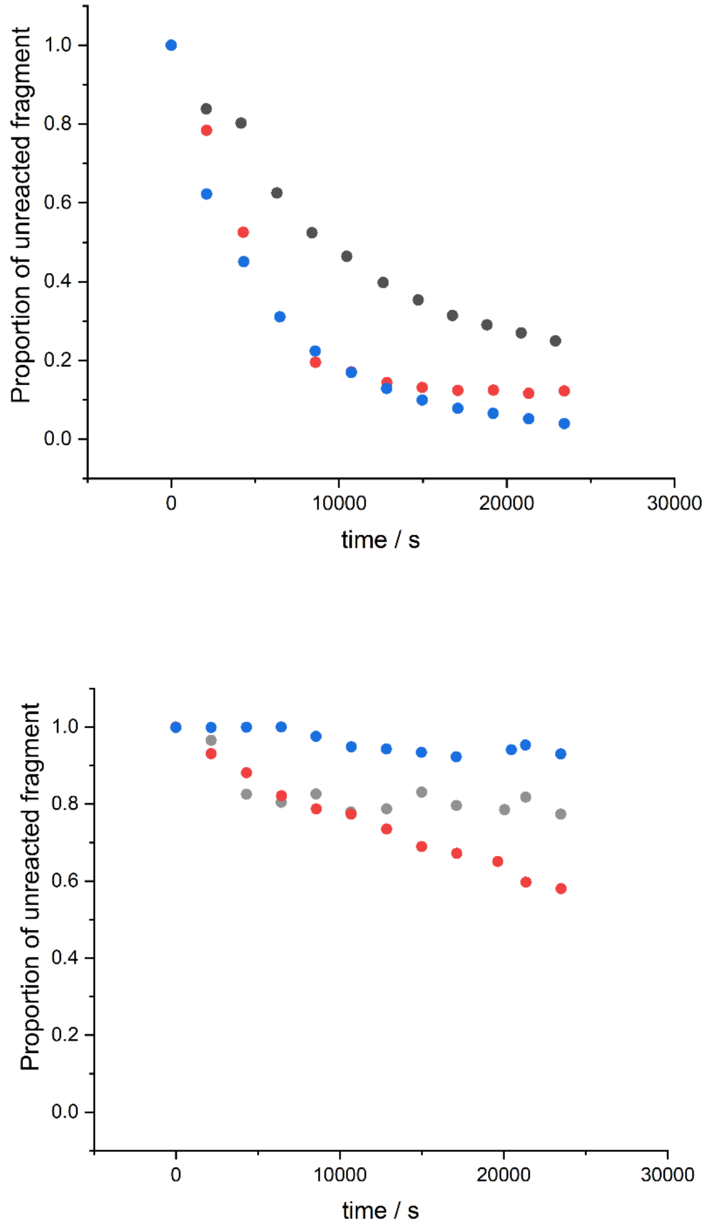

Figure S14: Reactivity of exemplar fragments. Panel A: Reactivity with 1 mM *N*-acetyl cysteine methyl ester at pH 7.5 (top): **F6** (black), **F100** (blue) and **F101** (red). Panel B: Reactivity with 1 mM *N*-acetyl lysine methyl ester at pH 10.7 (top): **F100** (blue), **F101** (red) and **F104** (grey).

2. Biology Experimental

2.1 Protein Sequences

<u>Aurora A Kinase [116-389] (MW: 33716 Da)</u>

GAMSYSYDAPSDFINFSSKQKNEESKKRQWALEDFEIGRPLGKGKFGNVYLAREKQSKFILALKVLFKAQLEKAGVE
HQLRREVEIQSHLRHPNILRLYGYFHDATRVYLILEYAPLGTVYRELQKLSKFDEQRTATYITELANALSYCHSKRVIHR
DIKPENLLLGSAGELKIADFGWSVHAPSSRRTTLCGTLDYLPPEMIEGRMHDEKVDLWSLGVLCYEFLVGKPPFEAN
TYQETYKRISRVEFTFPDFVTEGARDLISRLLKHNPSQRPMLREVLEHPWITANSSK

<u>Aurora A Kinase D274N variant for cystallography</u>

GAMSYSYDAPSDFINFSSKQKNEESKKRQWALEDFEIGRPLGKGKFGNVYLAREKQSKFILALKVLFKAQLEKAGVE
HQLRREVEIQSHLRHPNILRLYGYFHDATRVYLILEYAPLGTVYRELQKLSKFDEQRTATYITELANALSYCHSKRVIHR
DIKPENLLLGSAGELKIA**N**FGWSVHAPSSRRTTLCGTLDYLPPEMIEGRMHDEKVDLWSLGVLCYEFLVGKPPFEAN
TYQETYKRISRVEFTFPDFVTEGARDLISRLLKHNPSQRPMLREVLEHPWITANSSK

<u>NEK7 (MW: 35616 Da)</u>

MDEQSQGMQGPPVPQFQPQKALRPDMGYNTLANFRIEKKIGRGQFSEVYRAACLLDGVPVALKKVQIFDLMDA
KARADCIKEIDLLKQLNHPNVIKYYASFIEDNELNIVLELADAGDLSRMIKHFKKQKRLIPERTVWKYFVQLCSALEH
MHSRRVMHRDIKPANVFITATGVVKLGDLGLGRFFSSKTTAAHSLVGTPYYMSPERIHENGYNFKSDIWSLGCLLY
EMAALQSPFYGDKMNLYSLCKKIEQCDYPPLPSDHYSEELRQLVNMCINPDPEKRPDVTYVYDVAKRMHACTASS
LEHHHHHH

<u>UbcH5B (MW: 17167 Da):</u>

GPLGSMALKRIHKELNDLARDPPAQSRAGPVGDDMFHWQATIMGPNDSPYQGGVFFLTIHFPTDYPFKPPKVAF
TTRIYHPNINSNGSICLDILRSQWSPALTISKVLLSISSLLSDPNPDDPLVPEIARIYKTDREKYNRIAREWTQKYAM

2.1.1 Protein Expression

Human Aurora A kinase domain 116-389 fused at the N-terminus to TPX2 7-20 and mutants in vector (pET28a+) were transformed into RIL cells alongside the pCDF vector encoding lambda phosphatase. The plasmid was purchased from GenScript. The protein was overexpressed in LB, with growth at 37 °C until the O.D. at 600 nm reached 0.6-0.8. Expression was then induced with 0.5 mM IPTG overnight at 20 °C. The pelleted cells were resuspended in 10 ml of ice-cold lysis buffer per litre of grow (50 mM TRIS pH 7.5, 250 mM NaCl, 20 mM imidazole, 10% glycerol, 5 mM MgCl2, one EDTA-free protease inhibitor tablet per 50 ml of buffer). The resuspended cells were sonicated at 60% amplitude for 10 sec on, 20 sec off, 5 min total to lyse them. The soluble was collected at 17000 rpm for 5 min. After filtering through a 0.45 µm filter the soluble was loaded onto a HisTrap HP. Any bound protein was eluted free in a gradient of lysis buffer including 500 mM imidazole. The His-tag was then cleaved overnight using TEV protease in dialysis at 4 °C into 50 mM TRIS pH 7.5, 250 mM NaCl, 10% glycerol, 5 mM MgCl2. After dialysis the cleaved protein was rebound to the HisTrap equilibrated in dialysis buffer. The Aurora interacted with the HisTrap still after cleavage so a gradient of 500 mM imidazole was used to elute off the tag-free protein. The Aurora A containing fractions were concentrated down in a 10 kDa cut-off concentrator and loaded onto a SD200 16/600 size exclusion column equilibrated into 50 mM TRIS pH 7.5, 200 mM NaCl, 10% glycerol, 5 mM MgCl2. In the final step Aurora A was concentrated down again and flash-frozen before storage at -80 °C.

Full-length (FL) Nek7 bearing a non-cleavable C-terminal 6×His tag (cloned in pET30; Novagen) was co-transformed with λ-phosphatase (in pCDF-Duet) into *Escherichia coli* BL21(DE3)-RIL competent cells (Thermo Fisher Scientific). Cultures were grown in Luria–Bertani (LB) medium supplemented with kanamycin (50 µg ml^{-1}), spectinomycin (50 µg ml^{-1}), and chloramphenicol (25 µg ml^{-1}). Co-expression with λ-phosphatase was employed to prevent autophosphorylation and to obtain homogeneous, unphosphorylated Nek7 protein. Cell pellets were harvested and lysed by sonication in lysis buffer containing 50 mM HEPES (pH 7.5), 300 mM NaCl, 20 mM imidazole, 5 % (v/v) glycerol, 1 mM $MgCl_2$, 0.2 mM $MnCl_2$ and a protease inhibitor tablet. The clarified lysate was loaded onto a 5 ml HisTrap affinity column (Cytiva), pre-equilibrated with lysis buffer, and eluted using a linear gradient of 20–250 mM imidazole. Eluted fractions containing Nek7 were pooled, concentrated and subjected to size-exclusion chromatography on a Superdex 200 16/600 column (Cytiva) equilibrated with buffer containing 50 mM HEPES (pH 7.5), 300 mM NaCl, 5 % (v/v) glycerol and 5 mM dithiothreitol (DTT). Fractions corresponding to monomeric Nek7 were collected, concentrated and analysed by SDS–PAGE to assess purity and flash frozen in liquid nitrogen.

An expression construct for human FL UbcH5B$^{3C/S,S22R}$ cloned into pGEX-6P-3 was a gift from Prof. Catherine L. Day (Department of Biochemistry, University of Otago, Dunedin, New Zealand). Mutations of Cys21, Cys107, and Cys111 to Ser (3C/S) as well as of Ser22 to Arg (S22R) were introduced to reduce protein aggregation and prevent ubiquitin binding to the "backside" β-sheet of UbcH5B, respectively. UbcH5B$^{3C/S,S22R}$ was expressed with an N-terminal Glutathione S-transferase (GST) tag in *E. coli* BL21(DE3)-RIL cells. Cells were grown at 37°C in Terrific Broth (TB; Fisher Chemical) media supplemented with ampicillin (100 µg ml^{-1}) and chloramphenicol (34 µg ml^{-1}) to an OD_{600} of ~ 1.4, then induced with 0.5 mM isopropyl-β-D-1-thiogalactopyranoside (IPTG; Fluorochem) and grown overnight (O/N) at 18°C. Purification of UbcH5B$^{3C/S,S22R}$ was carried out as previously described,[1-3] with minor modifications. Briefly, cells were resuspended in 100 ml ice-cold 1x phosphate-buffered saline (1x PBS) supplemented with 0.075% (v/v) β-mercaptoethanol, 1 mM benzamidine, 0.8 mM phenylmethylsulfonyl fluoride (PMSF), and 0.3 mg/ml lysozyme. Cells were lysed by sonication using a Sonics Vibracell instrument (1 s ON/3 s OFF, at 40% amplitude for 4 min), and the lysate was cleared by centrifugation at 30,000 g for 30 min at 4°C. The clarified lysate was subsequently passed through a 0.45 mm filter (Fisher Scientific), and then incubated with 2 ml Glutathione Sepharose™ 4 Fast Flow resin (Cytiva), pre-equilibrated in 1x PBS, for 2 h at 4°C in rotation. After washing the resin four times with 40 ml 1x PBS, the resin-bound UbcH5B$^{3C/S,S22R}$ was resuspended in 2 ml 1x PBS and incubated with 0.3 mg GST-HRV 3C protease and 5 mM DTT O/N at 4°C in rotation. After O/N incubation, the supernatant containing cleaved UbcH5B$^{3C/S,S22R}$ was concentrated and further purified on a HiLoad Superdex 75 16/600 column (GE Healthcare) pre-equilibrated in 1x PBS and 1 mM tris(2-carboxyethyl)phosphine (TCEP). Eluted peaks were analysed by SDS-PAGE, and fractions containing >95% pure UbcH5B$^{3C/S,S22R}$ were combined, concentrated to 3 mg/ml, snap-frozen in liquid nitrogen, and stored at -80°C.

2.2 Protein Labelling Experiments

2.2.1 Discovery of Reactive Fragment Hits using a Direct-to-Biology Approach

High-throughput Direct-to-Biology reaction arrays were carried out in a PTFE custom-made 96-well plate (8 × 12) with inserts for borosilicate glass vials (vial volume = 750 µL, vial dimensions = 8 × 30 mm, CV-2100-0830 Chemglass). Stock solutions of triethylamine (66 mM in MeCN), perfluorobenzenesulfonyl chloride (60 mM in MeCN) and corresponding amines (60 mM in MeCN)

were prepared. Stock solutions of amines that were salts were dissolved in a solution of triethyl amine (60 mM in MeCN). A magnetic follower was added to each vial followed by a solution of triethylamine (30 µL, 66 mM in MeCN). A solution of the corresponding amine substrate was then added (30 µL, 60 mM in MeCN) followed by a solution of perfluorobenzenesulfonyl chloride (30 µL, 60 mM in MeCN). All reagents were transferred to glass vials using a multichannel pipette. The final concentration of reagents in the plate was as follows: triethylamine (22 mM, 1.2 equiv); amine substrate (20 mM, 1 equiv); perfluorobenzenesulfonyl chloride (20 mM, 1 equiv) in a total reaction volume of 90 µL of MeCN. The glass vials were then capped and the reactions were allowed to stir at room temperature for 24 hr. The solvent was then removed under reduced pressure in a desiccator. The remaining crude residues were dissolved in 90 µL of DMSO so that the final concentration of product was 20 mM (assumed full conversion of starting material to product). Crude DMSO solutions were then stored at –20 °C prior to screening.

Crude reaction mixtures in DMSO (20 mM) were diluted tenfold in DMSO in a separate PTFE custom made 96-well plate (8 × 12) with inserts for borosilicate glass vials (vial volume = 750 µL, vial dimensions = 8 × 30 mm, CV-2100-0830 Chemglass). The resulting solutions (2 mM) were then diluted a further tenfold in freshly made TRIS buffer in a 384-well Perkin Elmer plate to make the desired working solutions (200 µM, 10% DMSO). Working solutions of crude reaction mixtures were then added to a Corning 384-well plate (part no. 4514) containing Aurora A kinase in TRIS buffer (from a 110 µM stock solution). The final volume of the incubation mixture was 30 µL with protein (2 µM, 1 equiv.) and reactive fragment (20 µM, 1% DMSO, 10 equiv.), assuming complete conversion of substrates into products. The plate was then sealed with adhesive foil and the mixtures were allowed to stand for 24 hr at room temperature (circa 20 °C). Each reaction was then injected (3 µL) onto a high-resolution mass spectrometer. Maximum entropy deconvolution methods were used as part of the downstream processing of acquired spectra to determine the quantity of unlabelled and labelled protein masses. A bespoke Python script was utilised to rapidly extract, process and visualise MS data acquired from protein labelling reactions (see Section 3.3 for details).

2.2.2 Aurora A Kinase Labelling Experiments with a Reactive Fragment Library: Fixed Time point, Hit Validation and Time Course Experiments.

Solutions of purified compounds in DMSO (20 mM) were diluted tenfold in DMSO in Eppendorf tubes. Stock solutions (2 mM) were then diluted a further tenfold in TRIS buffer to make the desired working solutions (200 µM, 10% DMSO). Working solutions of reactive fragments were added (10 µL) to Waters QuantRecovery MaxPeak vials (part number: 186009186) containing a solution of Aurora A kinase in TRIS buffer. The final volume of the labelling reactions was 100 µL with kinase (2 µM, 1 equiv.) and reactive fragment (20 µM, 1% DMSO, 10 equiv.). Sealed vials were incubated on a high-resolution mass spectrometer autosampler at 21 °C. Aliquots of the samples (5 µL) were then injected at 0 min, 30 min, 1 hr, 2 hr, 3 hr, 6 hr, 12 hr and 24 hr time points. Exact time points in seconds were then calculated from collected data. For kinetics experiments, concentrations of reactive fragments, investigated in duplicate, were varied (12.5 µM, 50 µM, 100 µM and 200 µM). The model shown in Figure S15 was then used to calculate kinetic parameters.

Kinetics Equations

$$\mathrm{P} + \mathrm{L} \underset{}{\overset{\mathrm{K}_D}{\rightleftharpoons}} \mathrm{P}\cdot\mathrm{L} \xrightarrow{k_{\mathrm{mod}}} \mathrm{P{-}L}$$

1) $\mathrm{K}_D = \frac{[P][L]}{[P \cdot L]}$

2) $k_{\mathrm{obs}} = k_{\mathrm{mod}} \cdot \frac{[P \cdot L]}{[P_{umod}]}$

3) $P_{unmod} = [P] + [P \cdot L]$

4) $\%mod = 100 \cdot (1 - e^{-k_{obs}t})$

Figure S15: Model for modification of a protein, P, with a modifier, L. Parameters for protein modification reactions are defined.

The ExpAssoc1 equation in OriginPro was used to determine k_{obs} (= 1/τ) at different concentrations of reactive fragment:

$$y = Yb + A \times \left[1 - e^{-\frac{(x - TD)}{\tau}}\right]$$

The time offset *TD* and the baseline value *Yb* were both set to 0, on the basis that, at T_0, none of the protein was modified. The data presented in Figure 2E and Supplementary Figure S6 assumed labelling approached 100% (i.e. *A* = 100).

2.2.3 Identification of Site of Labelling on Aurora A Kinase

A control sample of Recombinant Aurora A kinase (18 mg/mL, 332 µM) domain [116-389] was prepared by transferring 2.4 µL of stock solution to a 1.5 mL low binding Eppendorf containing ammonium bicarbonate (ABC) buffer (37.6 µL). A solution of DMSO (4 µL, 1% in 50 mM ABC buffer) was then added to the control sample. A second sample was prepared by transferring 2.4 µL of kinase stock into ABC buffer (33.6 µL) followed by a probe stock solution (4µL, 2mM, 1% DMSO in ABC buffer). The final concentration of protein was 20 µM (c.a. 26 µg), DMSO 1% and probe 200 µM. The samples were incubated for 2 to 5 hr at 25 °C. An aliquot was taken of each sample (10 µL) and 3 µL injected onto a high-resolution mass spectrometer to verify sample integrity and extent of labelling.

Next, Waters RapiGest SF Surfactant (5.5 µL, 1% w/v in ABC buffer, Part Number: 186001861) was added to each protein sample followed by TCEP·HCl (5 µL, 55 mM in ABC buffer). Protein samples were then reduced and denatured at 60 °C for 30 min with agitation (500 rpm). Alkylation was carried out by transferring a solution of iodoacetamide (5 µL, 180 mM in ABC buffer) to each sample. Samples were then allowed to stand in the dark for 30 min at room temperature. Removal of small molecules and reagents was achieved with overnight acetone (240 µL) precipitation at -20 °C.

Proteins were then pelleted by centrifugation at 14k × g for 10 min at 4 °C, the supernatant was discarded and the pellets were washed with a 4:1 mixture of acetone/ABC buffer (300 µL). This process was repeated once more, and the protein pellets were allowed to air dry for a maximum of 30 min. Proteins were then resuspended in a RapiGest solution (18 µL, 0.11% in ABC buffer). Solubilisation was aided with sonication and incubation at 30 °C with agitation (1000 rpm). Proteins were then digested for 16 hr at 37 °C with agitation (500 rpm) with a working solution of Trypsin/LysC (2 µL, 0.1 µg/µL in ABC buffer, Promega, Product Code: V5073).

Samples were then spun at 10k × g for 1 minute and incubated for 15 min at 37 °C with agitation (500 rpm). Enzymatic digestion was then stopped with TFA (4 µL, 3% in H_2O). Samples were incubated for a further 45 min at 37 °C with agitation (500 rpm) and centrifuged at 13k × g for 10 min. Samples for LC-MS/MS analysis were prepared by adding 88 µL of a 70% MeCN solution with 0.1% FA to each digest, then 20 µL of this digest solution was diluted further in 530 µL of a 0.1% FA in H_2O solution. Samples were centrifuged at 10k x g for 2 min and supernatant (50 µL) was transferred into an appropriate vial for timsTOF LC-MS/MS analysis. See Section 3.6 for details of data analysis.

Compound	Mass Shift (Da)	Sequence Coverage	Identified by (ion)	Residue	% Intensity
**F7**	310	86%	y_4	Cys247	78
			b_5, y_{15}	Cys290	18
**F13**	400	99%	y_4	Cys247	81
			b_5, y_{15}	Cys290	18
**F43**	400	95%	y_4	Cys247	66
			b_5, y_{15}	Cys290	32
**F100**	324	86%	y_4	CYS247	87
			b_5, y_{15}	CYS290	9
**F101**	388	99%	y_5	Tyr246	49
			y_4	Cys247	38
			b_5, y_{15}	Cys290	11
**F104**	402	99%	y_4	Cys247	60
			b_5, y_{15}	Cys290	35

Table S1: Label-free quantification of modified peptides and diagnostic MS2 ions. Labelling events with <5% intensity are not listed.

2.2.4 X-Ray Crystallography of Aurora A with Fragment **F104**

Purified Aurora-A 116-389 D274N (SEE Section 2.1 for sequence) fused at the N-terminus to TPX2 7-20 was labelled with compound **F104** at 200 µM for 16 hour at 20 °C. The labelled Aurora-A was then concentrated down to 20 mg/ml in a 30 kDa cut off concentrator (Amicon). ADP was added to a final concentration of 5mM and the protein was left on ice for 30 minutes before it was screened against a range of commercial crystallisation matrices. Drops were laid down at a 1:1 ratio of Aurora-

A:precipitant in MRC sitting drop plates using a Mosquito LCP crystallisation robot (STP labtech) and incubated at 18 °C. Crystals were produced using Morpheus Fusion (Molecular Dimensions) condition E4 (0.1 M Carboxylic acids, Buffer system 1 pH 6.5, 1.2% Cholic acid derivative, 30% precipitant mix 3 (40% Glycerol, 20% PEG 4000)) as the precipitant after 4 days. Crystals were flash-frozen in liquid nitrogen. Diffraction data were collected from a single crystal at Diamond Light Source (Oxford, UK) on beamline I03. Autoprocessed data from the xia2 dials pipeline at Diamond Light Source were used for structure determination.[4,5] Molecular replacement was performed in PHASER[6] using the structure of Aurora-A fused with TPX2 and bound to AMP-PNP as a model (PDB 6VPG).[7] Clear difference density was observed for the covalent modification of Cys290, and this was modelled in using Coot.[8] Subsequent rounds of iterative refinement were performed using Coot (data collection and refinement statistics can be found in Appendix Table S1). MolProbity was used to determine structure quality and PDB-Redo used to aid refinement.[9,10]

	TPX2-fused Aurora-A D274N modified with **F104**
Residue range	TPX2 7-20 fused to Aurora-A 116-389
**Data collection**	
X-ray source	DLS i03
Wavelength (Å)	0.9762
Resolution range (Å)	60.16-2.27 (2.27-2.31)
Space Group	P 41 21 2
Unit cell (a, b, c) (Å)	85.085, 85.085, 141.44
Unique reflections	23451 (1194)
Multiplicity	26.7 (27.7)
Mean I/σ(I)	8.4 (0.96)
Completeness	99.92 (98.6)
CC 1/2	1 (0.3)
**Refinement statistics**	
Resolution limits	2.27-2.39
Number of reflections	1665
R work	0.207
R free	0.251
Number of nonhydrogen atoms	2425
No. atoms Macromolecule	2223
No. atoms Ligand	83
No. atoms Water	119
RMSZ bond lengths (Å)	0.54
RMSZ angles (°)	1.02
Ramachandran favoured	97 %
Ramachandran outliers	0 %
MolProbity Clashscore	4
Average B-factor	64
PDB ID code	9SUY
Deposition code	D_1292151116

Table S2: Data collection and refinement statistics, values in parentheses are for highest-resolution shells

3. Computational Methods

3.1 KNIME Workflow for the Identification of Direct-to-Biology Substrates

KNIME version 4.7.7 with Chemistry Add-Ons was used to create a workflow for the identification and selection of structurally diverse libraries of amines that were available in our inventory of chemicals. The selected amines were then used as substrates for direct-to-biology experiments. The first six nodes (Figure S16) were connected sequentially with the following settings:

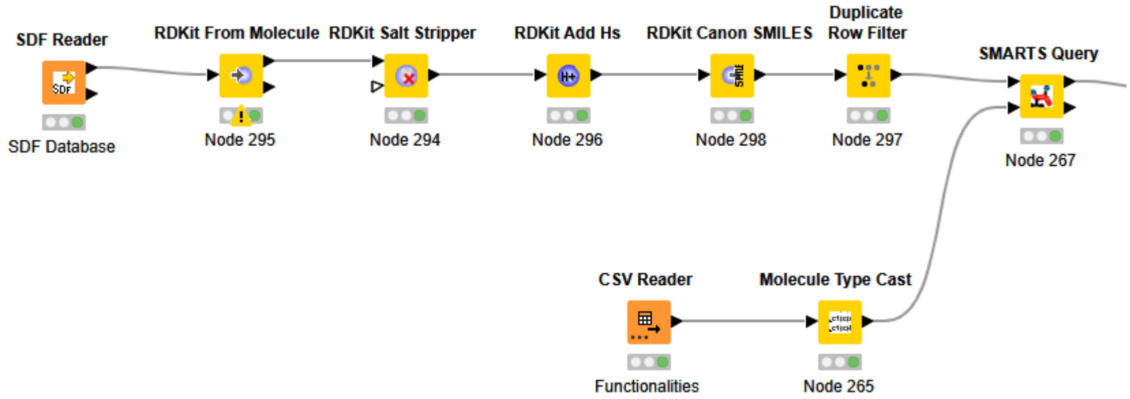

Figure S16: Showing the nodes used in the first section of the KNIME workflow, up to the SMARTS Query node.

- SDF Reader node was used to read in-house chemical inventories as .sdf files with *Extract SDF blocks* checked.
- RDKit from Molecule node with *Molecule column* set to *SDF Molecule* and *Error Handling* set to *Send erroneous rows to second output*. All other node settings remained unchanged.
- RDKit Salt Stripper node was added to the workflow, *RDKit Mol column* was configured to read *RDKit Mol* with *Keep only largest fragment after salt stripping* checked. All other settings remained unchanged.
- RDKit Add Hs was then connected to the RDKit Salt Stripper node and made to read the *salt stripped molecule* column, this was done by selecting *salt stripped molecule* in the dropdown menu under RDKIT Mol column. All other settings remained unchanged.
- RDKit Canon SMILES node was made to read the *Molecule (RDKit Mol)(Added Hs)* column by selecting the corresponding column from the dropdown menu under RDKit Mol Column, all other settings were left unchanged.

- Duplicates were then removed with the Duplicate Row Filter node. *Manual Selection* and *enforce exclusion* were checked, the green *include* box was made to contain *substance CAS* and *Canonical SMILES*.

The Duplicate Row Filter node was then connected to the first input port of a SMARTS Query node to select molecules by functional groups. The second input port was connected to a Molecule Type Cast node and a CSV Reader node (Figure S17). The following settings were used for the four nodes:

- The CSV reader node was made to read a .csv file containing the following SMARTS pattern *[NX3;H2,H1;!$(NC=O)]*. This allows the selection of compounds that contain amine functionalities and excludes compounds that contain only amides in their structure. The following settings were used: Under *Input Location > Read from > Local File System,* and *Mode > File* was checked. Under *Reader Options > Format > Autodetect format* was selected and the following symbols were used: *Column delimiter contained a , (comma)* with *Row delimiter* checked, *quote char* and *quote escape char* both contained *" (double quote), Comment char* in *Reader Options contained # (hash)*. *Has column header* was checked. All other settings remained unchanged.
- The Molecule Type Cast node was made to read *SMARTS* by using the dropdown menu under *Structure Column. Structure Type* was made to read *Smarts* by using the dropdown menu. The Molecule type cast node was then connected to the second input port of the SMARTS query node.
- The SMARTS query node was configured as follows: Under *Source > Molecule > Canonical SMILES* was selected from the dropdown menu and *SMARTS > SMARTS* was selected from the

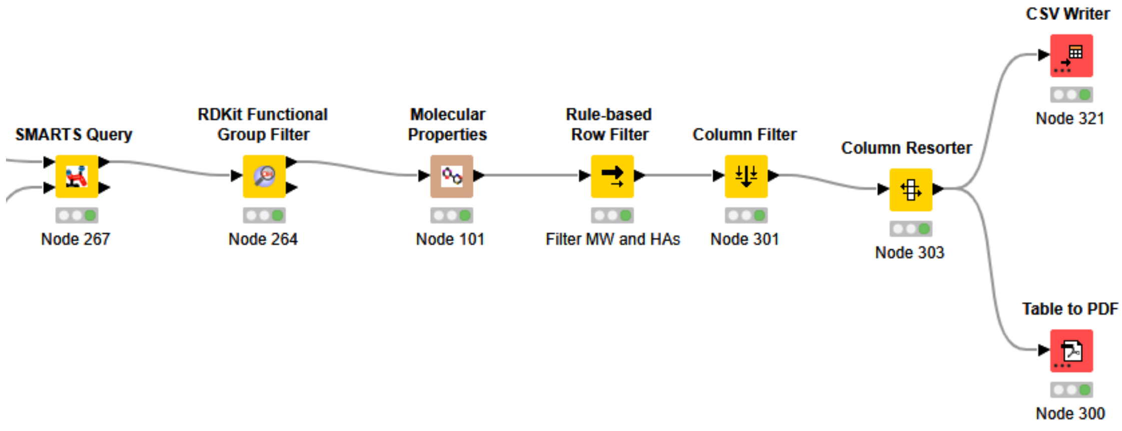

Figure S17: Showing the nodes used in the second and final section of the KNIME workflow from SMARTS Query to the final writer nodes.

dropdown menu. Under *parameters* both boxes were left unchecked. All other settings were left unchanged.

The SMARTS query node was then connected to a further sequence of five nodes with the following settings:

- Undesired functionalities were removed with the RDKit Functional Group filter (Figure S15) with the following settings: *Select molecule column > RDKit Mol Column > Salt Stripped Molecule*; A bespoke functional group definition file was used (see Table S3) and *Enable recording in the following new column: First Non-Matching Pattern* was checked.
- Molecules were then filtered by molecular properties using the Molecular Properties node. This was configured as follows: Under *Column Selection > Canonical SMILES* was selected from the dropdown menu*; Enforce exclusion* was checked under the red *Exclude* box. The green *Include* box contained the following molecular properties: *Aromatic Atoms Count, No. of Heavy Atoms, Rotatable Bonds Count, Rotatable Bonds Count (non terminal), XLogP*. All other settings remained unchanged.
- A Rule-based Row Filter was then connected to the workflow, in *Expression* the following rules were added*:*
  $Heavy Atoms Count$ ≤ 7 => TRUE
  $Heavy Atoms Count$ ≥ 16 => TRUE
  $XLogP$ ≥ 3.00 => TRUE
  $XLogP$ ≤ -2.0 => TRUE
  $Rotatable Bonds Count (non terminal)$ ≥ 3 => TRUE
  Exclude TRUE matches was checked.
- Four optional nodes were then added which included a Column Filter node to select which columns to include in the output data file. A Column Resorter node to reorder the columns as desired. Lastly, a CSV Writer node and a Table to PDF node were added to complete the workflow and save the generated list of amines. Amines were then manually selected from this list and used as substrates in the high-throughput synthesis and direct to biology procedure (Table S4).

3.2 Generation and Enumeration of Products of Direct-to-Biology Reaction Arrays

A separate KNIME workflow was created to computationally generate sulfonylation products from the selected amine library (Figure S18). The computationally generated compound data was then used for the downstream analysis of protein modification reactions. To achieve this two .sdf files were created with one containing the perfluorophenylsulfonyl chloride and the second containing the library of amines.

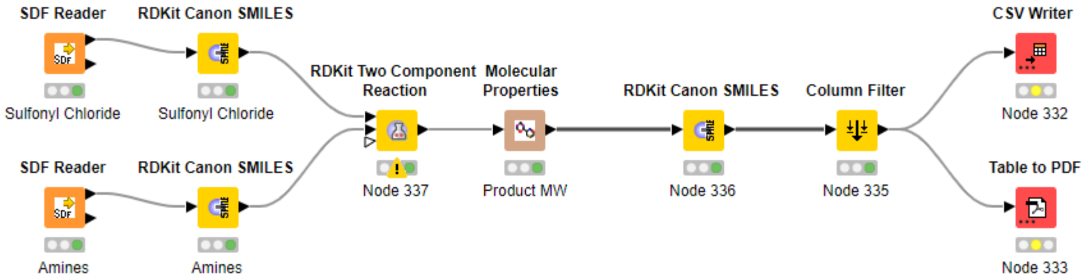

Figure S18: Showing the full KNIME workflow for the computational generation of sulfonylation reaction products with our library of selected amines.

- The workflow was started with two separate SDF Reader nodes (one for each .sdf file, see Figure S15) with *Extract SDF blocks* checked.
- Two separate RDKit Canon SMILES nodes were then connected to each SDF Reader mode to generate canonical SMILES of the molecular structures. The nodes were both configured as follows: *RDKit Mol column* was made to read *SDF Molecule* in the configuration settings.
- An RDKit Two Component Reaction node. This was configured as follows: Under *Reaction > Reactants 1 RDKit Mol column > SMI Chlorides* was selected from the dropdown menu*; Reaction > Reactants 2 RDKit Mol column > SMI* Amines was selected from the dropdown menu. In *Reaction SMARTS* the following string was used *[S:1][Cl].[N;!$(N[C,S]=[S,O,N]):2]>>[S:1][N;!$(N[C,S]=[S,O,N]):2]*. *Randomize reactants* was left unchecked and in *Other Options > Do matrix expansion* was checked.
- A Molecular Properties node was then added. The node was configured as follows: Under *Column Selection > Product* was selected from the dropdown menu, in the red *Exclude* box *Enforce exclusion* was checked. *Molar Mass* and *Molecular Formula* were added to the green *Include* box.
- An RDKit Canon SMILES node was added to generate canonical SMILES of products followed by a Column Filter node.
- Finally CSV Writer node and a Table to PDF node were included at the end of the workflow to save the generated data. The SMILES patterns and molecular weights of generated compounds

were then used for the downstream analysis of incubations with reactive fragments and recombinant protein

Active (D2B)	Functional Group	SMARTS Pattern
☐	AcidChloride	C(=O)Cl
☑	Aromatic Acid Chloride	[$(C-!@[a])](=O)(Cl)
☑	Aliphatic Acid Chloride	[$(C-!@[A;!Cl])](=O)(Cl)
☑	CarboxylicAcid	C(=O)[O;H,-]
☑	Aromatic Carboxylic Acid	[$(C-!@[a])](=O)([O;H,-])
☐	Aliphatic Carboxylic Acid	[$(C-!@[A;!O])](=O)([O;H,-])
☐	Alpha Amino Acid	[$(C-[C;!$(C=[!#6])]-[N;!H0;!$(N-[!#6;!#1]);!$(N-C=[O,N,S])])](=O)([O;H,-])
☑	SulfonylChloride	[$(S-!@[#6])](=O)(=O)(Cl)
☑	Aromatic Sulfonyl Chloride	[$(S-!@c)](=O)(=O)(Cl)
☑	Aliphatic Sulfonyl Chloride	[$(S-!@C)](=O)(=O)(Cl)
☐	Amine	[N;!H0;$(N-[#6]);!$(N-[!#6;!#1]);!$(N-C=[O,N,S])]
☐	Primary Amine	[N;H2;D1;$(N-!@[#6]);!$(N-C=[O,N,S])]
☑	Primary Aromatic Amine	[N;H2;D1;$(N-!@c);!$(N-C=[O,N,S])]
☐	Primary Aliphatic Amine	[N;H2;D1;$(N-!@C);!$(N-C=[O,N,S])]
☐	Secondary Amine	[N;H1;D2;$(N(-[#6])-[#6]);!$(N-[!#6;!#1]);!$(N-C=[O,N,S])]
☑	Secondary Aromatic Amine	[N;H1;D2;$(N(-[c])-[#6]);!$(N-[!#6;!#1]);!$(N-C=[O,N,S])]
☐	Secondary Aliphatic Amine	[N;H1;D2;$(N(-C)-C);!$(N-[!#6;!#1]);!$(N-C=[O,N,S])]
☑	Aromatic Amine	[N;!H0;$(N-c);!$(N-[!#6;!#1]);!$(N-C=[O,N,S])]
☐	Aliphatic Amine	[N;!H0;!$(N-c);$(N-C);!$(N-[!#6;!#1]);!$(N-C=[O,N,S])]
☐	Cyclic Amine	[N;!H0;R;$(N-[#6]);!$(N-[!#6;!#1]);!$(N-C=[O,N,S])]
☑	Boronic Acid	[$(B-!@[#6])](O)(O)
☑	Aromatic Boronic Acid	[$(B-!@c)](O)(O)
☑	Aliphatic Boronic Acid	[$(B-!@C)](O)(O)
☑	Isocyanate	[$(N-!@[#6])](=!@C=!@O)
☑	Aromatic Isocyanate	[$(N-!@c)](=!@C=!@O)
☑	Aliphatic Isocyanate	[$(N-!@C)](=!@C=!@O)
☐	Alcohol	[O;H1;$(O-!@[#6;!$(C=!@[O,N,S])])]
☐	Aromatic Alcohol	[O;H1;$(O-!@c)]
☐	Aliphatic Alcohol	[O;H1;$(O-!@[C;!$(C=!@[O,N,S])])]
☑	Aldehyde	[CH;D2;!$(C-[!#6;!#1])]=O
☑	Aromatic Aldehyde	[CH;D2;$(C-!@[a])](=O)
☑	Aliphatic Aldehyde	[CH;D2;$(C-!@C)](=O)
☐	Halogen	[$([F,Cl,Br,I]-!@[#6]);!$([F,Cl,Br,I]-!@C-!@[F,Cl,Br,I]);!$([F,Cl,Br,I]-[C,S](=[D1;O,S,N]))]
☐	Aromatic Halogen	[F,Cl,Br,I;$(*-!@c)]
☑	Aliphatic Halogen	[$([F,Cl,Br,I]-!@C);!$([F,Cl,Br,I]-!@C-!@[F,Cl,Br,I])]

☐	Not Fluorine Halogen	[$([Cl,Br,I]-!@[#6]);!$([Cl,Br,I]-!@C-!@[F,Cl,Br,I]);!$([Cl,Br,I]-[C,S](=[D1;O,S,N]))]
☑	Aliphatic Not Fluorine Halogen	[$([Cl,Br,I]-!@C);!$([Cl,Br,I]-!@C-!@[F,Cl,Br,I]);!$([Cl,Br,I]-[C,S](=[D1;O,S,N]))]
☐	Aromatic Not Fluorine Halogen	[$([Cl,Br,I]-!@c)]
☐	Bromine Halogen	[$([Br]-!@[#6]);!$([Br]-!@C-!@[F,Cl,Br,I]);!$([Br]-[C,S](=[D1;O,S,N]))]
☑	Aliphatic Bromine Halogen	[$(Br-!@C);!$(Br-!@C-!@[F,Cl,Br,I]);!$(Br-[C,S](=[D1;O,S,N]))]
☐	Aromatic Bromine Halogen	[$(Br-!@c)]
☑	Azide	[N;H0;$(N-[#6]);D2]=[N;D2]=[N;D1]
☑	Aromatic Azide	[N;H0;$(N-c);D2]=[N;D2]=[N;D1]
☑	Aliphatic Azide	[N;H0;$(N-C);D2]=[N;D2]=[N;D1]
☐	N-Carbonyl Aliphatic Amine	*C(=O)N(C(*)([H])[H])C(*)([H])[H]
☐	N-Boc Aliphatic Amine	*C([H])([H])N(C(=O)OC(C)(C)C)C(*)([H])[H]
☐	N-Acyl Aliphatic Amine	*C([H])([H])N(C(=O)C([H])([H])[H])C(*)([H])[H]
☐	N-Chloroacetamide Aliphatic Amine	*C([H])([H])N(C(=O)C[Br,I,Cl,F])C(*)([H])[H]
☑	Anhydride	[CX3](=[OX1])[OX2][CX3](=[OX1])
☑	Imide	[CX3](=[OX1])[NX3H0]([NX3H0]([CX3](=[OX1]))[CX3](=[OX1]))[CX3](=[OX1])
☐	Nitro	[$([NX3](=O)=O),$([NX3+](=O)[O-])][!#8]
☑	N-oxide	[$([#7+][OX1-]),$([#7v5]=[OX1]);!$([#7](~[O])~[O]);!$([#7]=[#7])]
☐	Aldehyde/formamide	[CX3H1](=O)[#6,#7]
☑	Imine	[$([CX3]([#6])[#6]),$([CX3H][#6])]=[$([NX2][#6]),$([NX2H])]
☑	Imide2	[CX3](=[OX1])[NX3H][CX3](=[OX1])
☑	Imide3	[CX3](=[OX1])[NX3H0]([#6])[CX3](=[OX1])
☐	Nitrate	[$([NX3](=[OX1])(=[OX1])O),$([NX3+]([OX1-])(=[OX1])O)]
☐	Isonitrile	[CX1-]#[NX2+]
☑	Hydrazine	[NX3][NX3]
☑	Hydrazone	[NX3][NX2]=[*]
☑	DiazoNitrogen	[$([#6]=[N+]=[N-]),$([#6-]-[N+]#[N])]
☐	Ketone	[#6][CX3](=O)[#6]
☑	Deuterium	[2H]
☑	13C	[13c,13C]
☑	15N	[15n,15N]
☐	Alkyl sulfide	[C][#16X2H0]
☑	Thiourea	[NX3][CX3]=[SX1][#6]
☑	Thioamide	[NX3][CX3]=[SX1]
☐	Carboxylate	[CX3](=[OX1])O
☐	aromatic lactone	[cX3](=[OX1])[oX2]
☑	aromatic enone	ccc=O
☑	acrylamide	[CX3]=C[CX3](=[OX1])[NX3]
☑	Oxime	[CX3](=N-O)
☑	Nitroso	[NX2](=O)
☑	Hydroxylamine	[NX3][OX2]
☑	Enamine	[n,NX3][CX3]=[CX3]
☐	Sulfone	[$([#16X4](=[OX1])(=[OX1])([#6])[#6]),$([#16X4+2]([OX1-])([OX1-])([#6])[#6])]
☑	Sulfinate	[$([#16X3](=[OX1])[OX2H0]),$([#16X3+]([OX1-])[OX2H0])]
☐	Sulfoxide	[$([#16X3]=[OX1]),$([#16X3+][OX1-])]

☑	Sulfonic acid and ester	[OX2,-]S(=O)(=O)(*)
☑	Thiol	[#16!H0]
☑	Isothiocyanate	[NX2]=[C]=[S]
☑	Azo	[NX2]=[NX2]
☑	Substituted alkene	[CX3]=C(C)(C)
☑	Amidine	[NX3][CX3]=[NX2]
☑	pyridinium eg	[n+]
☐	Chiral1	C[C@H](*)(*)
☐	Chiral2	C[C@@H](*)(*)
☐	Cyclic alkene removal	C[CX3]=C(c)(C)
☑	alkenyl halide	[F,Cl,Br,I][CX3]=[CX3]
☑	2-Halo pyridine/pyrimidine	c1([F,Cl,Br,I])[c,n]ccc[nH0]1
☑	2-Halo hetarenes	nc([F,Cl,Br,I])[c,n]
☑	1,2-diaminoarene	c([N;H2,H1])c[N;H2,H1]
☑	1,2-catechol	c([OH])c[OH]
☑	1,3-catechol	c([OH])[c,n]c[OH]
☑	1,3-diaminoarene	c([NH2])[c,n]c[NH2]
☑	1,4-catechol	c([OH])ccc[OH]
☑	thione	[#6]=S
☐	aniline (only)	cc([NH2])c
☑	ene-nitrile	[CX3]=[CX3]-C#N
☐	Substituted amide	[NX3][CX3]=[OX1]
☐	ethyl chain	[*][CH2][CH3]
☐	propyl chain	[*][CH2][CH2][CH3]
☑	sulfonyl halide	[$(S-!@[#6])](=O)(=O)[I,Br,Cl,F]
☑	zinc	[Zn]
☑	magnesium	[Mg]
☑	lithium	[Li]
☑	tin	[Sn]
☑	copper	[Cu]
☑	silicon	[Si]
☑	palladium	[Pd]
☑	boron	[B]
☑	Ruthenium	[Ru]
☑	Germanium	[Ge]
☑	1,2-dicarboxylic acid	c([$(C-!@[a])](=O)([O;H,-]))c([$(C-!@[a])](=O)([O;H,-]))
☑	1,3-dicarboxylic acid	c([$(C-!@[a])](=O)([O;H,-]))cc([$(C-!@[a])](=O)([O;H,-]))
☑	1,4-dicarboxylic acid	c([$(C-!@[a])](=O)([O;H,-]))ccc([$(C-!@[a])](=O)([O;H,-]))
☑	1,5-dicarboxylic acid	c([$(C-!@[a])](=O)([O;H,-]))cccc([$(C-!@[a])](=O)([O;H,-]))
☑	alkene	[c,n,C,N]C=C
☐	alkyne	[$([CX2]#C)]
☑	Hypervalent iodine (with I=O)	[I]=O
☐	Lactone	c[c,C](=O)O[c,C]
☑	Aromatic Urea	[#7][#6](=O)[#7]
☑	Urea	[NX3]C(=O)[NX3]
☑	Acetylenic Ester	[$([CX2]#C)]C(=[OX1])[OX2]C
☑	1,5 Hexadiyne	[$([CX2]#C)]CC[$([CX2]#C)]
☑	2,4 Hexadiyne	[$([CX2]#C)]C[$([CX2]#C)]
☑	Hexadiyne 2.0	[$([CX2]#C)][$([CX2]#C)]
☑	Heptdaiyne (O,N,C)	[$([CX2]#C)]C*C[$([CX2]#C)]

☑	1,5-diene	C=CCCC=C
☑	Phenyldialkyne 1,3	[$([CX2]#C)]ccc[$([CX2]#C)]
☑	Phenyldialkyne 1,4	[$([CX2]#C)]cccc[$([CX2]#C)]
☑	1,3-diene	C=CC=C
☑	1,4 diene	C=CCC=C
☑	Epoxide	C2OC2
☐	NH2	[NH2]
☐	Ene-dione	[CX3](=O)C=C[CX3](=O)
☑	Dihydropyrrole	C1C=CC[NH]1
☐	Acetal	CO[CX4]OC
☑	Pyran	Brcc([OX2])c
☑	Alpha-halo acid	[F,Cl,Br,I]cc([$(C-!@[a])](=O)([O;H,-]))
☑	Diacrylamide	C=CC(=O)N1CCN(C(=O)C=C)CC1
☑	Aldehyde 2.0	[CH](=O)[#6,#7]
☐	Amine Aromatic NH	[n;H1]
☐	Aliphatic NH	[C][NH1][C]
☐	Aniline NH	[c][NH1][*]
☐	Nitrile	[*][C](#N)
☑	Sulfomyl Chloride	[N][S](=O)(=O)(Cl)
☑	alpha Keto-Halides	[*][CX3](=[OX1])[#6][F,Cl,Br,I]
☑	Oxime 2	[#6][N](-O)[#6]
☑	Oxadiazole	[#6]N(-N=O-C(=C)-O)
☑	Methane Diamine	[#6][N][C][N][#6]
☑	Aromatic Methyldiamine	[n][C][N]
☑	Vinyl S(VI)	[C]=[C][S](=O)(=O)[#6,n,N]
☑	Enol Ethers	[C]=[C][O][#6]
☑	Imidates	[N]=[#6][O]
☑	Quat. Nitrogens	[$([NX4+])]
☑	Tertiary Sulfur	[S+](C)C
☑	Halogenated Succinamide	[N][Cl,Br]
☑	Phosphate Group	[OH][P](=O)[OH]
☑	Sulfonamide	[N][S](=O)(=O)
☑	beta-Keto Amide	[*][NH][#6](=O)[#6][#6](=O)[*]
☑	aplha-keto amide	[*][NH][#6](=O)[#6](=O)[*]
☑	DiAmine	[#7X3;H2,H1;!$(NC=O)].[#7X3;H2,H1;!$(NC=O)]
☑	Aromatic Diamine	[N][n]
☑	Sulfonamide	[#7][#16X4](=[OX1])=[OX1]

Table S3: Bespoke list of functional groups used for the RDKIt Functional Group filter. The list was used as a tab delimited .txt file (in the format Name\tSmarts\tLabel) to filter compounds. Functional groups that are checked did not pass the filter and were removed by the node.

Number	SMILES	Number	SMILES
**S2**	CNCc1cccnc1	**S58**	NCCCN1CCOCC1
**S3**	NCCN1CCOCC1	**S59**	N#CC1CCNCC1
**S4**	CNCc1ccc2c(c1)OCO2	**S60**	NCc1cc(F)cc(C(F)(F)F)c1
**S6**	CC(C)(C)OC(=O)N1CCCNCC1	**S61**	Cl.Fc1ccc(C2CCNCC2)cc1
**S7**	O=C1CNCCN1	**S62**	NC1CCN(Cc2ccccc2)CC1

**S9**	Cl.N#CC1(c2ccccc2)CCNCC1	**S63**	CNCc1ccc(C#N)cc1
**S10**	NCc1ccccc1N1CCCC1	**S64**	NC1CCOCC1
**S11**	C1CNCCOC1.Cl	**S65**	OC1(Cc2ccccc2)CCNCC1
**S12**	Cl.NCc1coc(-c2ccccc2)n1	**S66**	NCc1ccc(O)cc1
**S13**	O=C(c1ccccc1)N1CCNCC1	**S67**	OC1CCNCC1
**S14**	Cl.O=C1CC2(CCNCC2)CN1	**S68**	COc1ccc(CN)cc1
**S16**	COc1cc(CN)ccc1C#N	**S69**	NCc1cccc(N2CCCC2)c1
**S18**	N#Cc1ccc(C2(N)CC2)cc1	**S70**	c1csc(-c2noc(C3CCNCC3)n2)c1
**S19**	NCc1ccco1	**S71**	c1ccc(OC2CCNCC2)cc1
**S21**	Cn1c(CN)nc2ccccc21	**S72**	C1CC2CCC1N2.Cl
**S22**	O=C(c1ccco1)N1CCNCC1	**S73**	NC(c1ccccc1)c1ccccc1
**S25**	NCCN1CCCC1	**S74**	Cn1cc(CN)cn1
**S27**	OC[C@@H]1CCCN1	**S75**	CN1CCN(CCCN)CC1
**S28**	Br.Fc1ccc(N2C[C@@H]3C[C@H]2CN3)cc1	**S76**	COc1ccccc1CN
**S29**	COc1ccc(-c2nc(CN)co2)cc1	**S77**	N[C@@H]1C[C@H]2CC[C@@H]1C2
**S30**	N[C@@H](CO)c1ccccc1	**S78**	CC(=O)N1CCCNCC1
**S31**	NCCN1CCCCC1	**S79**	CNC1CCOCC1
**S32**	CC(C)N1CCNCC1	**S80**	CC(C)(C)OC(=O)N1CC[C@]2(CCNC2)C1
**S33**	CC(C)(C)OC(=O)N1CC(N)C1	**S81**	N#Cc1cccc(CN)c1
**S34**	CN[C@@H](C)c1ccccc1	**S82**	Cl.NCc1ccccc1Oc1ccccc1
**S35**	NC[C@H]1CN(Cc2ccc(F)cc2)CCO1	**S83**	Cl.NC[C@@H]1CCCC[C@H]1O
**S36**	CCOc1ccccc1N1CCNCC1	**S84**	NC1CCC(O)CC1
**S37**	NCCc1cccs1	**S85**	NCCc1ccc(O)cc1
**S38**	COc1ccc2c(c1)CCNC2	**S86**	COc1cc(CN)ccc1O.Cl
**S39**	NCc1ccccc1O	**S87**	Cl.NCc1cccc([N+](=O)[O-])c1
**S40**	c1cc(N2CCNCC2)ccn1	**S88**	CN(C)c1ccccc1CN
**S41**	O=C(OCc1ccccc1)N1CCNCC1	**S89**	CC(C)c1noc(C2CCNCC2)n1
**S42**	CC(=O)N1CCNCC1	**S90**	NCc1ccc(-n2cccn2)cc1
**S43**	O=C1CNCCN1Cc1ccccc1	**S91**	COc1ccc(CN)cc1OC
**S44**	N#Cc1ccc(N2CCNCC2)cc1	**S92**	c1cnc(N2CCNCC2)nc1
**S45**	CCOc1cccc(F)c1CN	**S93**	FC(F)(F)c1ccc(N2CCNCC2)nc1
**S46**	c1ccc2c(c1)CCNC2	**S94**	Cl.NC12CC3CC(CC(C3)C1)C2

**S47**	NCC(=O)NCc1cccc(Cl)c1	**S95**	C1CC2(CCN1)OCCO2
**S48**	C1NCC12COC2	**S96**	Cl.FC1(F)CCNC1
**S49**	NCc1ccccn1	**S97**	Cc1nc(C2CCNCC2)no1.Cl
**S50**	c1ccc2oc(C3CCNCC3)nc2c1	**S98**	Cl.c1cc2c(cc1C[C@H]1CCCNC1)OCO2
**S51**	Cl.N#Cc1ccccc1CN	**S99**	c1ccc(CN2CCNCC2)cc1
**S52**	COc1cc(F)ccc1CN	**S100**	CN1CCCNCC1
**S53**	NCc1cccs1	**S101**	Oc1ccc(N2CCNCC2)cc1
**S54**	Cl.c1cc2c(cn1)CNC2	**S102**	Cl.NCc1ccc([N+](=O)[O-])cc1
**S55**	COc1ccc(CN)c(OC)c1	**S103**	Cl.NC1CCC(F)(F)CC1
**S56**	Cn1cc(CN)c2ccccc21	**S104**	FC(F)(F)c1nnc2n1CCNC2
**S57**	NCc1ccsc1Br	**S105**	C1CC2CNCC1O2

Table S4: Selected amine substrates for the high-throughput synthesis and Direct-to-Biology workflow. Substrates that come before **S25** were also used in preliminary experiments (see Figure S1).

3.3 Automated analysis of high-throughput screening data to discovery chemical modifiers

Before the assay was performed, assay information was logged in a MongoDB noSQL database using custom Python scripts. The structure of data avoids data duplication and enables results to be filtered and compared across a series of protein targets.

(a) For each protein to be studied the following parameters were stored:

- Name and sequence metadata for the protein
- The retention time window that the protein eluted on the LC MS run, determined experimentally
- The deconvolution window for spectral deconvolution (typically a 10 kDa region spanning the protein and modified protein mass range)
- The molecular formula
- The peak integration window determined by simulating the isotopic distribution using the python package IsoSpecPy. From the theoretical isotopic pattern a low resolution envelope of the peak was created and the integration window defined between the points on the envelope where the intensity was 20% of the peak maximum. The integration window was then stored as the offset from the exact mass of the protein and was assumed to remain the same regardless of any modifications.

(b) An array of chemical modifiers was created by combining a “warhead” (protein-reactive electrophile) and a “selectivity element” (typically a group for non-covalent molecular recognition).

For each warhead the following information was stored:

- The warhead name
- The net change on addition of the warhead to a protein: For example, reaction of a sulfonyl fluoride leads to a net loss of HF from the warhead and protein combination. The code also recognised that warheads could lead to constant additions to a protein regardless of the structure of the modifier. Such changes were given a custom designation starting with a ^ symbol.

For each selectivity element the following information was stored:

- The selectivity element name

For each modifier the following information was stored:

- The SMILES of the modifier
- The database ID of the selectivity element
- The database ID of the warhead
- The formula of the modifier

(c) Modifiers and proteins were then combined into assay points

For each assay point the following information was stored:

- Metadata relating to experiment name and scientist
- The concentration of protein and modifier
- Incubation time
- The protein database ID
- The modifier database ID

Using appropriate joins, a view was then created that exposes, for each assay point, all the required details for MS processing and analysis (formulae of protein and modifiers; net change on modification; and retention time, deconvolution window and integration parameters)

The database ID of each assay point was logged and saved to an input sequence file for the mass spectrometer that stores the database ID for each assay point against the microtiter plate well index

(d) Data acquisition

The data was acquired on a Bruker Impact II QqTOF mass spectrometer using electrospray ionization. Samples were introduced from an Elute HT HPLC system equipped with a PAL autosampler. Short chromatography was performed using a Waters Acquity Vanguard Protein BEH C4 guard column 300A 1.7μm with dimensions 2.1mm x 5mm. The column was heated to 40 °C. Elution used a binary gradient made from A: Water containing 0.1% Formic Acid and B: MeCN containing 0.1% Formic Acid with linear interpolation between time points. The flow rate was 0.7 ml/min. Gradient details (balance is solvent B): Time 0 min A: 80%, 0.2 min A: 80%, 0.5 min A: 60%, 0.55 min A: 5%, 0.65 min A: 5%, 0.75 min A:

80%, 0.95 min A: 80%. Sodium formate was directly introduced into the source via a switching valve for the last 10 sec of acquisition to act as calibrant.

The sample overlapping option was employed to enable preparation of the next injection while the previous sample was running to reduce the total cycle time to 70 s per sample.

The sample list was created from the input file and contained the database ID of each assay point as a custom parameter for each well

(e) Data processing

In the Bruker DataAnalysis software with a MaxEnt deconvolution license, the following processing steps were performed using the custom processing script functions.

The data were calibrated using the data from the sodium formate-containing segment of each spectrum.

The processing parameters for each spectrum were retrieved from the database using a http request to the MongoDB Data API using the database ID parameter as a lookup key.

Spectra were then combined in the retention time window defined for the protein and the resulting data deconvolved in the range defined by the deconvolution window.

The theoretical exact masses of the un-, singly-, doubly- and triply-modified proteins were then calculated by combining the formulas of the protein and modifier and subtracting the change on reaction of the warhead. The resulting exact mass for each species was then created.

The deconvoluted spectra data were then integrated for each species (no modification, single modification, double modification etc) defined from the exact mass and the peak integration window that is defined at the protein level.

The total areas in each region were then sent to the database using an HTTP request alongside a low resolution (integer mass resolution) representation of the region of the spectrum containing protein and modified peaks

(f) Data visualization

Data was visualized for each sample using a custom Python script built using the Dash package. This enabled any of the data for the assay to be plotted alongside the representation of each spectrum, the theoretical location of each modified peak in the spectrum and the structure of the modifier.

3.4 MSConvert and UniDec Parameters for the Deconvolution of Protein Mass Spectra

The following workflow was utilised for the manual deconvolution of intact protein MS data collected for incubation reactions with Aurora A kinase and reactive fragment libraries.

- Raw intact protein MS data as .d files were converted to UniDec readable mzML files using ProteoWizard MSConvert (version 3) software.[11]
- Generated mzML files of MS spectra were loaded onto UniDec (version 7.02) in the UniChrome function and protein spectra were manually selected in the displayed chromatogram.
- Under *Peak Selection, Extraction, and Plotting* > *Peak Normalization* > *None* was checked and *Extract Normalization* > *None* was checked. All other parameters were left unchanged.
- The selected spectrum was then loaded onto the UniDec function by clicking *Open Selection in UniDec GUI*.
- The mass-to-charge range was set under *Data Processing* > *m/z: 500 to 1800* and *Use Background Subtraction* was checked, the data was then processed by clicking *Process Data*.
- Under *UniDec Parameters* the following parameters were used, *Charge Range: 10 to 100, Mass Range: 30000 to 40000 Da, Sample Mass Every (Da): 1.0*, the protein spectrum was then deconvoluted by clicking *Run UniDec*.
- Under *Peak Selection and Plotting* > *Peak Detection Range (Da)* was set to 50.0 and *Peak Detection Threshold* was set to 0.2 or set accordingly depending on peak height. *Peak Detection* was then clicked.
- Peaks were integrated by going to *Analysis* > *Integrate Peaks* (or ctrl + i).
- The amount of modified protein was calculated as a percentage using the following equation (where 'mod' stands for modified protein and 'unmod' stands for unmodified protein):

$$\% \, mod = \frac{Area_{mod}}{Area_{mod} + Area_{unmod}} \times 100$$

For the analysis of timecourse and kinetics data of protein modification reactions a bespoke python script (BafPipe by Dr. Lawrence Collins, https://github.com/lawrencecollins/BafPipe) was utilised with the following parameters (Table S5). The scripts uses UniDec to rapidly deconvolute large numbers of raw .d MS files.

Parameter	Input	Comments
Directory	C:\Users\[..]	
Start Scan	150	
End Scan	155	
Species Unlabelled	33715	Mass of unlabelled protein (Da)

Species Labelled	34068	Mass of labelled protein (Da)
Tolerance (Da)	5	Peak matching tolerance
Config masslb	30000	Deconvolution window low mass
Config massub	40000	Deconvolution window high mass
Config massbins	1	Mass bins for deconvolution - sample mass every
Config peakwindow	10	
Config peakthresh	0.075	
Config minmz	600	m/z lower bounds (defaults to 0)
Config maxmz	1800	m/z upper bounds (defaults to 10e12)
Config startz	1	
Config endz	100	
Config numz	100	
Config numit	60	number of iterations of deconvolution algorithm
Color Unlabelled	red	
Color Labelled	green	

Table S5: Deconvolawrence parameters used for deconvolution of large direct-to-biology datasets.

3.5 Covalent Docking of D2B Hit Fragments on Aurora A

Hits fragments for Aurora A kinase identified *via* D2B screens were uploaded onto Schödinger Maestro (Version 14.1.138) as a single .sdf file. Compounds were then prepared for docking using the built-in LigPrep function with preset factory settings. Aurora A kinase (PDB: 5ORL) was then loaded into the software *via* the Get PDB function under the File tab. The protein was prepared for docking using the built-in function, *Protein Preparation Workflow*, using preinstalled factory settings. A small molecule that labelled CYS247 *via* a disulfide bond was manually deleted from the structure. Covalent docking was then performed with the prepared fragments using the Covalent Docking (CovDock) function. Under *Receptor* > *Reactive Residue* was set to A:247, *Centroid of selected residue*s was selected with the following residue numbers: 246 (TYR), 190 (HIE), 280 (HIS), 272 (ILE), 275 (PHE), 184 (ILE), 188 (LEU), 193 (ILE), 252 (VAL).

Docking of fragment **F104** to Cys290 on Aurora A (PDB: 9SUY) was carried out following the same workflow and deleting fragment **F104** from the original PDB crystal structure. Under *Receptor* >

Reactive Residue was set to A:290, *Centroid of selected residue*s was selected with the following residue numbers: 335 (GLN), 287 (THR), 289 (LEU), 292 (THR), 296 (LEU), 300 (MET), 334 (TYR).

Under *Reaction Type* > *Reaction Type*, *Custom (from file)* was selected from the dropdown menu. A bespoke reaction for the substitution of fluorine on the perfluorobenzenesulfonamide with a cysteine thiol was uploaded under *Custom chemistry file*. The .cdock file was generated by editing the Nucleophilic Substitution reaction that comes preinstalled with the software. The resulting bespoke reaction file contained the following text:

```
# Nucleophilic substitution of a fluoride by a receptor thiol

# Receptor CYS, sulfur is <1>
RECEPTOR_SMARTS_PATTERN 2,[C,c]-[S;H1,-1]

# Ligand, heavily fluorinated aromatic ring, atom for substitution is <2>
LIGAND_SMARTS_PATTERN 1,[c]([F])

# Neutralize the CYS if necessary
CUSTOM_CHEMISTRY ("<1>",("charge",0,(1)))

# Add ligand-receptor single bond
CUSTOM_CHEMISTRY ("<1>|<2>",("bond",1,(1,2)))

# Remove the fluoride leaving group
CUSTOM_CHEMISTRY ("<2>[F]",("delete",2))
```

Docking simulations were run on all five carbon centres on the aromatic ring. Once completed, results for S_NAr reactions between cysteine thiol and reactive fragment on carbon-4 were then manually selected and reprocessed on PyMol.

3.6 FragPipe Workflow for Aurora A Kinase Peptide Mapping

Fragpipe (version 22.0) was used to localise modifications within peptides generated from bottom-up peptide mapping workflows on Aurora A kinase. MSFragger, IonQuant, diaTracer, DIA-NN, Python, Database Splitting and Spectral Library Generation add-ins were downloaded.

An initial search was carried out by modification of the factory-installed workflow 'Open' with the following settings:

- timsTOF LC-MS/MS data for Auora A kinase samples treated with the corresponding reactive fragment as a .d file was uploaded *Workflow* > *Input LC-MS Files, IM-MS (ion mobility, timsTOF only)* was checked
- Under *Workflow* > *Select a workflow, Open* was selected from the dropdown menu, the workflow was loaded by clicking *Load workflow*
- Under the *Database* tab the appropriate .fasta file was uploaded from UniProt (UP000005640 Homo sapiens (Human)) and decoys were added by clicked the *Add decoys* button.
- Under the *MSFragger* tab *Run MSFragger* was checked, and in *Common Options* > *Peak Matching* > *Precursor mass tolerance*, *Da* was selected from the dropdown menu and the range was set to *-150 - 1,000* and *Fragment mass tolerance* was set to *20 PPM*. *Protein Digestion* under *Enzyme name 1, trypsin* was added. Under *No Cuts 1*, *P* was added and under *Mixed Cleavages 1*, *2* was added. Under *Enzyme name 2, lysc* was added. Other settings in this section remained unchanged.
    - In *Modifications* > *Variable Modifications* and *Fixed Modifications* all boxes remained unchecked. Other settings in this section remained unchanged.
    - In *Advanced Options* and *Spectral Processing* all settings were left unchanged.
    - In *Open Search Options, Yes, keep delta mass* was selected from the dropdown menu under *Report mass shift as a variable mod* and *Localize mass shift (LOS)* was checked.

- In *Advanced Peak Output Options*, *Write calibrated mzML* was checked. All other settings in this tab remained unchanged.

- Under *Validation* > *Crystal-C* was unchecked and in the same tab under *FDR Filter and Report, Remove contaminants was checked*. All other settings in this tab remained unchanged.
- All other settings remained unchanged and the software was made to run by clicking *RUN* in the *Run* tab.

The output data was then analysed manually by opening the psm.tsv file and looking for the mass of the incubated small molecule in the *Delta Mass* column. Observed mass modifications were then added to a new workflow for label-free quantification. Datasets for triplicate control samples were run with triplicate samples treated with a corresponding compound. This was repeated for all 6 reactive fragments tested. The previous workflow was modified as follows for LFQ quantification of labelled peptides:

- Under the *MSFragger* > In *Common Options* > *Peak Matching* > *Precursor mass tolerance*, *Da* was changed to *-10 - 10* and *Fragment mass tolerance* was set to *20 PPM*. *Isotope error* was set to *0/1*. Other settings in this section were left unchanged.
  - Under *Modifications* > *Fixed modification*: *C (cysteine), S (serine), T (threonine), K (lysine), H (histidine), R (arginine), Y (tyrosine)* were unchecked. All other settings remained unchanged. Fragpipe was then run with the following modified settings.
  - Under *Advanced options* > *Mass Offsets* the following masses were added in the appropriate text box: -105.0248 -89.0299 -33.9877 -32.0085 -30.0106 -18.0106 -17.0265 -2.0157 -1.007825 -0.984 0 0.984 3.9949 12.0 13.9793 14.0157 15.9949 19.9898 21.969392 21.9819 23.95806 26.0157 27.9949 28.0313 28.990164 29.9742 31.972071 31.9898 37.9469 37.955882 42.0106 42.047 43.0058 43.9898 44.985078 47.9847 53.9193 57.02146 58.0055 61.9135 61.921774 68.026215 70.041865 71.0371 79.9568 79.9663 86.000394 100.016 114.042927 119.004099 128.095 146.0579 156.1011 162.0528 173.051 176.0321 178.0477 183.035399 189.046 203.0794 204.1878 210.1984 228.111 229.014009 238.2297 301.9865 340.1006 349.1373 365.1322 365.1322 406.1587 541.06111 [input fragment mass shift]. The corresponding mass change of the reactive fragment was added at the end of the list.
- Fragment mass shifts = **F7**: 310.0062; **F13**: 400.0433; **F43**: 400.0578; **F100**: 324.0578; **F101**: 388.00660; **F104**: 402.0006.

- Under *PTMs* > *PTM Profiling*, *Max fragment charge* was set to *2*, *Precursor tolerance* was set to *8 PPM*, Peak picking with was set to *3 PPM*. Under *Annotation* > *Annotation Source*, *Common mass shifts* was selected.
- For Label-free Quantification, under *Quant (MS1)* > *MS1 Quantification*, *Run MS1 quant* and *IonQuant* were selected. Under *Common, match between runs (MBR)* was checked and *Normalise intensities across runs* was checked. Under *Advanced options* > *Match between runs (MBR)* > *MBR Top runs* was set to *2*
- All other settings remained unchanged, and the software was run.

For this search, peptide data was visualised using the built-in tool under the *Run* tab and by clicking the *Open Fragpipe-PDV viewer* option. The output *combined_ion.tsv* file was then processed to calculate the total intensities of modified residues and % modification relative to the total amount of modified peptides. Only peptides with robust MS2 fragmentation and reliable localisation of modified amino acid residues were included in calculations.

4. Chemistry Experimental

General Experimental Considerations

Commercially available starting materials were obtained from Sigma–Aldrich, Fluorochem, Alfa Aesar, BLDpharm, Santa Cruz Biotechnology and Apollo Scientific. Water-sensitive reactions were performed under nitrogen in oven-dried glassware cooled under nitrogen before use. Anhydrous trimethylamine was obtained from a SureSeals bottle supplied by Sigma–Aldrich. All other solvents used were of chromatography or analytical grade. An IKA RV 10 rotary evaporator was used to remove solvents under reduced pressure.

Thin layer chromatography (TLC) was performed using glass-backed silica (Merck silica gel 60 F254) plates obtained from Merck. Ultraviolet lamp (λ_{max} = 254 nm) and/or $KMnO_4$ stain was used for visualization. R_F values are only reported for compounds that were purified by flash column chromatography. Flash column chromatography was performed using silica gel 60 (35-70 µm particles) supplied by Merck. Accurate Mass spectra were acquired on a Bruker Impact II QqTOF spectrometer equipped with a VIPHESI source using either electrospray or atmospheric pressure chemical ionisation.

Samples were introduced using an HTC PAL autosampler and Bruker Elute Pump. HPLC columns were heated to 40 degrees Celsius unless otherwise stated. Samples passed through a Bruker Diode array UV-detector before entering the mass spectrometer. Perkin-Elmer One FT-IR spectrometer was used to analyse the infrared spectra.

Mass directed auto-purification (MDAP-HPLC) of reactions was performed using an Agilent 1290 Infinity II Preparative HPLC system with mass spectrometer (LC/MSD XT) and fraction collector. The system ran in positive mode with an Agilent Technologies PLRP-S, 300Å, 8 µM particle size, 150x25 mM column at ambient temperature with a binary solvent system: MeCN and H_2O with 0.1% formic acid. The flow rate was set to 21.09 mL/min.

Proton (^1H), carbon (^{13}C) and (^{19}F) NMR data was collected on a Bruker 400 or 500 MHz spectrometer. Data was collected at 298 K unless otherwise stated. Chemical shifts (δ) are given in parts per million (ppm) and they are referenced to the residual solvent peak. Coupling constants (*J*) are reported in Hertz (Hz) and splitting patterns are reported in an abbreviated manner: app. (apparent), s (singlet), d (doublet), t (triplet), q (quartet), m (multiplet), br. (broad). Assignments were made using COSY, HSQC, HMBC and NOESY experiments. The ^{13}C NMR spectra of perfluorophenyl-substituted compounds were complicated by fluorine-carbon coupling. The signals for carbon atoms on these substituents were complicated by coupling to ^{19}F (typically: $^1J_{CF}$ 255–265 Hz; $^2J_{CF}$ 13–15 Hz; $^3J_{CF}$ 1–6 Hz). In some cases, not all splitting patterns could be assigned, and are marked (†). Signals that were not observed are noted; in these cases, the presence of fluorine was confirmed by ^{19}F NMR spectroscopy. Diastereotopic protons are noted with superscripts a and b (e.g. -H^a). Rotamers are noted with subscripts A and B.

4.1 Fragment Stability and Reactivity Assays

Analysis was performed using an Agilent 1290 Infinity II HPLC system (Agilent, Santa Clara, CA, United States), with a diode array detector. Chromatographic separations were performed using an Agilent InfinityLab Poroshell 120 EC-C18 (2.1 x 50 mm, 2.7 um) at a column temperature of 40 °C. The mobile phase used was A - TRIS buffer (95 %) and B - acetonitrile (5%) and the gradient running to 5 % TRIS

buffer over 5 minutes at a flow rate of 0.5 mL/min. The DAD recorded the chromatogram at a wavelength of 254 nm.

Compounds **F7** and **F104** (5 µL, 20 mM in DMSO) were transferred to LC-MS vials together with 1,4-dicyanobenzene (5 µ, 20 mM in DMSO) as an internal standard. TRIS buffer was then added (490 µL) so that the final concentration of compound and internal standard was 200 µM with 2% DMSO. Samples were then analysed every 50 minutes over a 24 hour time period.

The UV peak areas corresponding to the compound and internal standard (1,4-dicyanobenzene) were integrated using the instrument software and processed in Excel. For each time point, the total peak area of the compound was divided by the total peak area of the internal standard to give a *Probe/Standard* ratio. This ratio was then normalised to the maximum value observed across the dataset to account for variations in injection volume and detector response. The resulting *Normalised* values were plotted against time in OriginPro, no decomposition was observed at 24 hours for both compounds (see Figure S11).

The assay was also used to assess inherent reactivity with amino acids. Compounds (5 µL, 20 mM in DMSO) were transferred to LC-MS vials together with 1,4-dicyanobenzene (5 µL, 20 mM in DMSO) as an internal standard. TRIS buffer was then added (480 µL) so that the final concentration of compound and internal standard was 200 µM with 2% DMSO. The pH of incubations was fixed at 7.5 for *N*-acetyl-L-cysteine methyl ester and 10.7 for *N*-acetyl- L-lysine methyl ester. Either *N*-acetyl- L-cysteine methyl ester or *N*-acetyl- L-lysine methyl ester (10 µL, 50 mM in TRIS) was added to corresponding samples so that the final concentration of nucleophile was 1 mM (10 equiv.). Samples were then analysed immediately and then every 30 min for 6 hr.

4.2 General Procedure B – Re-synthesis of Direct-to-Biology Hit Compounds

Hits from protein labelling experiments were resynthesized in a PTFE custom made 96-well plate (8 × 12) with inserts for borosilicate glass vials (vial volume = 750 µL, vial dimensions = 8 × 30 mm, CV-2100-0830 Chemglass). Stock solutions of triethylamine (0.66 M in MeCN), perfluorobenzenesulfonyl chloride (0.6 M in MeCN) and corresponding amines (0.6 M in MeCN) were prepared. Stock solutions of amines that were salts were dissolved in a solution of triethyl amine (0.6 M in MeCN). A magnetic follower was added to each vial followed by a solution of triethylamine (100 µL, 0.66 M). A solution of the corresponding amine substrate was then added (100 µL, 0.6 M) followed by a solution of perfluorobenzenesulfonyl chloride (100 µL, 0.6 M). All reagents were added to the corresponding vials

as solutions in acetonitrile using pipettes. The final concentration of reagents in the plate was as follows: triethylamine (0.22 M, 1.2 equiv); amine substrate (0.2 M, 1 equiv); perfluorobenzenesulfonyl chloride (0.2 M, 1 equiv) in a total reaction volume of 300 µL. The glass vials were then capped and the reactions were allowed to stir at room temperature for 24 hr. The solvent was then removed under reduced pressure in a desiccator. The resulting crude residue was dissolved in a mixture of acetone and hexane (1:1) for purification by flash silica-gel column chromatography.

4.3 Compound synthesis

tert-Butyl 4-((perfluorophenyl)sulfonyl)-1,4-diazepane-1-carboxylate (F6)

Prepared according to General Procedure B with substrate S6. The crude reaction was purified by flash column chromatography, eluting with acetone/hexane 10:90. The *carboxylate* was isolated as a colourless solid (15.9 mg, 61%) containing two rotamers (a and b). R_F = 0.93 (acetone/hexane 20:80). ν_{max}/cm^{-1} 2975, 2933, 1689, 1644, 1520, 1492, 1413, 1365, 1298, 1252, 1233, 1163, 1097, 988, 912,

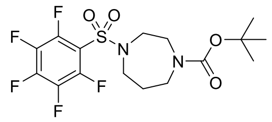

881, 773, 730, 702, 597 and 576. δ_H (500 MHz, $CDCl_3$) 3.59-3.51 (4H, m, 2- and 7-H_2), 3.47 (2H, t, *J* 5.0 Hz, 3-H_2), 3.40 (2H, t, *J* 6.0 Hz, 5-H_2), 2.02-1.97 (2H, m, 6-H_2), 1.46 (9H, s, tert-butyl H_9). δ_C (125 MHz, $CDCl_3$) 155.2 (carbonyl$_a$), 154.8 (carbonyl$_b$), 144.6 (ddq, *J* 263.0, 13.0 and 4.4 Hz, perfluorophenyl C-2 and -6), 143.8 (dtt, *J* 263.7, 13.4 and 5.4 Hz, perfluorophenyl C-4), 138.0 (dtt, *J* 258.1, 12.6 and 5.2 Hz, perfluorophenyl C-3 and -5), 116.5-116.1 (m†, perfluorophenyl C-1), 80.4 (tert-butyl C-2$_a$), 80.3 (tert-butyl C-2$_b$), 50.5 (C-3$_a$), 50.3 (C-3$_b$), 49.4 (C-2$_b$ or C-7$_b$), 49.3 (C-2$_a$ or C-7$_a$), 48.2 (C-5$_a$), 48.0 (C-5$_b$), 46.2 (C-2$_a$ or C-7$_a$), 45.7 (C-2$_b$ or C-7$_b$) 28.5 (tert-butyl C_3-1), 28.3 (C-6). δ_F (376 MHz, $CDCl_3$) –136.08- –133.13 (2F, m, perfluorophenyl 3- and 5-F or 2- and 6-F), –146.08- –146.19 (1F, m, perfluorophenyl 4-F), –158.33- –158.43 (2F, m, perfluorophenyl 3- and 5-F or 2- and 6-F). HRMS: $C_{16}H_{19}F_5N_2O_4S$ [M+Na]; requires: 453.0878; found: 453.0875.

4-((Perfluorophenyl)sulfonyl)piperazin-2-one (F7)

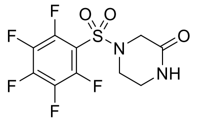

Prepared according to General Procedure B with substrate S7. The crude reaction was purified by flash column chromatography, eluting with acetone/hexane 50:50, to give the *piperazinone* as a colourless solid (11.6 mg, 58%). R_F = 0.55 (acetone/hexane 50:50). ν_{max}/cm^{-1} 2924, 1670, 1520, 1491, 1371, 1300, 1174, 1105, 988, 945, 669, 613 and 544. δ_H (500 MHz, CD_3OD) 3.93 (2H, s, 3-H_2), 3.59-3.57 (2H, m, 5-H_2), 3.43-3.41 (2H, m, 6-H_2). δ_C (125 MHz, CD_3OD) 167.8 (C-2), 48.4 (C-3) 43.3 (C-5), 41.9 (C-5). Fluorinated carbons not visible due to low sample concentration. δ_F (376 MHz, CD_3OD) –134.64- –134.70 (m, perfluorophenyl 3- and 5-F or 2- and 6-F), –143.39- –144.07 (m, perfluorophenyl 4-F), –157.36- –157.46 (m, perfluorophenyl 3- and 5-F or 2-and 6-F). Integrations not calculated due to low sample concentration and high background noise. HRMS: $C_{10}H_7F_5N_2O_3S$ [M+H] requires: 331.0170; found: 331.0171.

(4-((Perfluorophenyl)sulfonyl)piperazin-1-yl)(phenyl)methanone (F13)

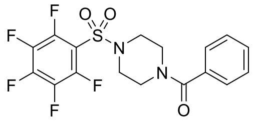

Prepared according to General Procedure B with substrate S13. The crude reaction was purified by flash column chromatography, eluting with acetone/hexane 30:70, to give the *methanone* as a colourless solid (3.7 mg, 13%). R_F = 0.16 (acetone/hexane 20:80). ν_{max}/cm^{-1} 2931, 1641, 1521, 1495, 1428, 1371, 1285, 1264, 1180, 1115, 1098, 991, 945, 730, 711, 598 and 576. δ_H (500 MHz, $CDCl_3$) 7.47-7.36 (5H, m, phenyl 2- and 3- and 4- and 5- and 6-H), 3.85-3.65 (4H, m, piperazinyl 2- and 6-H_2), 3.32 (4H, bs, piperazinyl 3- and 5-H_2). δ_C (125 MHz, $CDCl_3$) 170.8 (carbonyl), 144.8 (d†, perfluorophenyl C-2 and -6), 144.2 (d†, perfluorophenyl C-4), 138.4 (d†, perfluorophenyl C-3 and -5), 134.6 (phenyl C-1), 113.6 (m†, perfluorophenyl C-1), 130.6 (phenyl C-4), 128.9 (phenyl C-2 and -6 or C-3 and -5), 127.3 (phenyl C-3 and -5 or C-2 and -6), 45.9 (piperazinyl C-3 and -5). Rotameric piperazinyl carbons 2 and 6 could not be identified with confidence, these could be broad peaks at 47.3 ppm or 41.8 ppm. δ_F (376 MHz, $CDCl_3$) –134.15 - –134.20 (2F, m, perfluorophenyl 3- and 5-F or 2- and 6-F), –144.20 - –144.31 (1F, m, perfluorophenyl 4-F), –157.57 - –157.67 (2F, m, perfluorophenyl 3- and 5-F or 2- and 6-F). HRMS: $C_{17}H_{13}F_5N_2O_3S$ [M+H] requires: 421.0640; found: 421.0638.

Furan-2-yl(4-((perfluorophenyl)sulfonyl)piperazin-1-yl)methanone (F22)

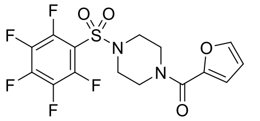

Prepared according to General Procedure B with substrate S22. The crude reaction was purified by flash column chromatography, eluting with acetone/hexane 20:80, to give the *methanone* as a colourless solid (10.5 mg, 43%). R_F = 0.15 (acetone/hexane 20:80). ν_{max}/cm^{-1} 2922, 1633, 1619, 1518, 1492, 1480, 1426, 1369, 1288, 1270, 1172, 1117, 1094, 1011, 988, 946, 760, 732, 604 and 574. δ_H (500 MHz, $CDCl_3$) 7.49 (1H, app. d, *J* 0.9 Hz, furanyl 5-H), 7.07 (1H, dd, *J* 3.4 and 0.5 Hz, furanyl 3-H), 6.50 (1H, dd, *J* 3.5 and 1.8 Hz, furanyl 4-H), 3.95 (4H, br. s, piperazinyl 2- and 6-H_2), 3.37-3.35 (4H, m, piperazinyl 3- and 5-H_2). δ_C (125 MHz, $CDCl_3$) 159.1 (carbonyl), 147.4 (furanyl C-2), 144.83 (ddq, *J* 263.0, 13.0 and 4.4 Hz, perfluorophenyl C-4), 144.3 (dtt, *J* 263.7, 13.4 and 5.4 Hz, perfluorophenyl C-4), 144.3 (furanyl C-5), 138.2 (dtt, *J* 258.1, 12.6 and 5.2 Hz, perfluorophenyl C-3 and -5), 117.9 (furanyl C-3), 113.4 (app. tt, *J* 15.2 and 2.2 Hz, perfluorophenyl C-1), 111.8 (Furanyl C-4), 46.0 (piperazinyl C-3 and -5), 44.0 (app. br. s, piperazinyl C-2 and -6) . δ_F (376 MHz, $CDCl_3$) –134.04 - –134.10 (2F, m, perfluorophenyl 3- and 5-F or 2- and 6-F), –144.25 - –144.36 (1F, m, perfluorophenyl 4-F), –157.60- –157.70 (2F, m, perfluorophenyl 3- and 5-F or 2- and 6-F). HRMS: $C_{15}H_{11}F_5N_2O_4S$ [M+H] requires: 411.0432; found: 411.0431.

Benzyl 4-((perfluorophenyl)sulfonyl)piperazine-1-carboxylate (F41)

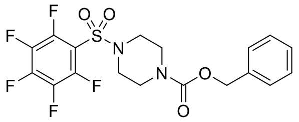

Prepared according to General Procedure B with substrate S41. The crude reaction was purified by flash column chromatography, eluting with acetone/hexane 20:80, to give the *carboxylate* as a colourless solid (20.6 mg, 76%). R_F = 0.27 (acetone/hexane 20:80). ν_{max}/cm^{-1} 2931, 2867, 1701, 1643, 1520, 1494, 1429, 1375, 1284, 1222, 1180, 1130, 1098, 990, 951, 764, 730, 715, 699, 597, 576 and 553. δ_H (500 MHz, $CDCl_3$) 7.38-7.31 (5H, m, benzyl, 2- and 3- and 4- and 5- and 6-H), 5.12 (2H, s, benzylic H_2), 3.26 (4H, br. s, 2- and 6-H_2), 3.65-3.63 (4H, m, 3- and 5-H_2). δ_C (125 MHz, $CDCl_3$) 154.9 (carbonyl), 144.8 (ddq, *J* 263.0, 13.0 and 4.4 Hz, perfluorophenyl C-2 and -6), 144.2 (dtt, *J* 263.7, 13.4 and 5.4 Hz, perfluorophenyl C-4), 138.4 (dtt, *J* 258.1, 12.6 and 5.2 Hz, perfluorophenyl C-3 and -5), 136.2 (phenyl C-1) 128.7 (benzyl C-3 and -5) 128.5 (benzyl C-4), 128.3 (benzyl C-2 and -6) 113.5 (app. tt, *J* 15.2 and 2.2 Hz, perfluorophenyl C-1) 67.8 (benzylic C), 45.6 (C-3 and -5), 43.6 (C-2 and -6). δ_F (376 MHz, $CDCl_3$) –134.04- –134.10 (2F, m, perfluorophenyl 3- and 5-F or 2- and 6-F), –144.25- –144.36 (1F, m, perfluorophenyl 4-F), –157.60- –157.70 (2F, m, perfluorophenyl 3- and 5-F or 2- and 6-F). HRMS: $C_{18}H_{15}F_5N_2O_4S$ [M+H] requires: 451.0745; found: 451.0744.

1-Benzyl-4-((perfluorophenyl)sulfonyl)piperazin-2-one (F43)

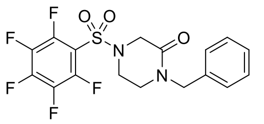

Prepared according to General Procedure B with substrate S43. The crude reaction was purified by flash column chromatography, eluting with acetone/hexane 30:70, to give the *piperazinone* as a colourless solid (16.9 mg, 67%). R_F = 0.16 (acetone/hexane 20:80). ν_{max}/cm^{-1} 1658, 1521, 1495, 1380, 1356, 1298, 1176, 1099, 990, 966, 666 and 578. δ_H (500 MHz, $CDCl_3$) 7.35-7.29 (3H, m, benzyl 3- and 5-H and 4-H), 7.23-7.21 (2H, m, benzyl 2- and 6-H), 4.61 (2H, s, benzylic H_2), 4.05 (2H, s, 3-H_2), 3.58-3.56 (2H, t, *J* 5.1 Hz, 5-H_2), 3.58-3.56 (2H, t, *J* 5.1 Hz, 6-H_2). δ_C (125 MHz, $CDCl_3$) 163.0 (C-2), 144.8 (ddq, *J* 263.0, 13.0 and 4.4 Hz, perfluorophenyl C-2 and -6), 144.4 (dtt, *J* 263.7, 13.4 and 5.4 Hz, perfluorophenyl C-4), 138.1 (dtt, *J* 258.1, 12.6 and 5.2 Hz, perfluorophenyl C-3 and -5), 135.7 (benzyl C-1), 129.0 (benzyl C-3 and -5 or C-2 and -6), 128.4 (benzyl C-2 and -6 or C-3 and -5), 128.23 (benzyl C-4), 113.9-113.6 (app. tt, *J* 15.2 and 2.2 Hz, perfluorophenyl C-1), 50.2 (benzylic C), 48.1 (C-3), 45.6 (C-6), 43.1 (C-5). δ_F (376 MHz, $CDCl_3$) –134.39- –134.44 (2F, m, perfluorophenyl 3- and 5-F or 2- and 6-F), –143.77- –143.88 (1F, m, perfluorophenyl 4-F), –157.38 - –157.51 (2F, m, perfluorophenyl 3- and 5-F or 2- and 6-F). HRMS: $C_{17}H_{13}F_5N_2O_3S$ [M+H] requires: 421.0640; found: 421.0637.

4-(4-((Perfluorophenyl)sulfonyl)piperazin-1-yl)phenol (F101)

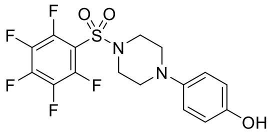

Prepared according to General Procedure B with substrate F101. The crude reaction was purified by reverse phase MDAP-HPLC eluting with H_2O/CH_3CN (with 0.1% formic acid) over 12 min to give the *phenol* as a colourless solid (1.4 mg, 6%). ν_{max}/cm^{-1} 1659, 1516, 1496, 1370, 1264, 1232, 1179, 989, 948, 732, 643, 621, 599, 575, 532, 483 and 457. δ_H (500 MHz, $CDCl_3$) 6.84-6.81 (2H, m, 2- and 6-H or 3- and 5-H), 6.79-6.76 (2H, m, 3- and 5-H or 2- and 6-H), 4.52 (1H, br. s, O*H*), 3.44-3.43 (4H, m, piperazinyl 3- and 5-H_2 or 2- and 6-H_2), 3.16-3.14 (4H, m, piperazinyl 2- and 6-H_2 or 3- and 5-H_2). δ_C (125 MHz, $CDCl_3$) 150.8 (C-1 or C-4), 145.0 (C-4 or C-1), 119.8 (C-2 and -6 or C-3 and -5), 116.2 (C-2 and -6 or C-3 and -5), 51.0 (piperazinyl C-2 and -6 or C-3 and -5), 46.0 (piperazinyl C-3 and -5 or C-2 and -6). Fluorinated carbons not visible due to low sample concentration. δ_F (376 MHz, $CDCl_3$) –133.92- –133.98 (m, perfluorophenyl 3- and 5-F or 2- and 6-F), –144.85- –144.96 (m, perfluorophenyl 4-F), –157.98- –158.08 (m, perfluorophenyl

3- and 5-F or 2- and 6-F). Fluorine integrations not calculated due to low sample concentration and high background noise. HRMS: $C_{16}H_{13}F_5N_2O_3S$ [M+H] requires: 409.0640; found: 409.0639.

1-Benzyl-4-((perfluorophenyl)sulfonyl)piperazine (F99)

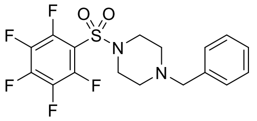

Prepared according to General Procedure B with substrate S99. The crude reaction was purified by flash column chromatography, eluting with acetone/hexane 10:90, to give the *piperazine* as a colourless solid (39.7 mg, >99%). R_F = 0.39 (acetone/hexane 20:80). ν_{max}/cm^{-1} 2922, 2817, 2776, 1643, 1519, 1491, 1454, 1370, 1296, 1179, 1128, 1097, 989, 956, 731, 700, 651, 596, 575 and 527. δ_H (500 MHz, $CDCl_3$) 7.32-7.29 (2H, m, benzyl 3- and 6-H), 7.27-7.26 (3H, m, benzyl 2- and 6-H and 4-H), 3.53 (2H, s, benzylic-H_2), 3.29 (4H, app. br. s, 3- and 5-H_2), 2.57-2.55 (4H, m, 2- and 6-H_2). δ_C (125 MHz, $CDCl_3$) 144.8 (ddq, *J* 263.0, 13.0 and 4.4 Hz, perfluorophenyl C-2 and -6), 144.0 (dtt, *J* 263.7, 13.4 and 5.4 Hz, perfluorophenyl C-4), 138.1 (dtt, *J* 258.1, 12.6 and 5.2 Hz, perfluorophenyl C-3 and -5), 137.4 (benzyl C-1), 129.2 (benzyl C-2 and -6 or C-3 and -5), 128.6 (benzyl C-3 and -5 or C-2 and -6), 127.6 (benzyl C-4), 113.6 (t†, *J* 15.2 Hz, perfluorophenyl C-1), 62.6 (benzylic C), 52.1 (C-2 and -6), 46.0 (C-3 and -5). δ_F (376 MHz, $CDCl_3$) –133.88- –133.93 (2F, m, perfluorophenyl 3- and 5-F or 2- and 6-F), –145.18- –145.29 (1F, m, perfluorophenyl 4-F), –158.18- –158.28 (2F, m, perfluorophenyl 3- and 5-F or 2 and 6-F). HRMS: $C_{17}H_{15}F_5N_2O_2S$ [M+H] requires: 407.0847; found: 407.0841.

1-Methyl-4-((perfluorophenyl)sulfonyl)-1,4-diazepane (F100)

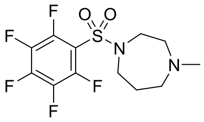

Prepared according to General Procedure B with substrate S100. The crude reaction was purified by flash column chromatography, eluting with acetone/hexane 60:40, to give the *diazepane* as a colourless solid (13.5 mg, 40%). R_F = 0.25 (acetone/hexane 50:50). ν_{max}/cm^{-1} 2953, 2797, 1644, 1518, 1496, 1352, 1295, 1164, 1102, 991, 598 and 578. δ_H (500 MHz, $CDCl_3$) 3.56-3.51 (4H, m, 3- and 5-H_2), 2.71-2.69 (2H, m, 2-H_2), 2.67-2.65 (2H, m, 7-H_2), 2.39 (3H, s, methyl), 1.96-1.91 (2H, m, 6-H_2). δ_C (125 MHz, $CDCl_3$) 144.8 (ddq, *J* 263.0, 13.0 and 4.4 Hz, perfluorophenyl C-2 and -6), 143.7 (dtt, *J* 263.7, 13.4 and 5.4 Hz, perfluorophenyl C-4), 138.0 (dtt, *J* 258.1, 12.6 and 5.2 Hz, perfluorophenyl C-3 and -5) 116.2 (app. tt, *J* 15.2 and 2.2 Hz, perfluorophenyl C-1), 58.9 (C-2), 56.8 (C-7), 48.4 (C-3 or C-5), 47.3 (C-5 or C-3), 46.7

(methyl C), 27.9 (C-6). δ_F (376 MHz, $CDCl_3$) –135.66- –135.72 (2F, m, perfluorophenyl 3- and 5-F or 2- and 6-F), –146.37- –146.48 (1F, m, perfluorophenyl 4-F), –158.51- –158.61 (2F, m, perfluorophenyl 3- and 5-F or 2- and 6-F). HRMS: $C_{12}H_{13}F_5N_2O_2S$ [M+H] requires: 345.0691; found: 345.0687.

7-((Perfluorophenyl)sulfonyl)-3-(trifluoromethyl)-5,6,7,8-tetrahydro-[1,2,4]triazolo[4,3-a]pyrazine (F104)

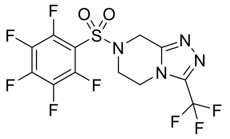

Prepared according to General Procedure B with substrate S104. The crude reaction was purified by flash column chromatography, eluting with acetone/hexane 30:70, to give the *pyrazine* as a colourless solid (16.8 mg, 66%). R_F = 0.17 (acetone/hexane 20:80). ν_{max}/cm^{-1} 1497, 1264, 1143, 991, 733, 704, 596 and 575. δ_H (500 MHz, $CDCl_3$) 4.85 (2H, s, 8-H_2), 4.31 (2H, t, *J* 5.4 Hz, 5-H_2), 3.92 (2H, t, *J* 5.4 Hz, 6-H_2). δ_C (125 MHz, $CDCl_3$) 148.5 (C-8a), 144.9 (d†, *J* 263.0 Hz, perfluorophenyl C-2 and -6), 144.0 (q, *J* 41.0 Hz, C-3), 138.4 (dtt, *J* 258.1, 12.6 and 5.2 Hz, perfluorophenyl C-3 and -5), 118.2 (q, *J* 270.7 Hz, trifluoromethyl C), 114.2 (app. tt, *J* 15.2 and 2.2 Hz, perfluorophenyl C-1), 44.0 (C-5), 42.6 (C-8 or C-6), 42.5 (C-6 or C-8). The fluorinated carbon in position C-4 on the perfluorophenyl was not visible in the ^{13}C NMR and was not assigned. δ_F (376 MHz, $CDCl_3$) –63.0 (3F, s, trifluoromethyl F_3), –135.10- –135.15 (2F, m, perfluorophenyl 3- and 5-F or 2- and 6-F), –142.81- –142.92 (1F, m, perfluorophenyl 4-F), –156.81- –156.97 (2F, m, perfluorophenyl 3- and 5-F or 2- and 6-F). HRMS: $C_{12}H_6F_8N_4O_2S$ [M+H] requires: 423.0156: found: 423.0151.

4.4 NMR Data

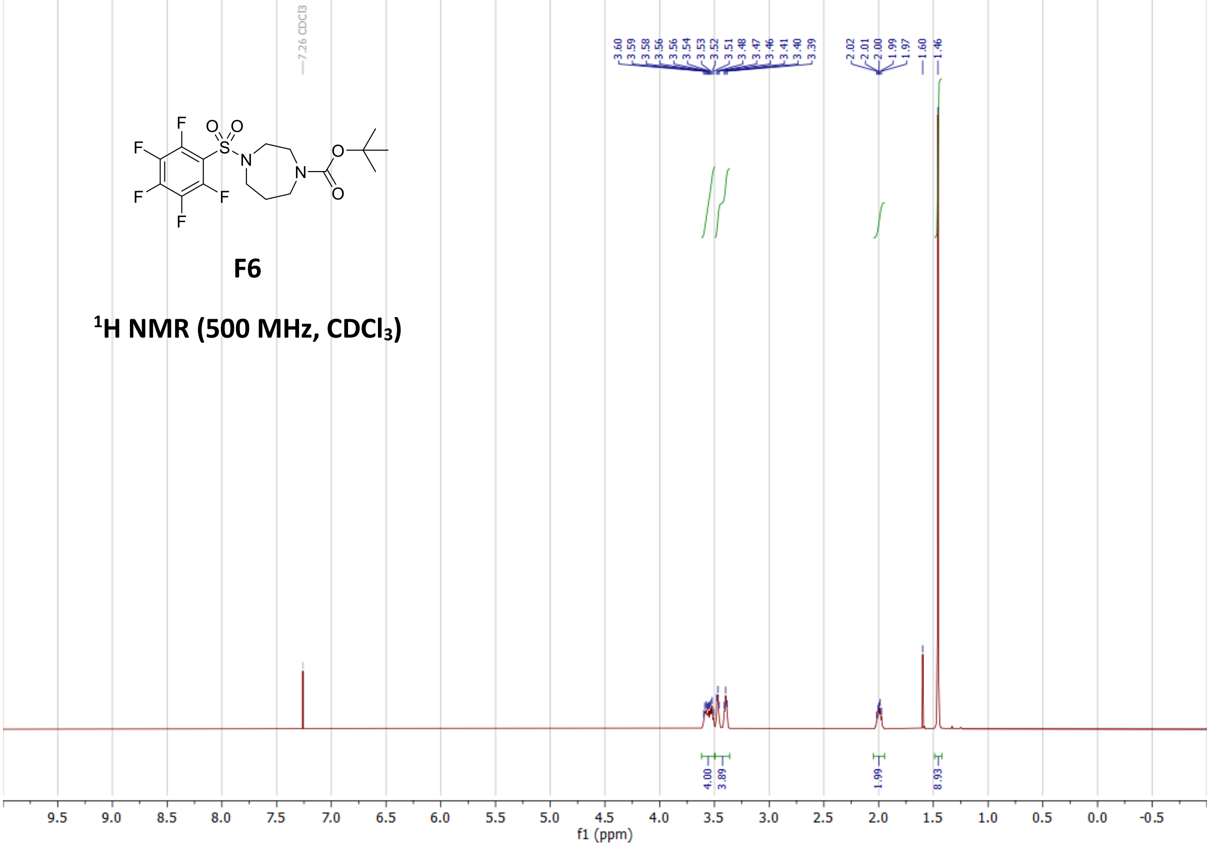

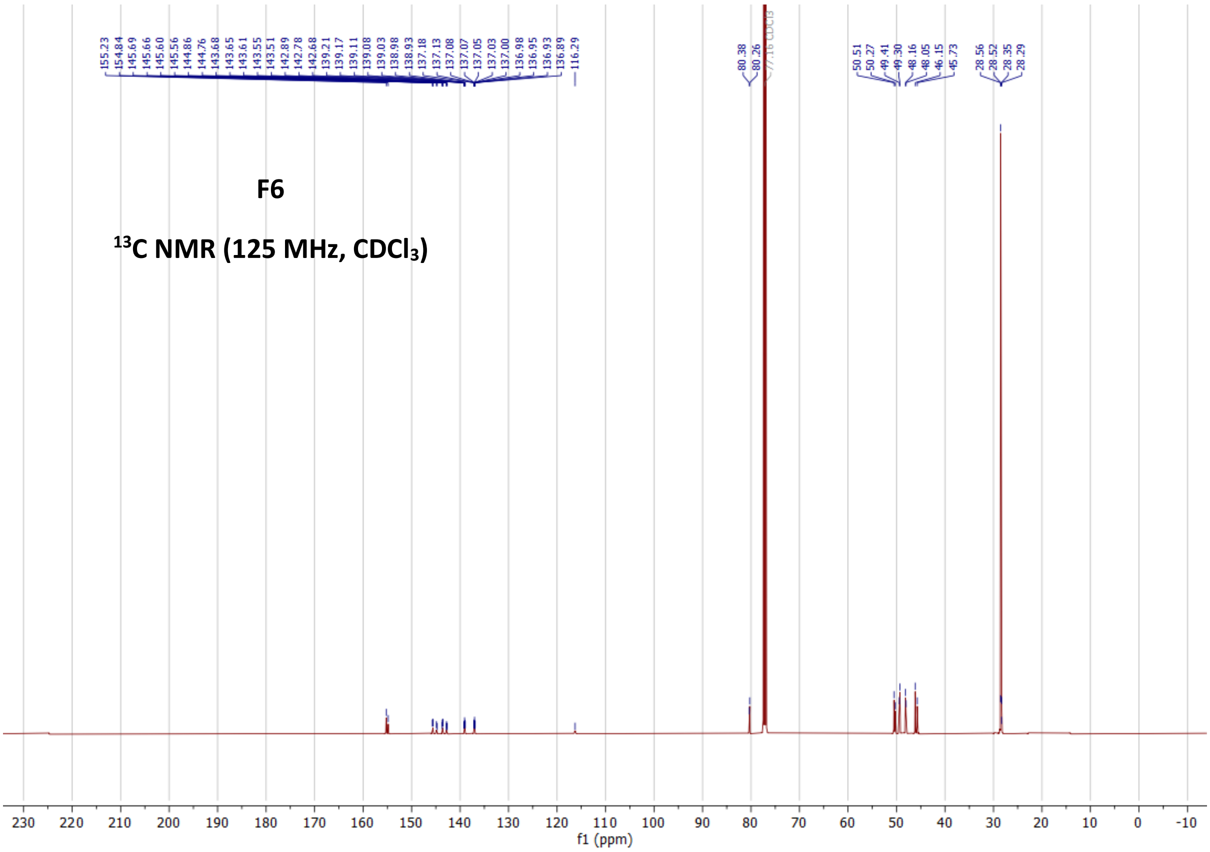

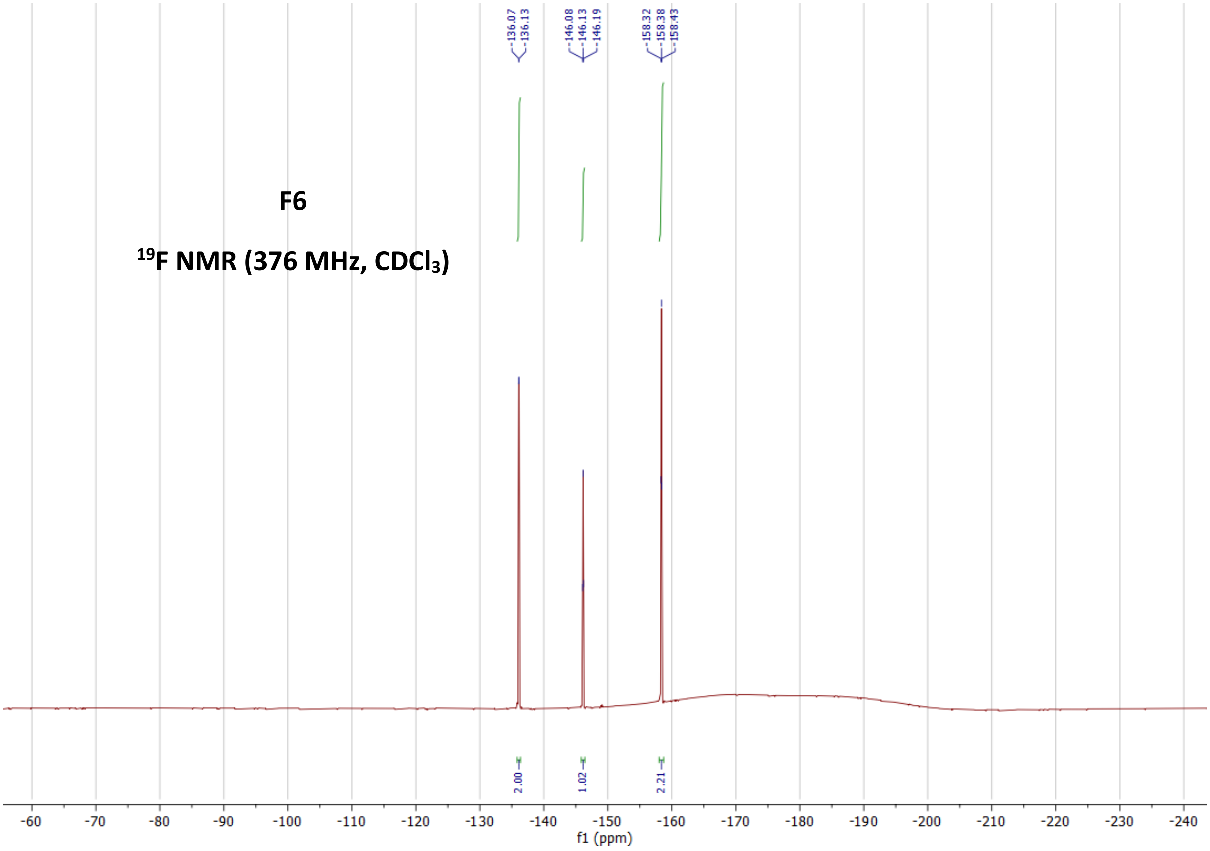
F6
19F NMR (376 MHz, CDCl3)
-136.07
-136.13
-146.08
-146.13
-146.19
-158.32
-158.38
-158.43
2.00
1.02
2.21
-60
-70
-80
-90
-100
-110
-120
-130
-140
-150
-160
-170
-180
-190
-200
-210
-220
-230
-240
f1 (ppm)

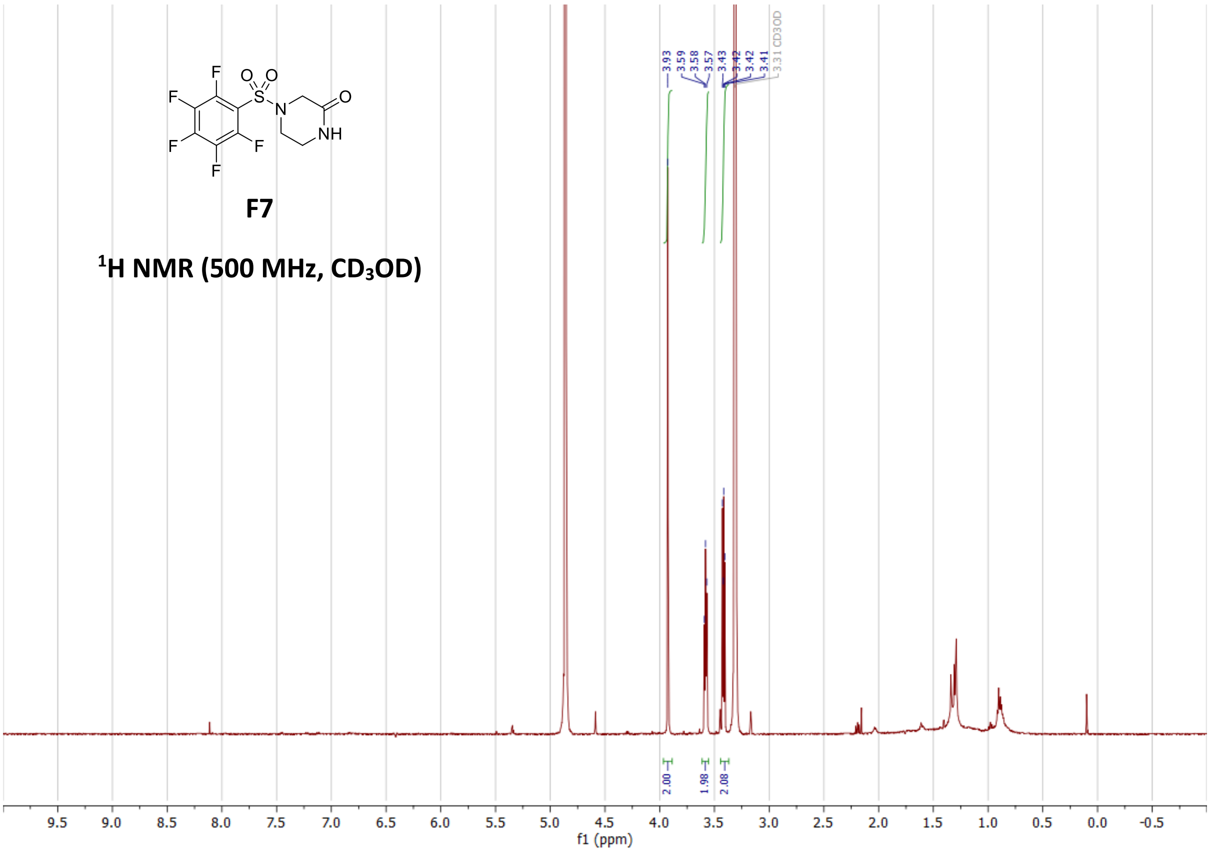
F7
1H NMR (500 MHz, CD3OD)
3.93
3.59
3.58
3.57
3.43
3.42
3.42
3.41
3.31 CD3OD
2.00
1.98
2.08
f1 (ppm)

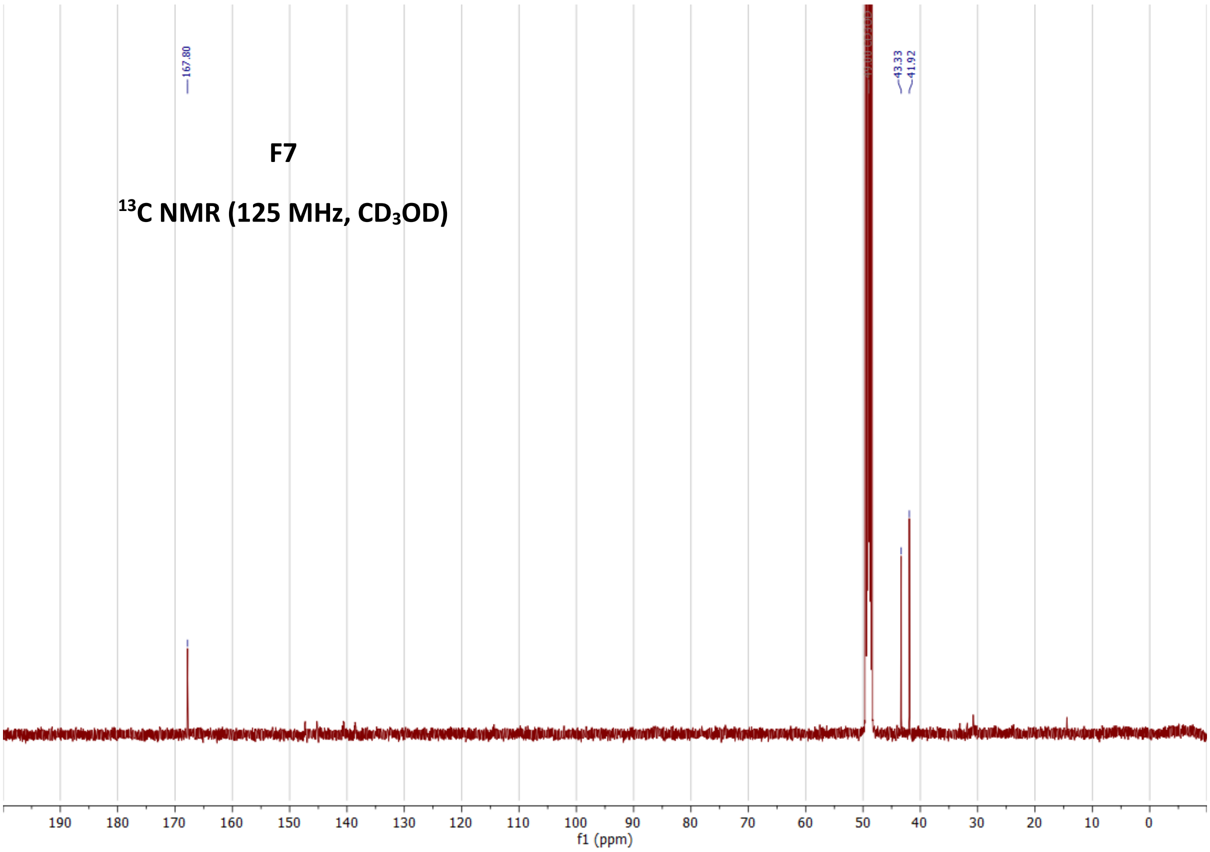
F7
13C NMR (125 MHz, CD3OD)
167.80
43.33
41.92
f1 (ppm)

F7

^{19}F NMR (376 MHz, CD_3OD)

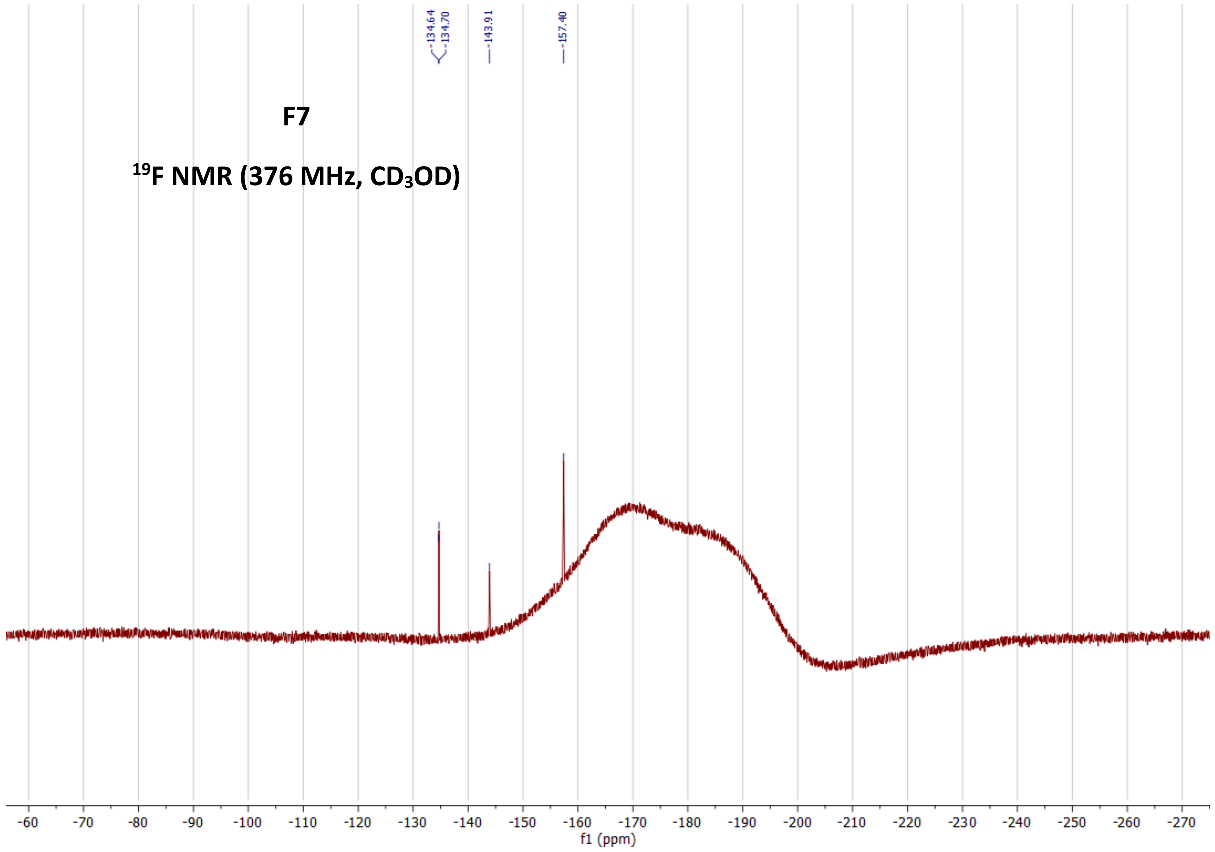

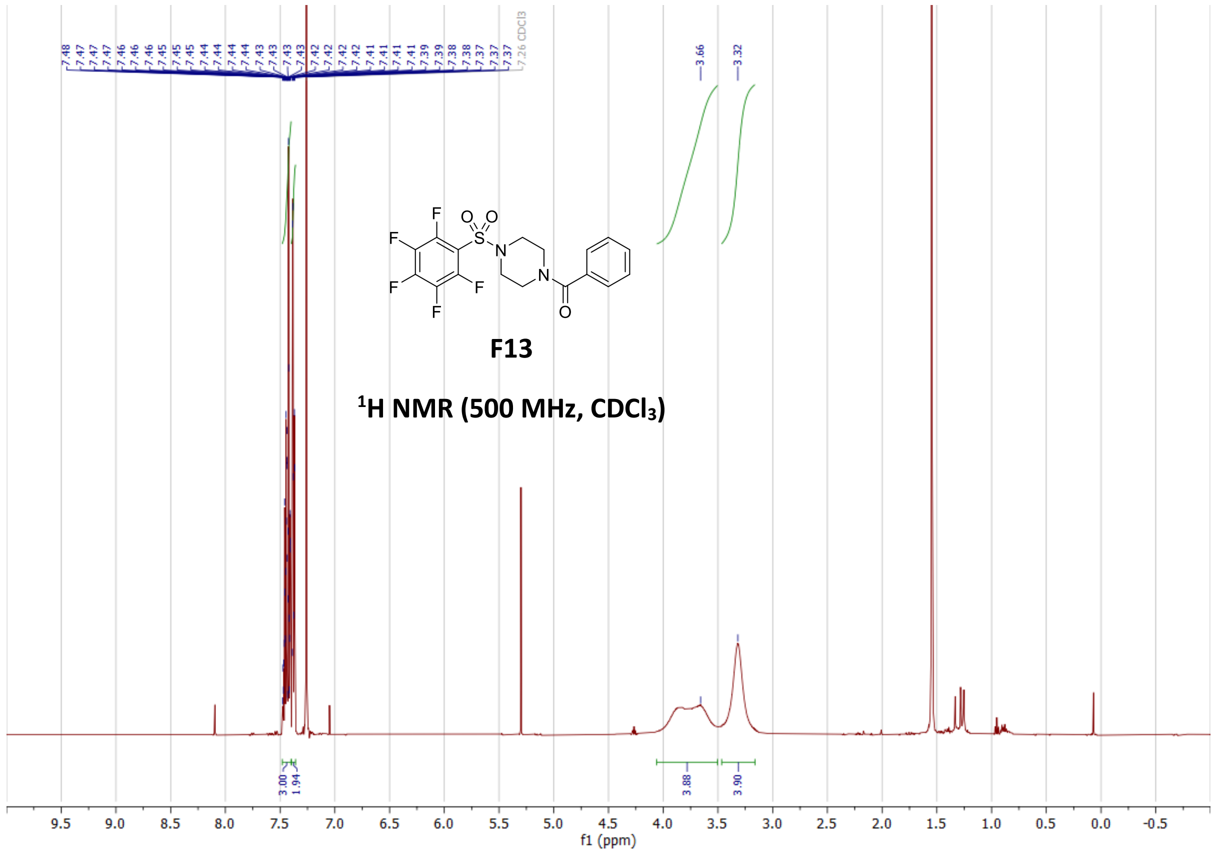
F13
1H NMR (500 MHz, CDCl3)
f1 (ppm)

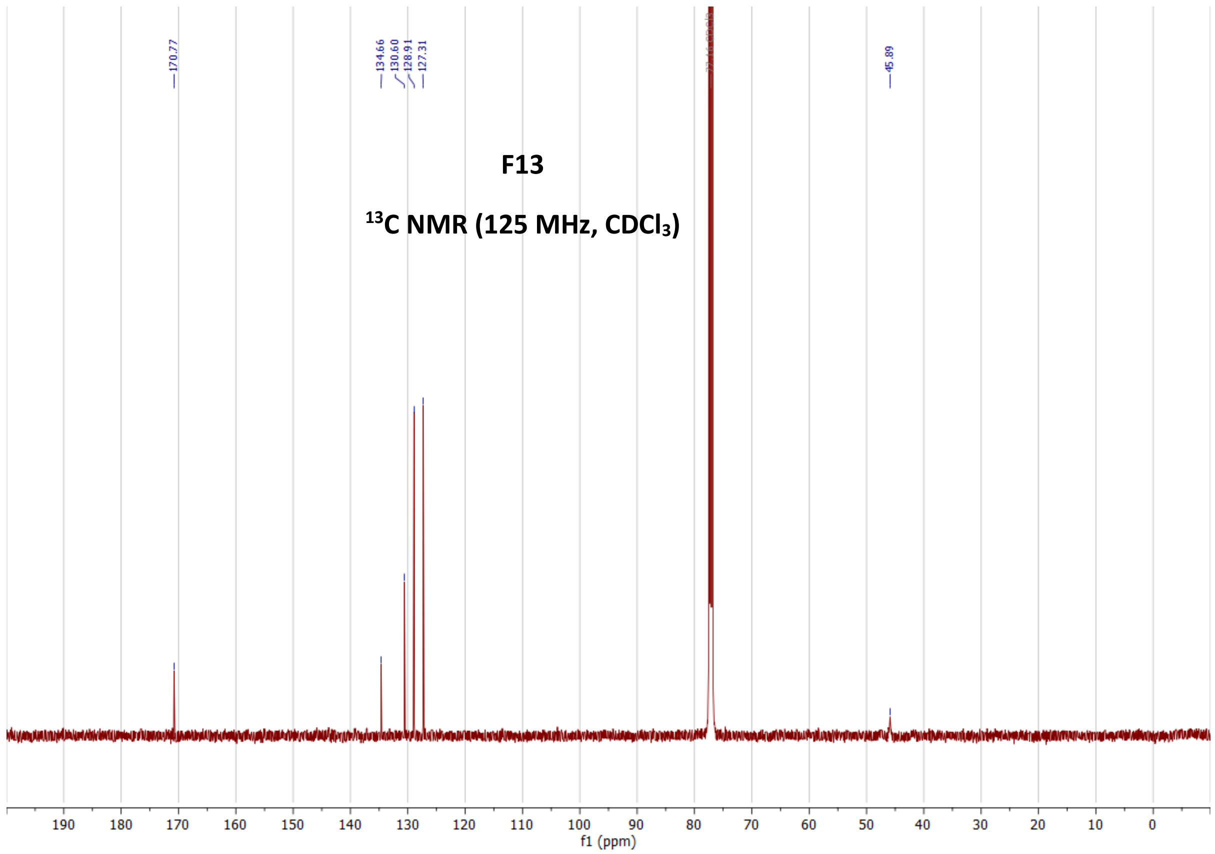
170.77
134.66
130.60
128.91
127.31
45.89
F13
^{13}C NMR (125 MHz, CDCl$_3$)
f1 (ppm)

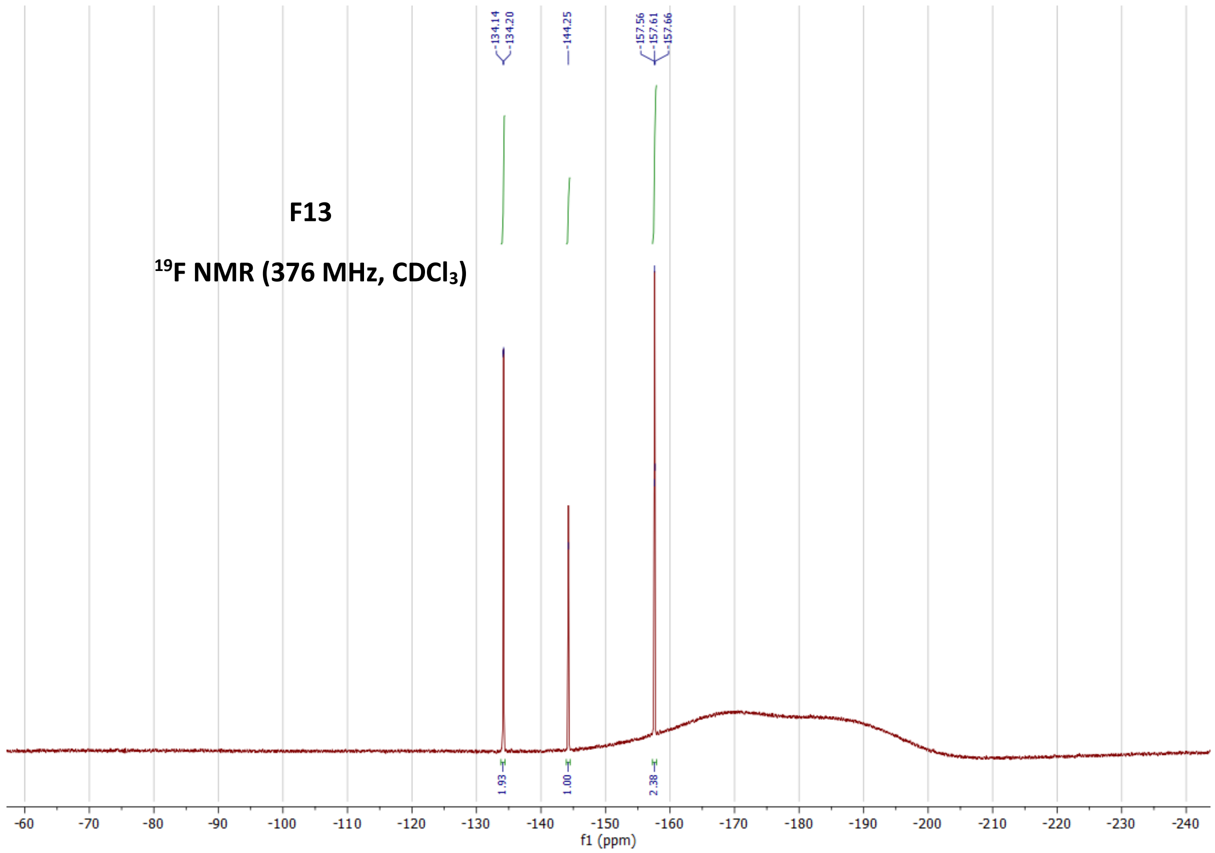
134.14
134.20
144.25
157.56
157.61
157.66
F13
^{19}F NMR (376 MHz, CDCl$_3$)
1.93
1.00
2.38
f1 (ppm)

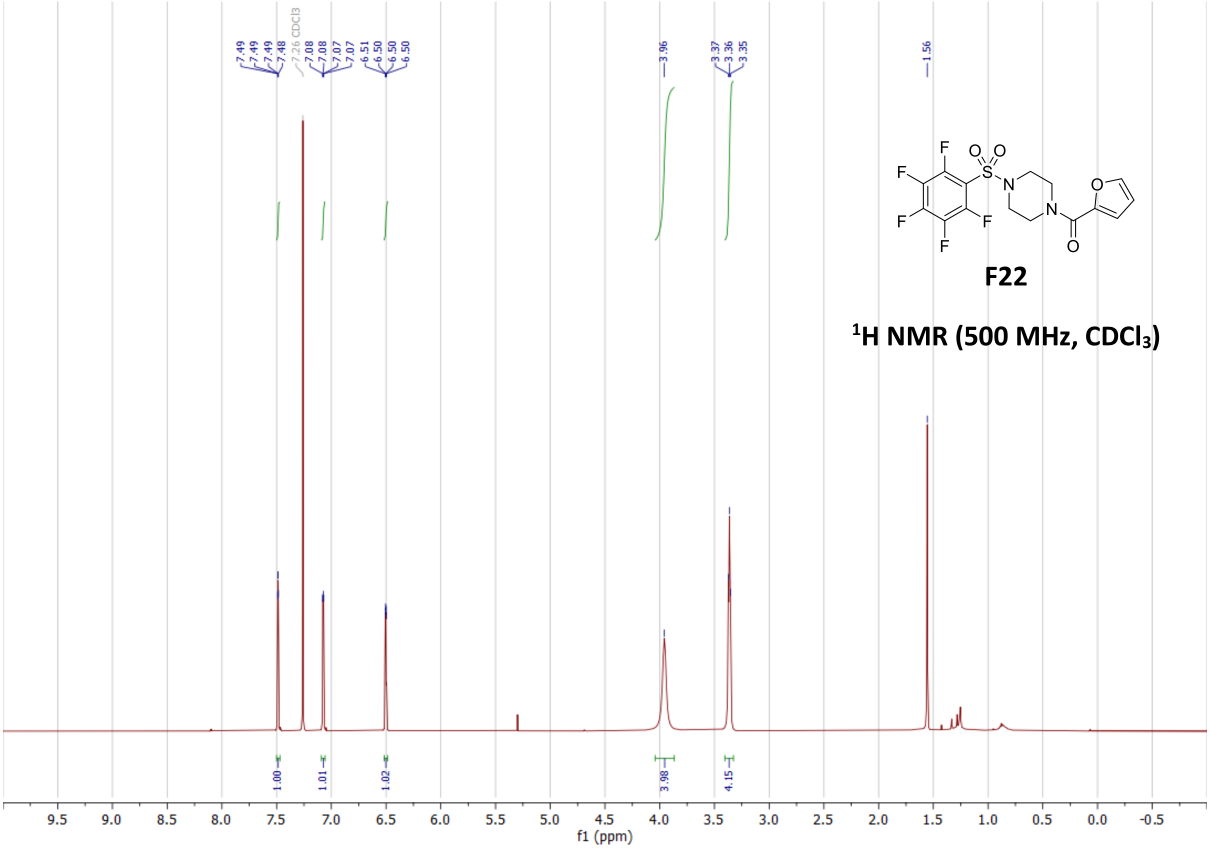

F22

^{1}H NMR (500 MHz, $CDCl_3$)

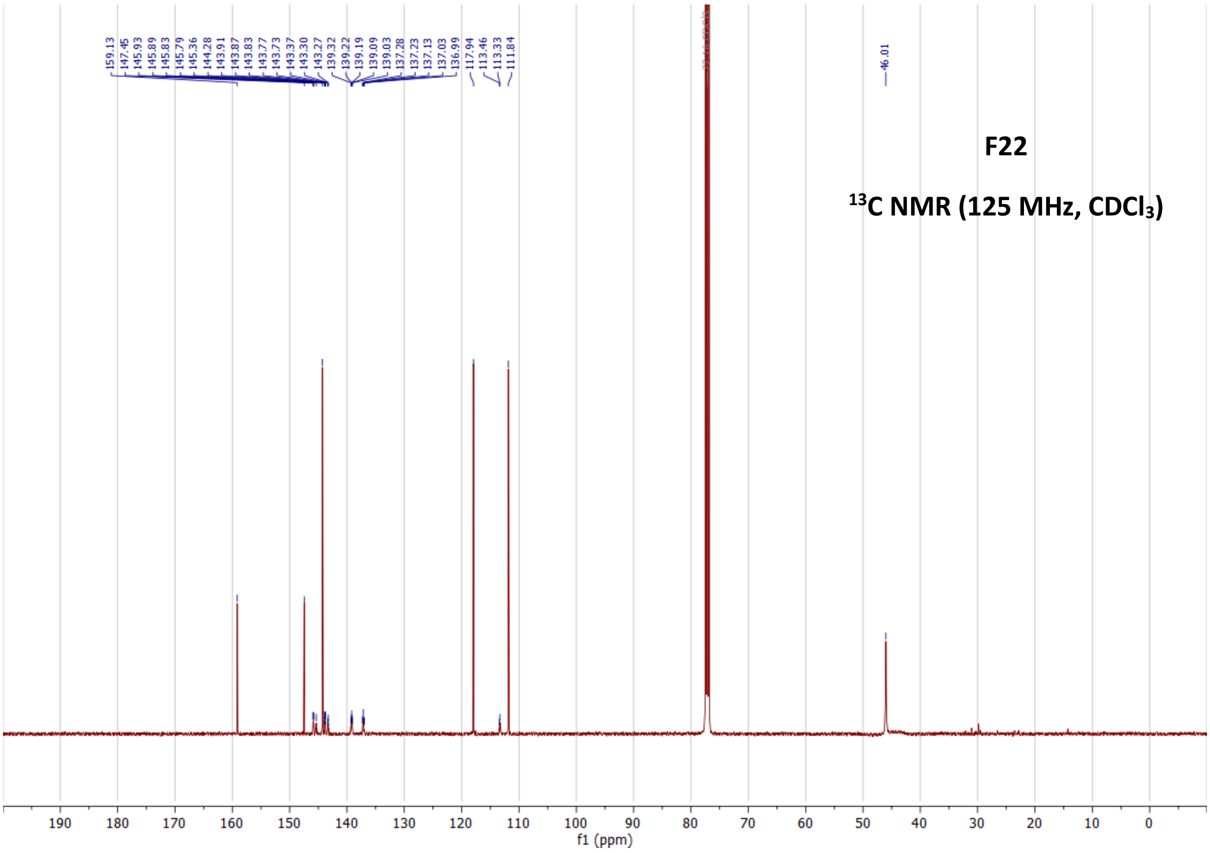

F22

^{13}C NMR (125 MHz, $CDCl_3$)

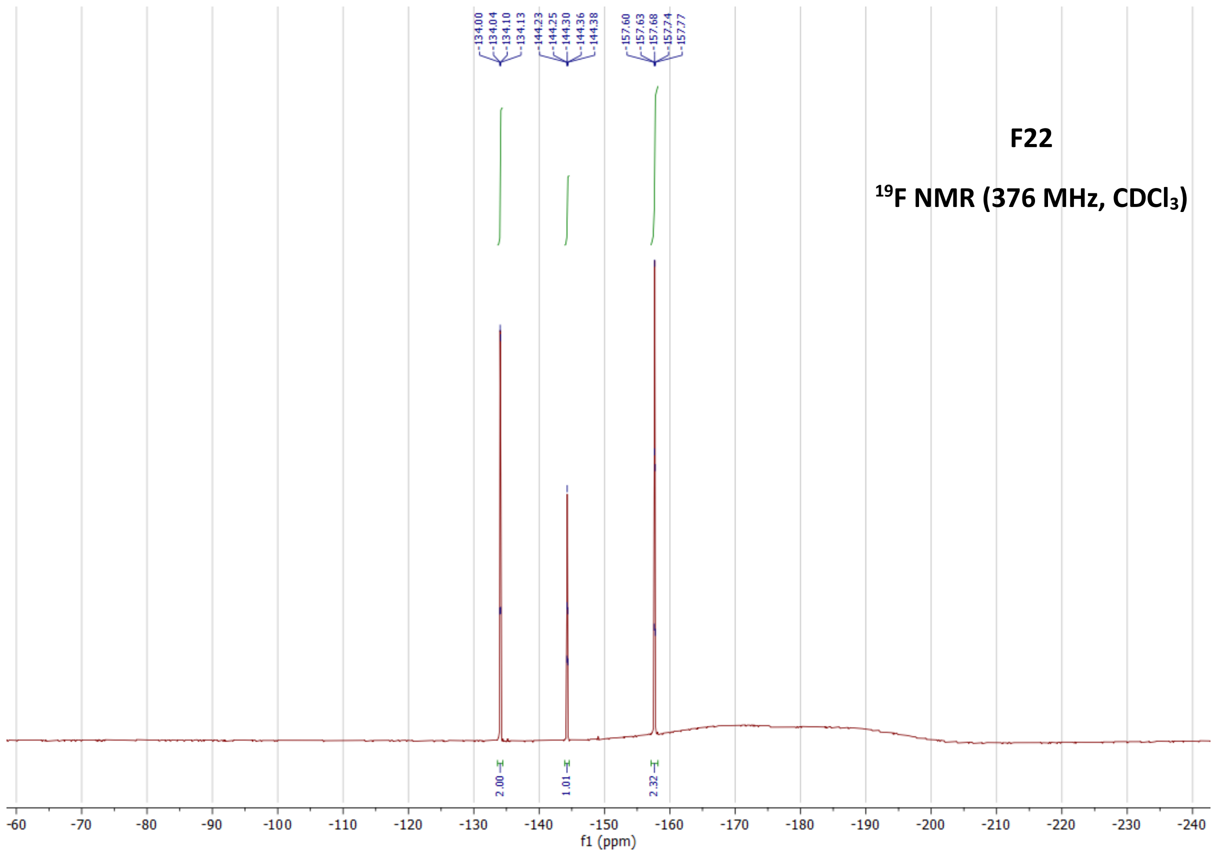
F22
^{19}F NMR (376 MHz, CDCl$_3$)
-134.00
-134.04
-134.10
-134.13
-144.23
-144.25
-144.30
-144.36
-144.38
-157.60
-157.63
-157.68
-157.74
-157.77
2.00
1.01
2.32
-60 -70 -80 -90 -100 -110 -120 -130 -140 -150 -160 -170 -180 -190 -200 -210 -220 -230 -240
f1 (ppm)

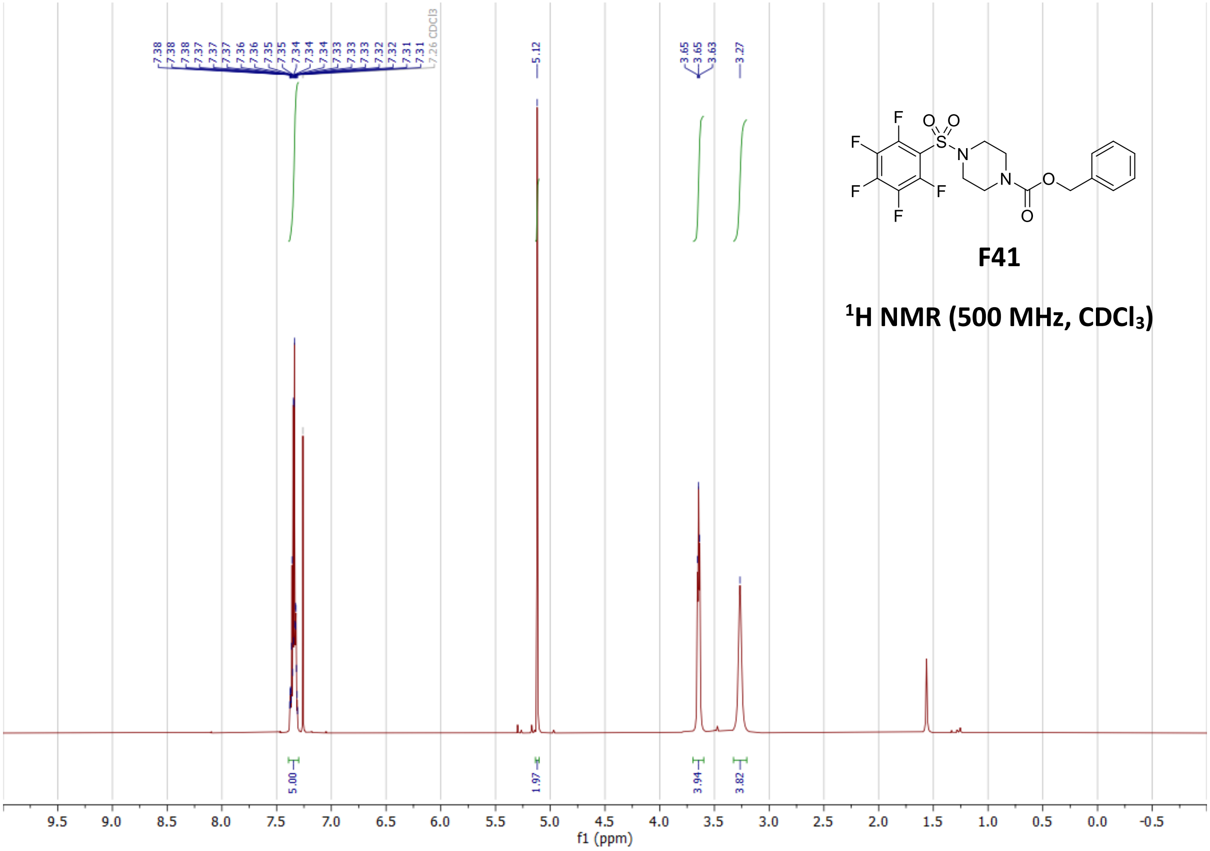
F41
^{1}H NMR (500 MHz, CDCl$_3$)
7.38 7.38 7.38 7.37 7.37 7.37 7.36 7.36 7.35 7.35 7.34 7.34 7.34 7.34 7.33 7.33 7.33 7.33 7.32 7.32 7.31 7.31
7.26 CDCl3
5.12
3.65 3.65 3.63
3.27
5.00
1.97
3.94
3.82
f1 (ppm)

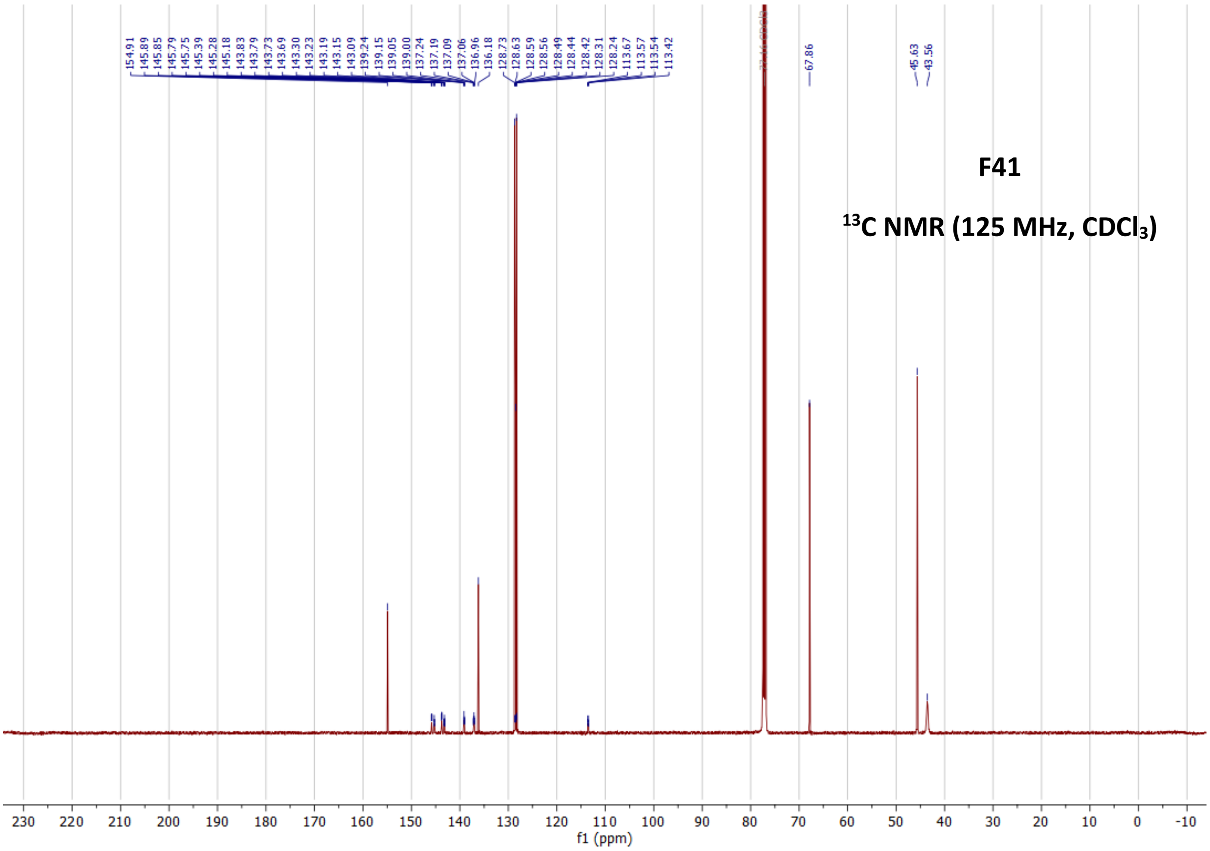
F41
^{13}C NMR (125 MHz, CDCl$_3$)
154.91 145.89 145.85 145.79 145.75 145.39 145.28 145.18 143.83 143.79 143.73 143.69 143.30 143.23 143.19 143.15 143.09 139.24 139.15 139.05 139.00 137.24 137.19 137.09 137.06 136.96 136.18 128.73 128.63 128.59 128.56 128.49 128.44 128.42 128.31 128.24 113.67 113.57 113.54 113.42
67.86
45.63
43.56
f1 (ppm)

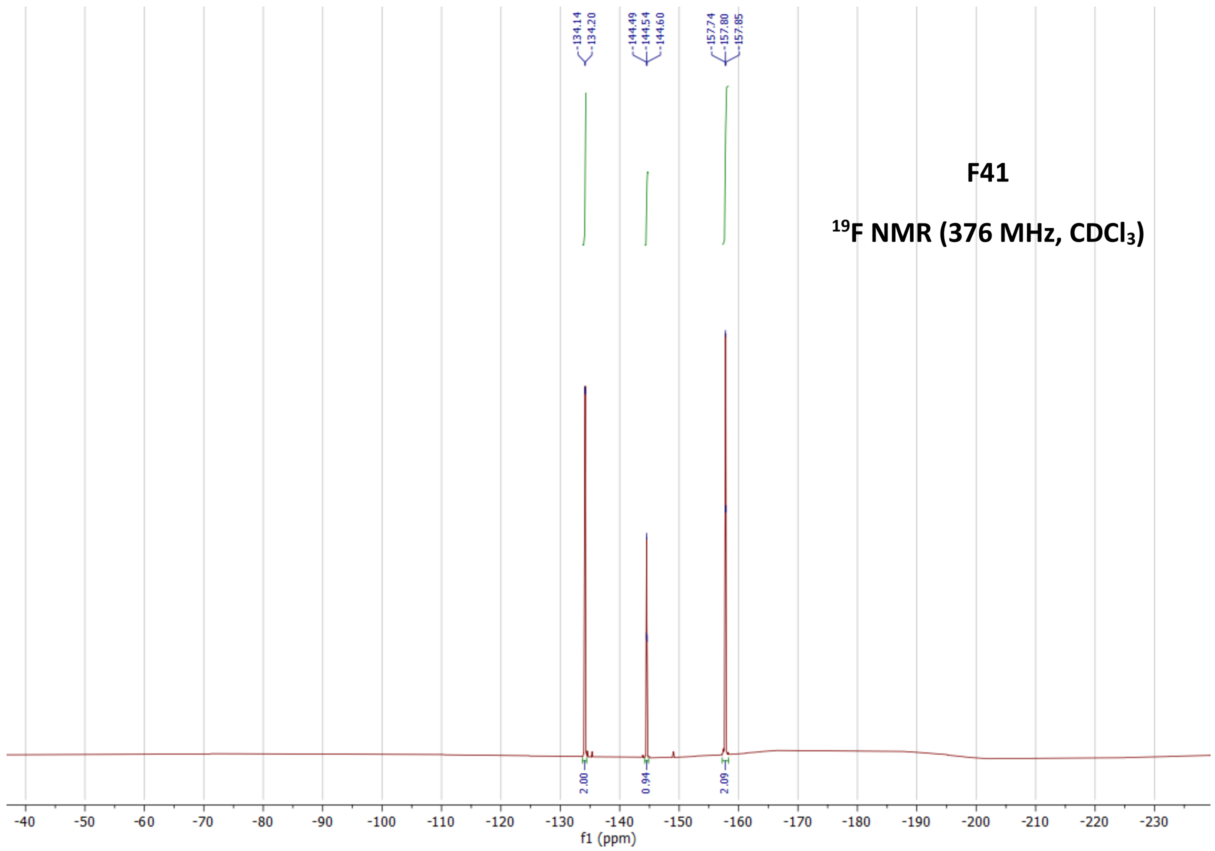

-134.14
-134.20
-144.49
-144.54
-144.60
-157.74
-157.80
-157.85
F41
^{19}F NMR (376 MHz, CDCl$_3$)
2.00
0.94
2.09
-40
-50
-60
-70
-80
-90
-100
-110
-120
-130
-140
-150
-160
-170
-180
-190
-200
-210
-220
-230
f1 (ppm)

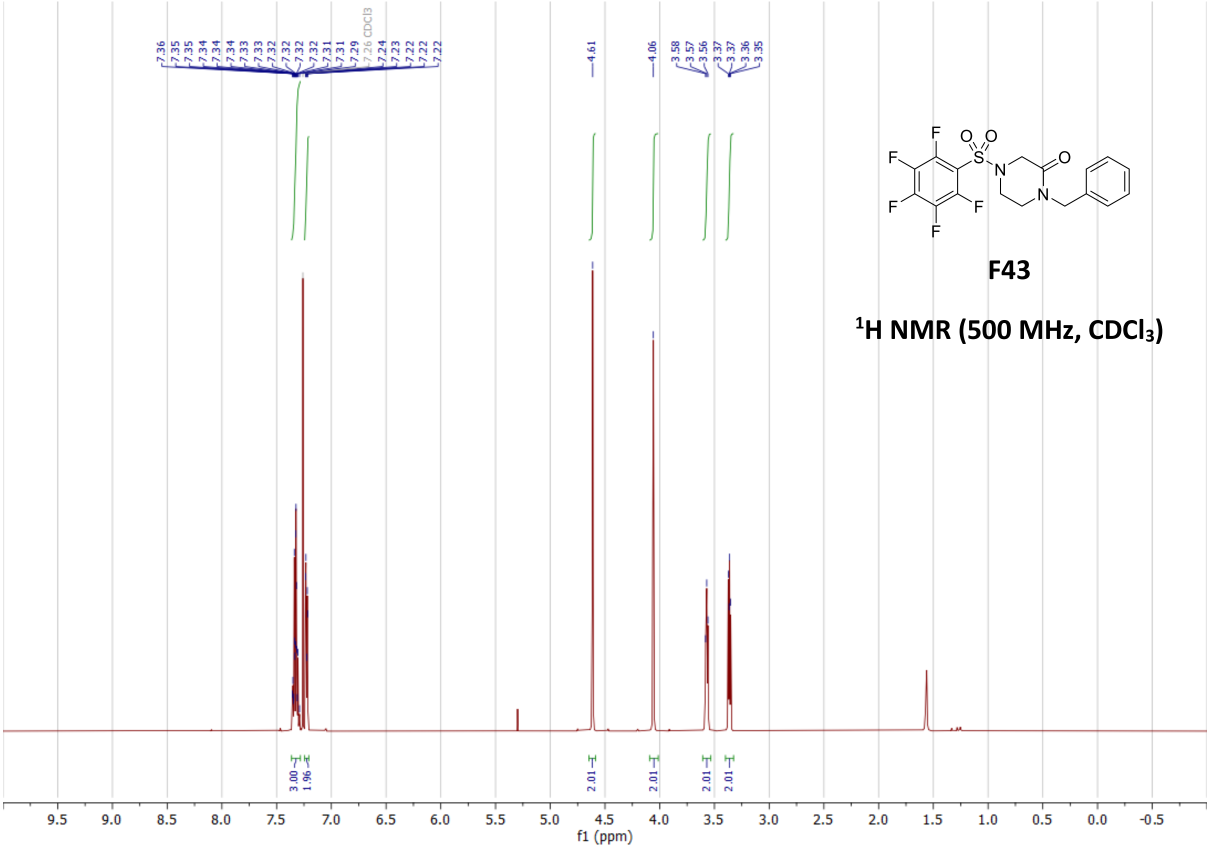

F43

^{1}H NMR (500 MHz, $CDCl_3$)

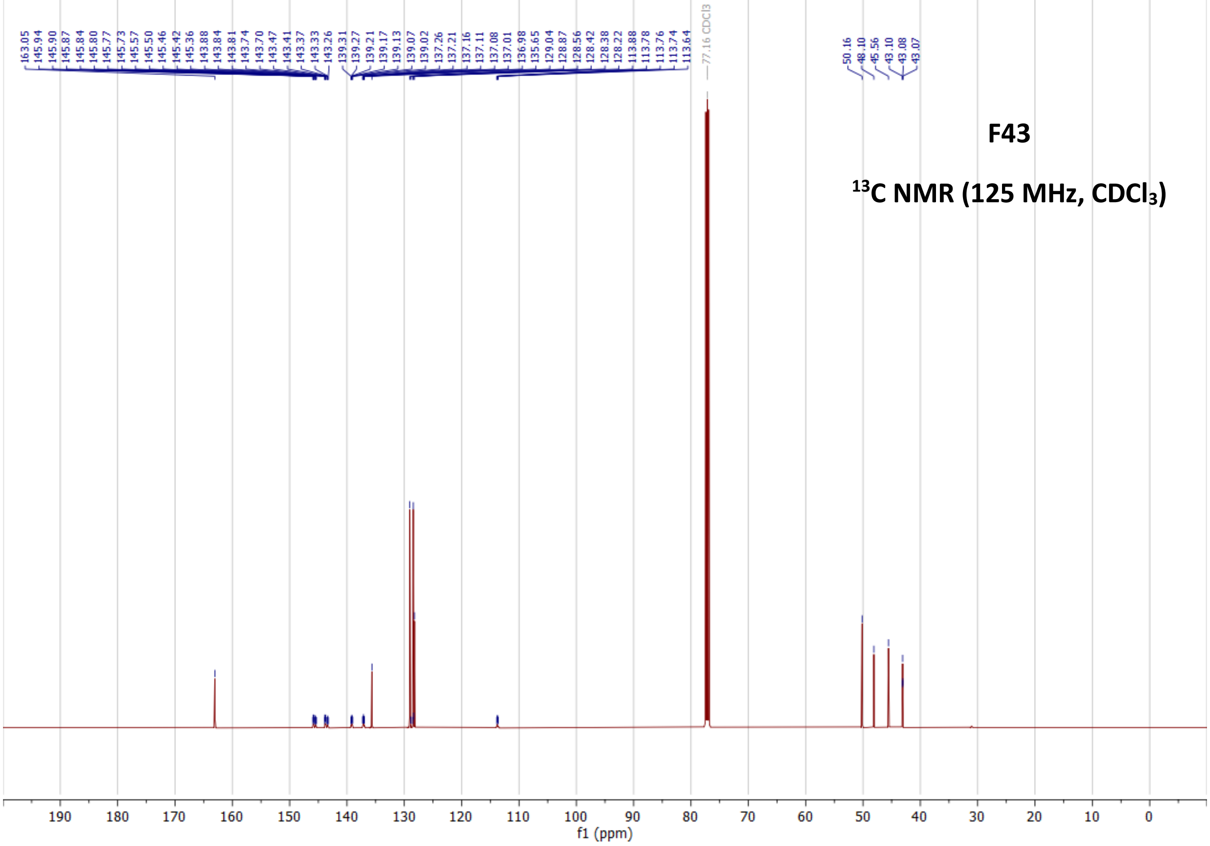

F43

^{13}C NMR (125 MHz, $CDCl_3$)

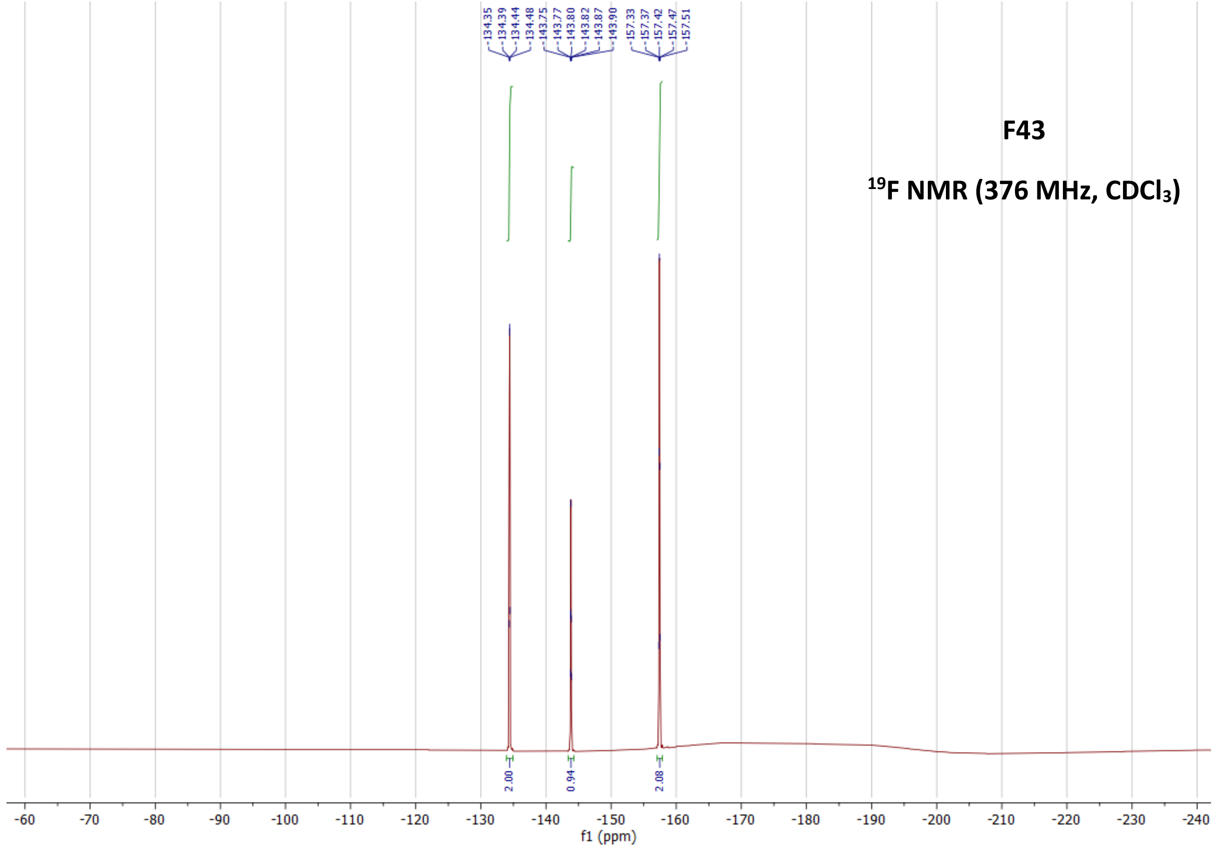

-134.35
-134.39
-134.44
-134.48
-143.75
-143.77
-143.80
-143.82
-143.87
-143.90
-157.33
-157.37
-157.42
-157.47
-157.51
F43
^{19}F NMR (376 MHz, CDCl$_3$)
2.00
0.94
2.08
-60 -70 -80 -90 -100 -110 -120 -130 -140 -150 -160 -170 -180 -190 -200 -210 -220 -230 -240
f1 (ppm)

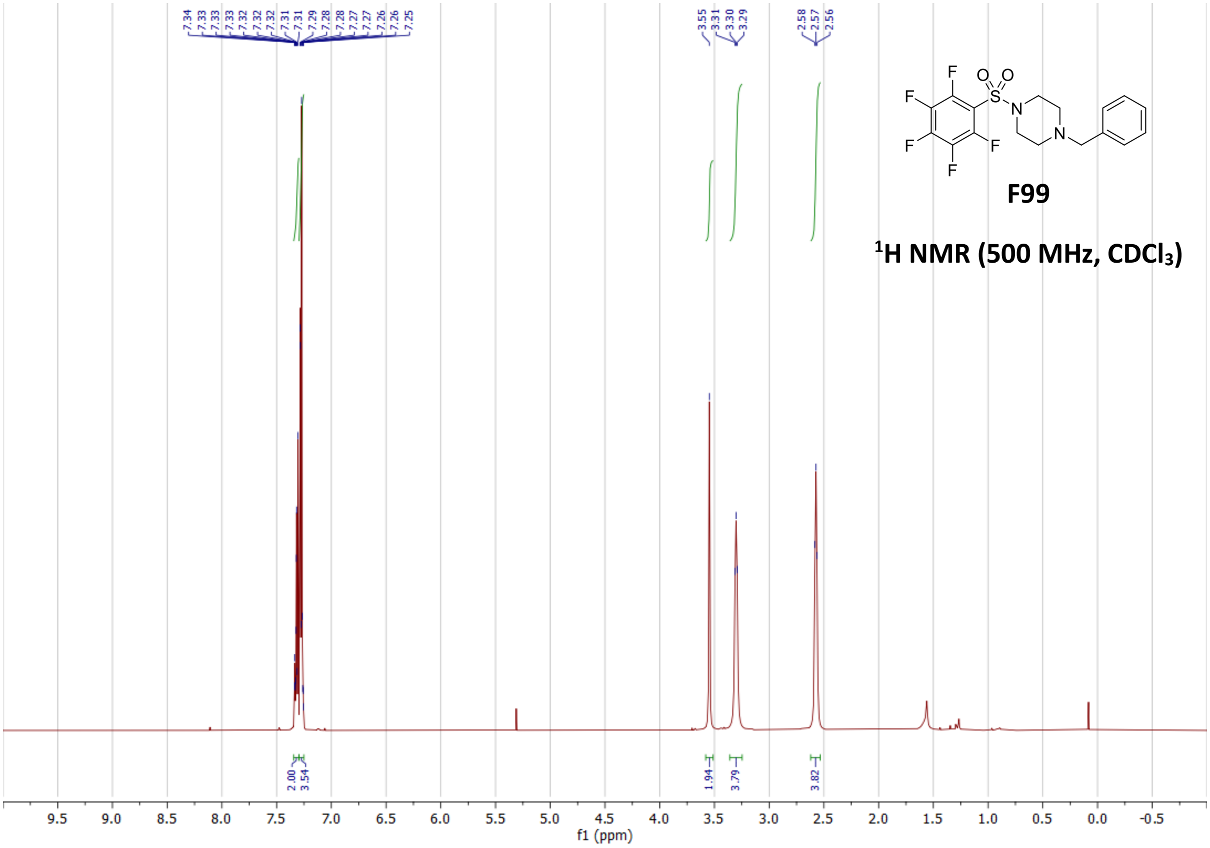

F99

^{1}H NMR (500 MHz, CDCl$_3$)

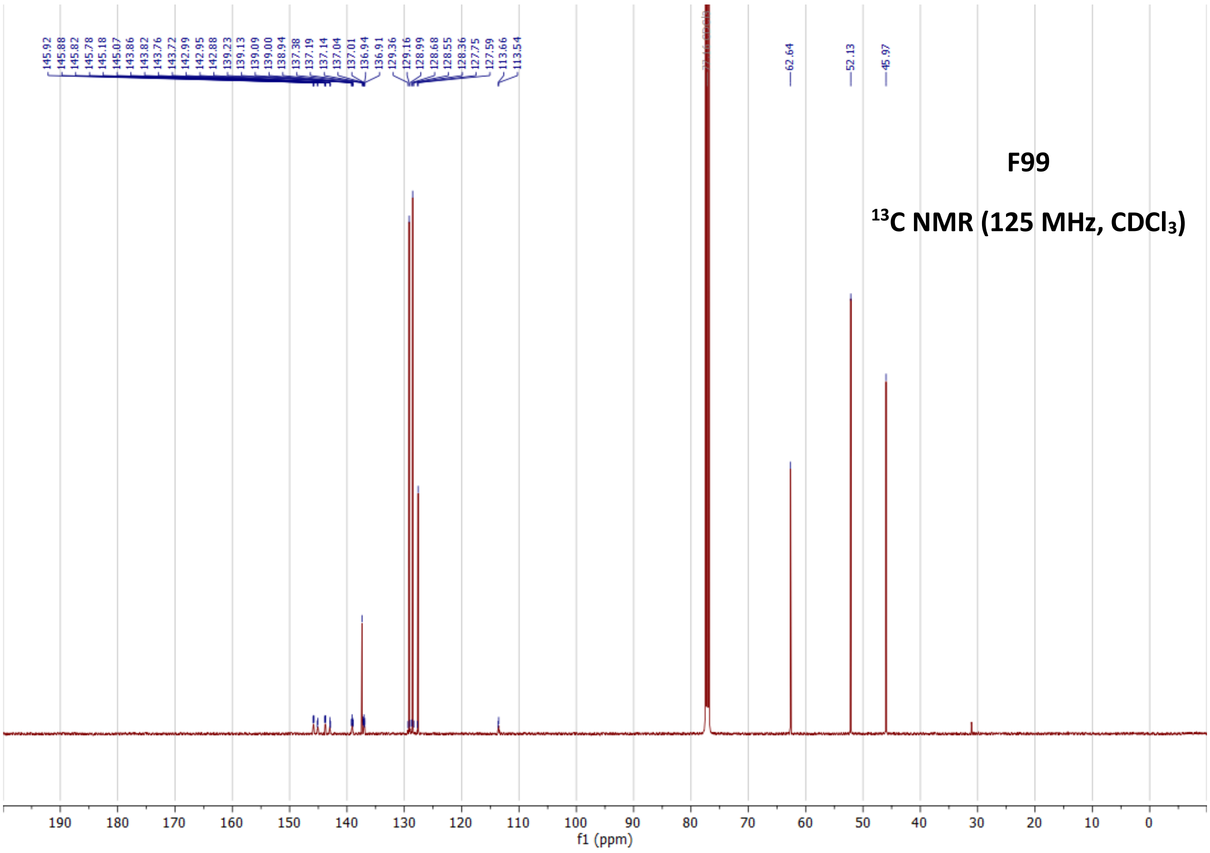

F99

^{13}C NMR (125 MHz, CDCl$_3$)

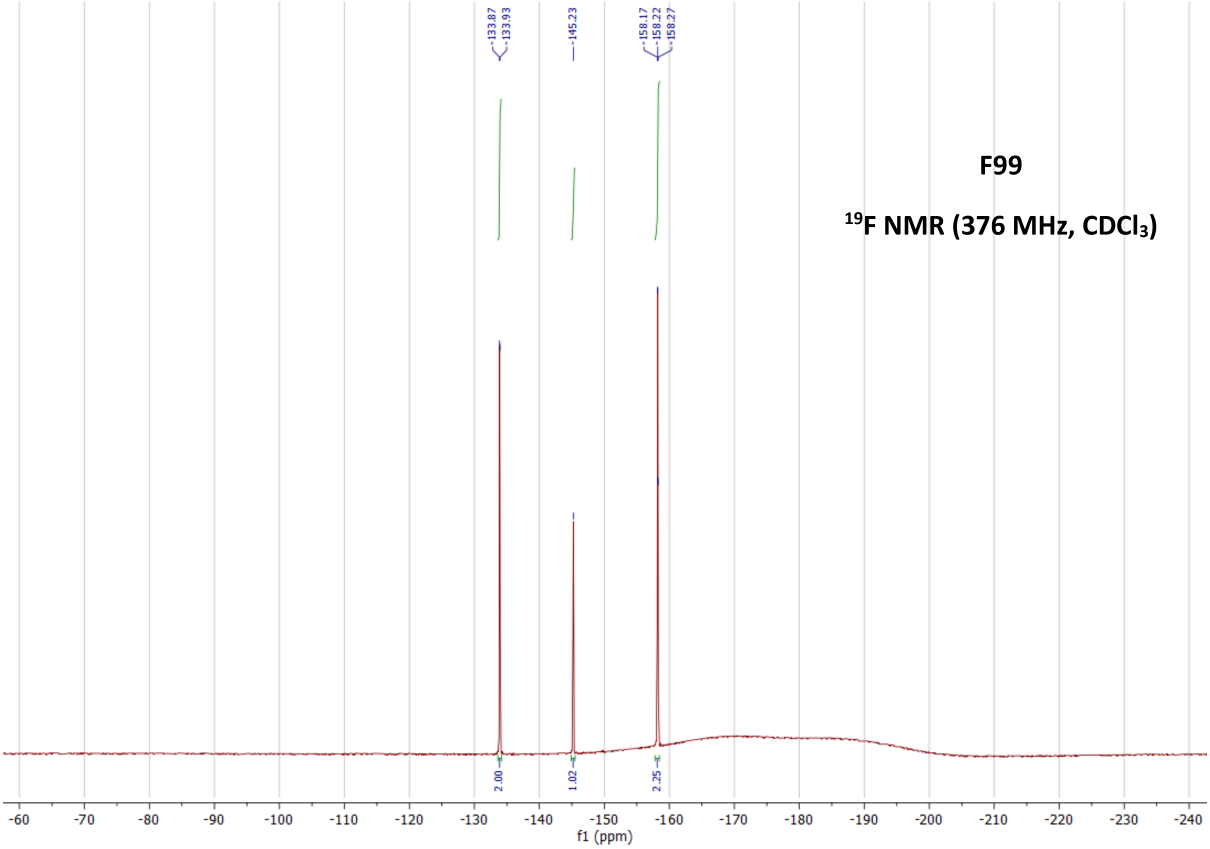

F99

^{19}F NMR (376 MHz, $CDCl_3$)

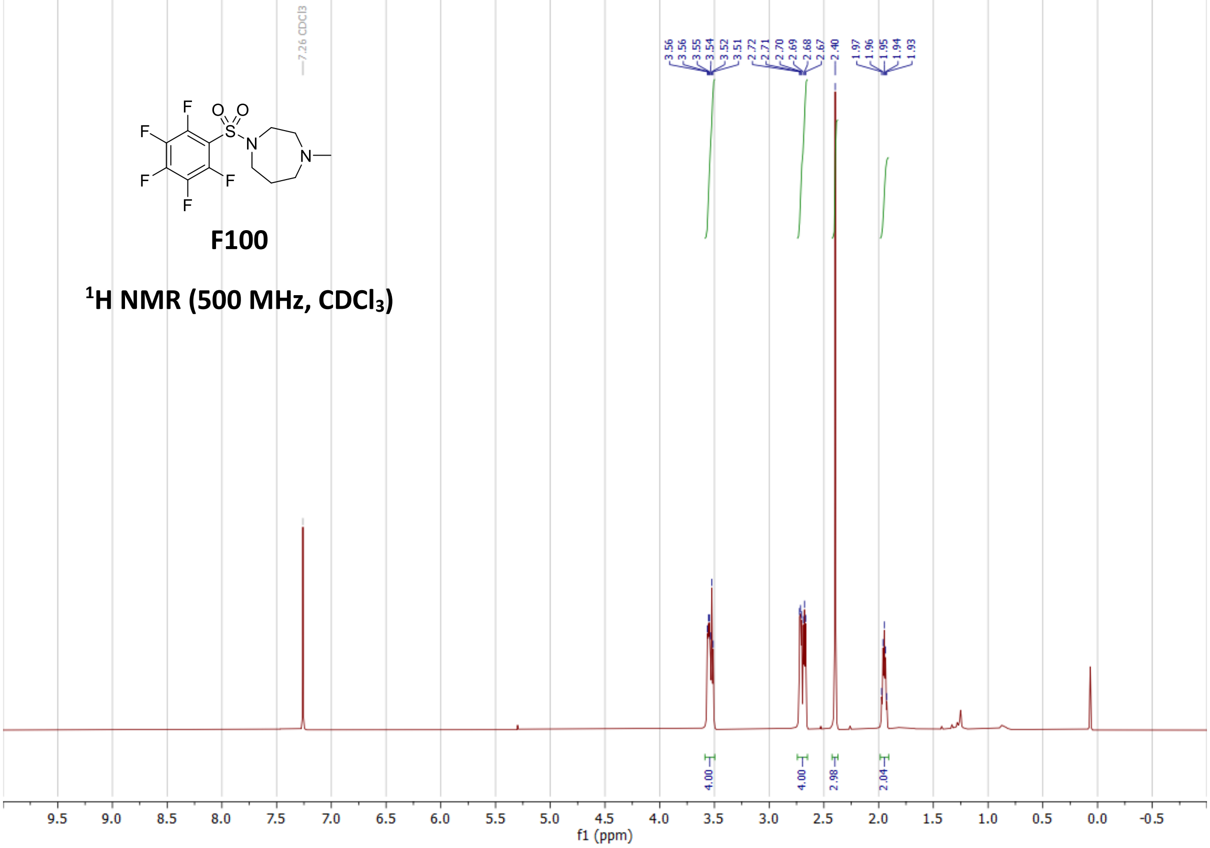

F100

^{1}H NMR (500 MHz, $CDCl_3$)

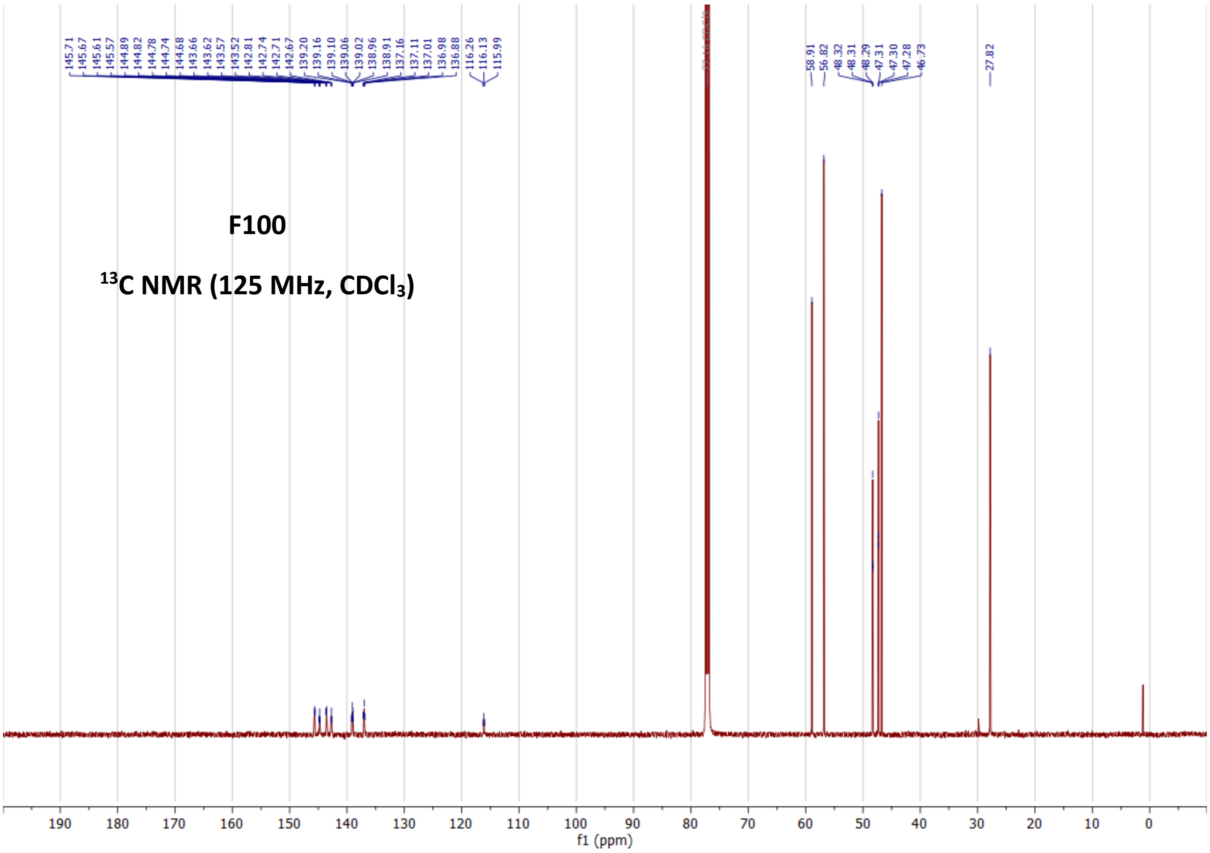

F100

^{13}C NMR (125 MHz, $CDCl_3$)

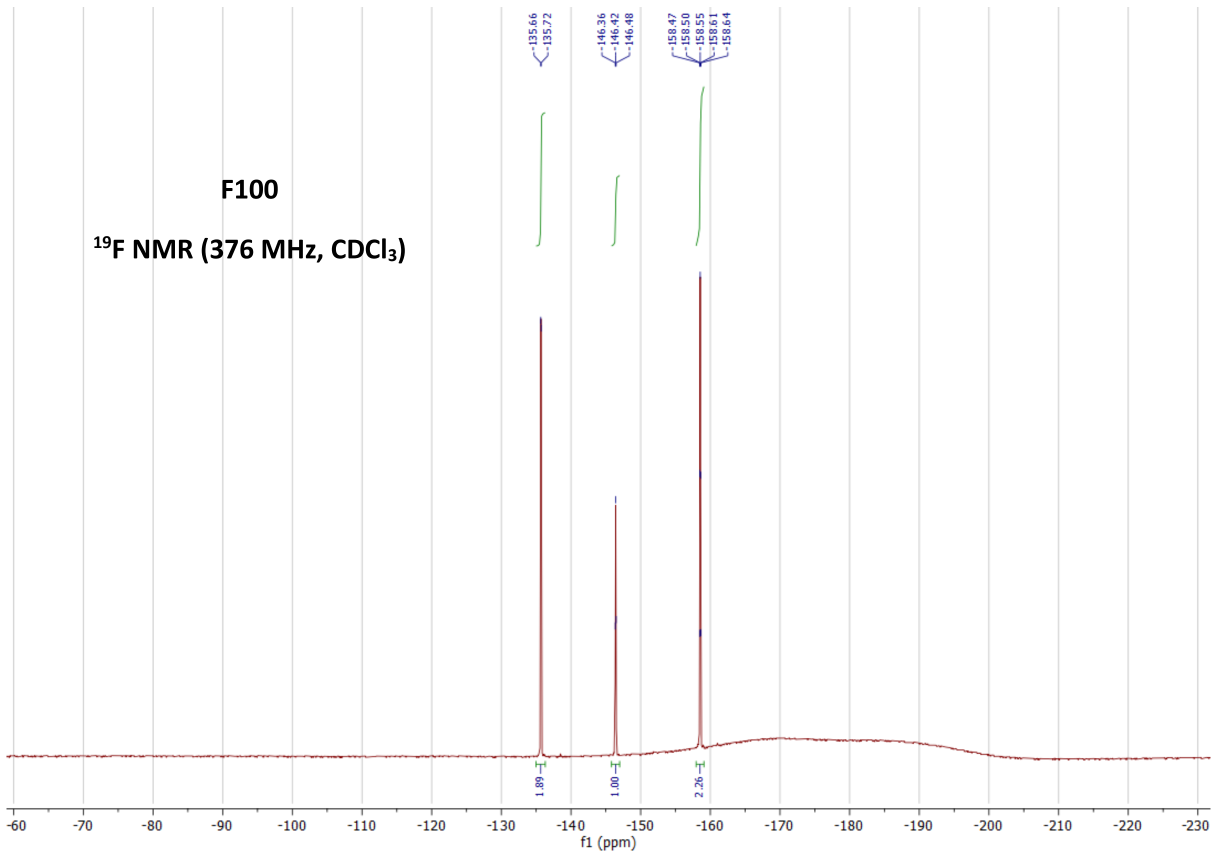
F100
^{19}F NMR (376 MHz, CDCl$_3$)
-135.66
-135.72
-146.36
-146.42
-146.48
-158.47
-158.50
-158.55
-158.61
-158.64
1.89
1.00
2.26
-60 -70 -80 -90 -100 -110 -120 -130 -140 -150 -160 -170 -180 -190 -200 -210 -220 -230
f1 (ppm)

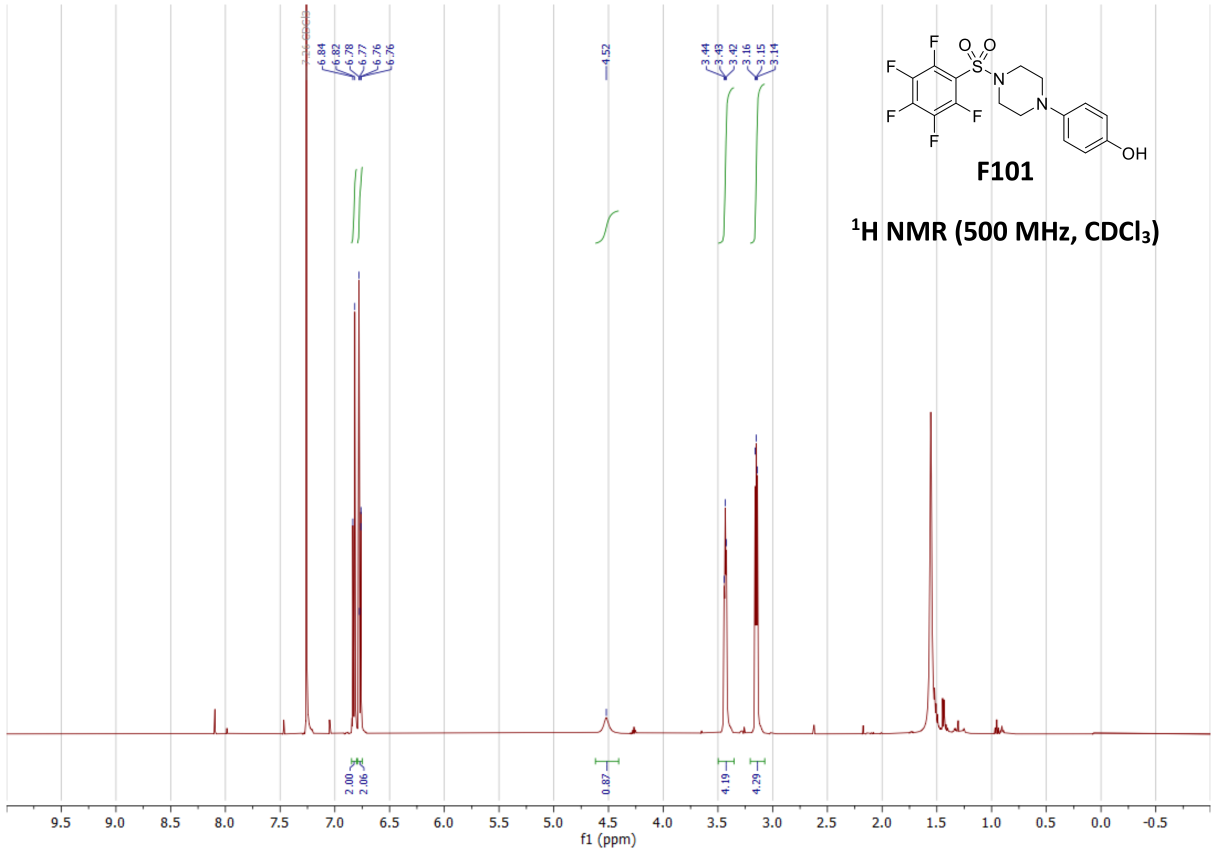
F101
¹H NMR (500 MHz, CDCl₃)
f1 (ppm)

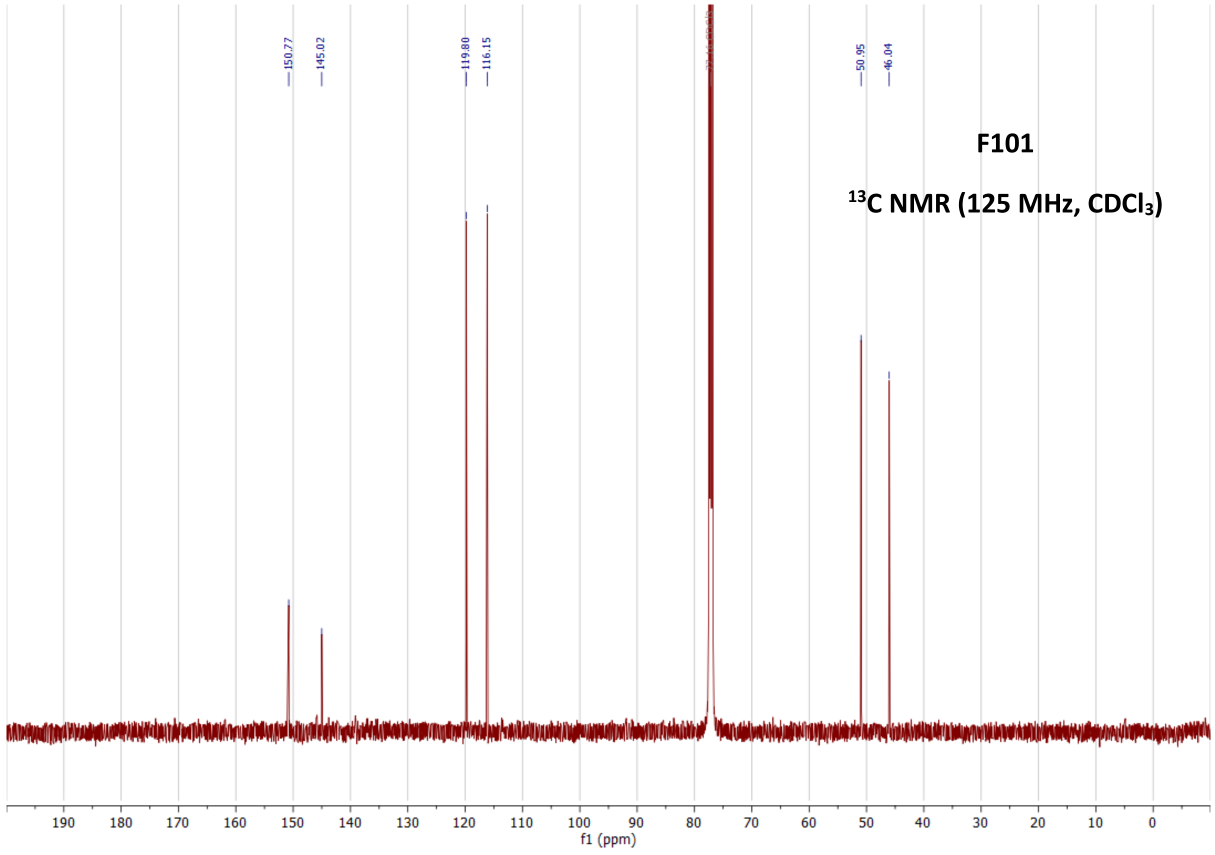
F101
¹³C NMR (125 MHz, CDCl₃)
f1 (ppm)

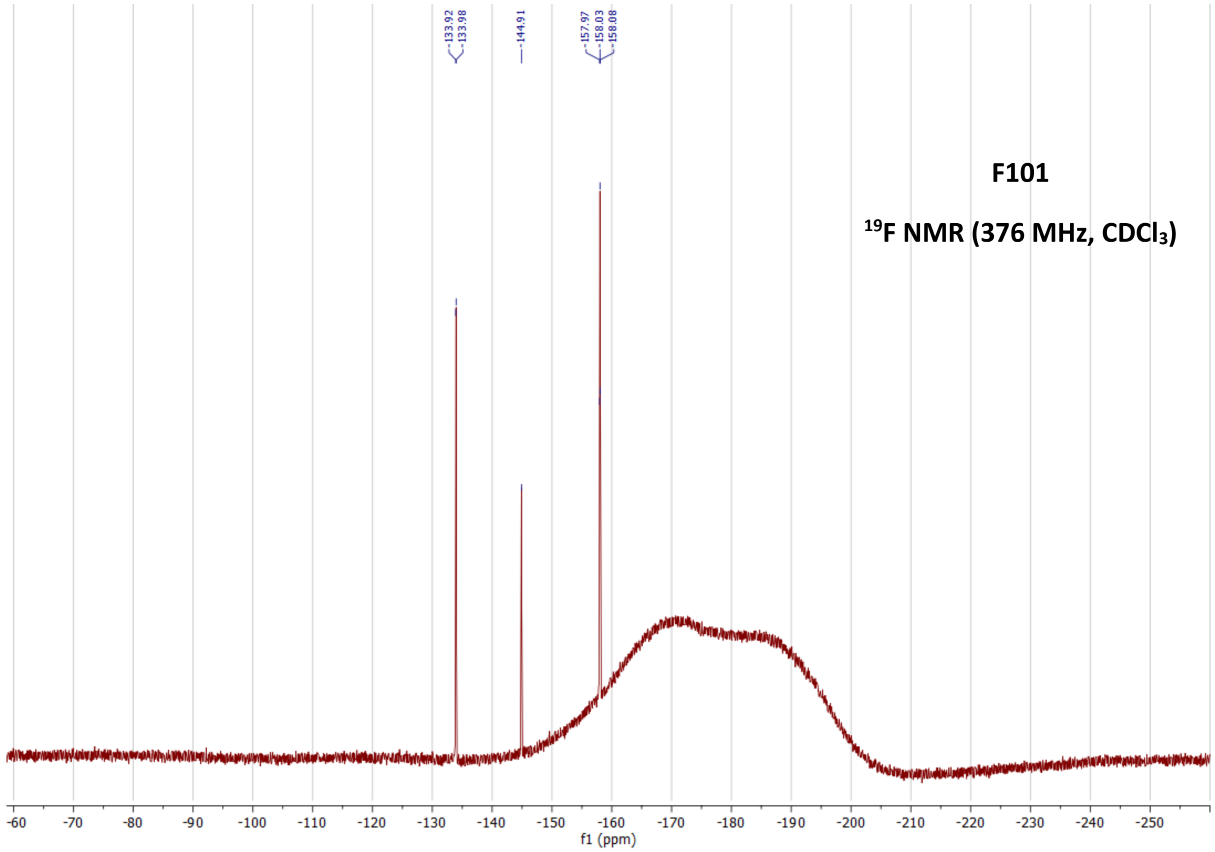

F101

^{19}F NMR (376 MHz, $CDCl_3$)

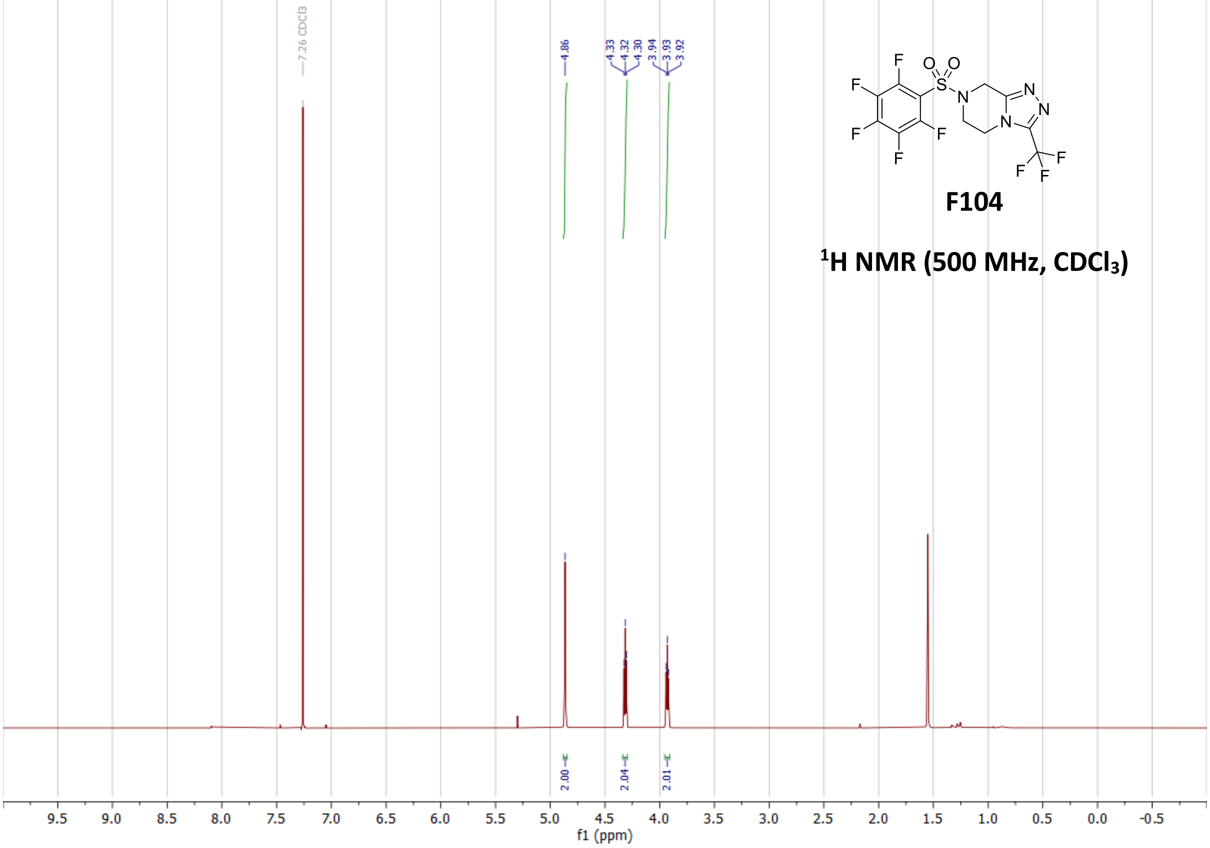

F104

^{1}H NMR (500 MHz, $CDCl_3$)

F104

^{13}C NMR (125 MHz, CDCl$_3$)

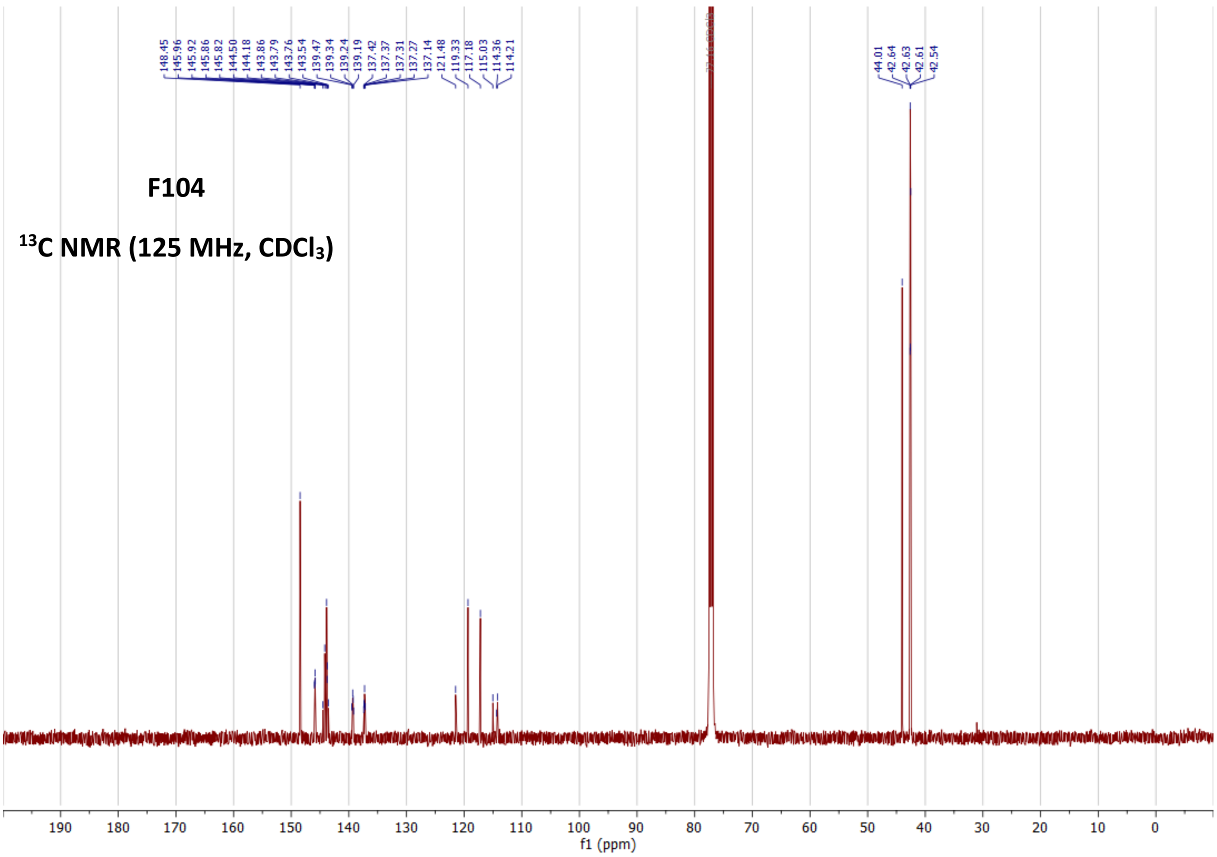

F104

^{19}F NMR (376 MHz, CDCl$_3$)

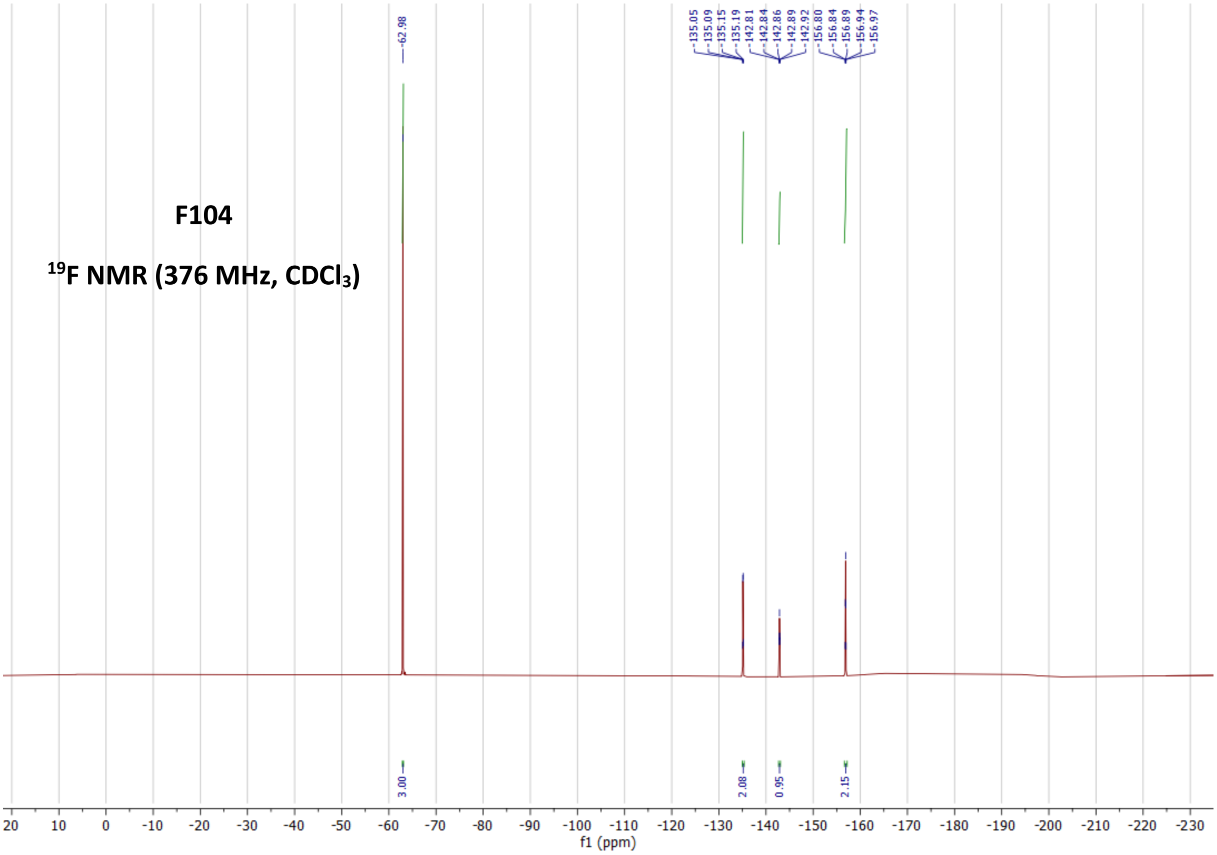

5 References

1. P. D. Mace, K. Linke, R. Feltham, F. R. Schumacher, C. A. Smith, D. L. Vaux, J. Silke and C. L. Day, *J. Biol. Chem*., **2008**, 283, 31633-31640.

2. R. Feltham, B. Bettjeman, R. Budhidarmo, P. D. Mace, S. Shirley, S. M. Condon, S. K. Chunduru, M. A. McKinlay, D. L. Vaux, J. Silke and C. L. Day*, J. Biol. Chem*., **2011**, 286, 17015-17028.

3. J. D. Wright, P. D. Mace and C. L. Day, *Nat. Struct. Mol. Biol.,* **2016**, 23, 45-52.

4. G. Winter, *J. Appl. Crystallogr.*, **2010**, *43*, 186-190.

5. G. Winter, D. G. Waterman, J. M. Parkhurst, A. S. Brewster, R. J. Gildea, M. Gerstel, L. Fuentes-Montero, M. Vollmar, T. Michels-Clark, I. D. Young, N. K. Sauter and G. Evans, *Acta Crystallogr. D*, **2018**, *74*, 85-97.

6. A. J. McCoy, R. W. Grosse-Kunstleve, P. D. Adams, M. D. Winn, L. C. Storoni and R. J. Read, *J. Appl. Crystallogr.*, **2007**, *40*, 658-674.

7. D. C. Lim, V. Joukov, T. J. Rettenmaier, A. Kumagai, W. G. Dunphy, J. A. Wells and M. B. Yaffe, *Sci. Signal.*, **2020**, *13*, eabb6707.

8. P. Emsley, B. Lohkamp, W. G. Scott and K. Cowtan, *Acta Crystallogr. D*, **2010**, *66*, 486-501.

9. C. J. Williams, J. J. Headd, N. W. Moriarty, M. G. Prisant, L. L. Videau, L. N. Deis, V. Verma, D. A. Keedy, B. J. Hintze, V. B. Chen, S. Jain, S. M. Lewis, W. B. Arendall III, J. Snoeyink, P. D. Adams, S. C. Lovell, J. S. Richardson and D. C. Richardson, *Protein Sci.*, **2018**, *27*, 293-315.

10. R. P. Joosten, F. Long, G. N. Murshudov and A. Perrakis, *IUCrJ*, **2014**, *1*, 213-220.

11. M. C. Chambers, B. Maclean, R. Burke, D. Amodei, D. L. Ruderman, S. Neumann, L. Gatto, B. Fischer, B. Pratt, J. Egertson, K. Hoff, D. Kessner, N. Tasman, N. Shulman, B. Frewen, T. A. Baker, M.-Y. Brusniak, C. Paulse, D. Creasy, L. Flashner, K. Kani, C. Moulding, S. L. Seymour, L. M. Nuwaysir, B. Lefebvre, F. Kuhlmann, J. Roark, P. Rainer, S. Detlev, T. Hemenway, A. Huhmer, J. Langridge, B. Connolly, T. Chadick, K. Holly, J. Eckels, E. W. Deutsch, R. L. Moritz, J. E. Katz, D. B. Agus, M. MacCoss, D. L. Tabb and P. Mallick, A cross-platform toolkit for mass spectrometry and proteomics, *Nat. Biotechnol.*, 2012, **30**, 918-920.
